# The prognostic role of inflammatory markers in patients with metastatic colorectal cancer treated with bevacizumab: A translational study [ASCENT]

**DOI:** 10.1371/journal.pone.0229900

**Published:** 2020-03-06

**Authors:** Stephen John Clarke, Matthew Burge, Kynan Feeney, Peter Gibbs, Kristian Jones, Gavin Marx, Mark P. Molloy, Timothy Price, William H. H. Reece, Eva Segelov, Niall C. Tebbutt

**Affiliations:** 1 Cancer Services, Royal North Shore Hospital, St Leonards, Australia; 2 Cancer Care Services, Royal Brisbane and Women Hospital, and University of Queensland, Herston, Australia; 3 Department of Oncology, Haematology and Palliative Care, St John of God Murdoch Hospital, Murdoch, Australia; 4 Medical Oncology, Western Hospital, Footscray, Australia; 5 Medical Affairs, Roche Products, Pty, Limited, Sydney, Australia; 6 Integrated Cancer Centre, Sydney Adventist Hospital, and University of Sydney, Wahroonga, Australia; 7 Australian Proteome Analysis Facility, Macquarie University, Sydney, Australia; 8 Haematology and Medical Oncology, The Queen Elizabeth Hospital and University of Adelaide, Adelaide, Australia; 9 Biostatistics, Covance Pty Ltd, Sydney, Australia; 10 Medical Oncology, St Vincent’s Hospital, Darlinghurst, Australia; 11 Medical Oncology, Austin Health, Heidelberg, Australia; Istituto di Ricovero e Cura a Carattere Scientifico Centro di Riferimento Oncologico della Basilicata, ITALY

## Abstract

**Background:**

In spite of demonstrating prognostic and possibly predictive benefit in retrospective cohorts and meta-analyses of cancer populations, including colorectal cancer (CRC), prospective evaluation of the relationship between neutrophil to lymphocyte ratio (NLR) and treatment outcomes in previously untreated mCRC patients receiving bevacizumab-based therapy has not yet been performed.

**Methods:**

An open-label, single arm, multi-centre study. Patients received first-line bevacizumab plus XELOX or mFOLFOX6 (Phase-A) and continued bevacizumab plus FOLFIRI beyond first progression (Phase-B). Analyses included the association of NLR with phase A progression free survival (PFS) and overall survival (OS). A sub-study investigated the safety in patients with the primary *in situ* tumor. An exploratory sub-study examined relationships of circulating proteomic markers with PFS.

**Results:**

Phase-A enrolled 128 patients; median age was 64 years (range: 26–84), 70 (55%) were female, 71 (56%) were PS-0 and 51 (40%) had primary *in situ* tumor. Fifty-three (41%) patients entered Phase-B. The median baseline (b) NLR was 3.2 (range: 1.5–20.4) with 32 (25%) patients having bNLR > 5. The PFS hazard ratio (HR) by bNLR > 5 versus ≤ 5 was 1.4 (95% CI: 0.9–2.2; *p =* 0.101). The median PFS was 9.2 months (95% CI: 7.9–10.8) for Phase-A and 6.7 months (95% CI: 3.0–8.2) for Phase-B. The HR for OS based on bNLR > 5 versus ≤ 5 was 1.6 (95% CI: 1.0–2.7; *p =* 0.052). The median OS was 25 months (95% CI: 19.2–29.7) for the full analysis set and 14.9 months for Phase-B. Baseline levels of nine proteomic markers showed a relationship with PFS. Treatment related toxicities were consistent with what has previously been published. There were 4 (3%) instances of GI perforation, of which, 3 (6%) occurred in the primary *in situ* tumor group.

**Conclusions:**

Results from this study are aligned with the previously reported trend towards worse PFS and OS in patients with higher bNLR.

**Trial registration:**

ClinicalTrials.gov: NCT01588990; posted May 1, 2012.

## Background

The efficacy of bevacizumab in combination with fluoropyrimidine-containing chemotherapy has been demonstrated in metastatic colorectal cancer (mCRC).[1–7] However, to date, there are no reproducible, validated, simple and inexpensive prognostic biomarkers to aid treatment selection. The optimal treatment duration and the role of bevacizumab in certain patient subgroups, specifically those considered at particular risk of bevacizumab-mediated toxicity, also require further investigation.

The microenvironment of the tumor and the inflammatory response are considered important effectors of cancer biology and tumorigenesis.[8] Tumor development and progression induced by an inflammatory response are mediated by interactions between pro-inflammatory cytokines and cellular pathways, including the nuclear factor kappa-light-chain-enhancer of activated B cells (NF-ΚB) and the signal transducer and activator of transcription-3 (STAT-3).[9] The role of inflammatory markers as predictive or prognostic tools in the setting of bevacizumab has been investigated retrospectively.[10, 11] The use of blood-based markers such as neutrophil/lymphocyte ratio (NLR) as prognostic/predictive biomarkers in patients receiving bevacizumab-based chemotherapy had not been previously evaluated in this setting.

This study[12] aimed to evaluate the relationship between the host inflammatory response, measured by NLR, selected proteomic plasma markers and treatment outcomes, in patients with previously untreated mCRC receiving bevacizumab-based first- and second-line treatments.

## Methods

### Study design

An open-label, prospective, single arm, phase-IV, multi-center study (NCT01588990) evaluating the relationship between NLR and treatment outcomes in patients with histologically confirmed, previously untreated mCRC; had World Health Organization (WHO) performance status (PS) of 0–1 and life expectancy of ≥ 3 months; and were eligible to received bevacizumab-based, first- and second-line, treatment. The study protocol has previously been published.[12] In summary, there were two phases of treatment; in Phase-A bevacizumab (7.5 mg/kg every 3 weeks) plus XELOX or bevacizumab (5mg/kg every 2 weeks) plus mFOLFOX6 were administered from study start until first disease progression. In Phase-B bevacizumab (5.0 mg/kg every 2 weeks) plus FOLFIRI were administered from first disease progression until second disease progression. The study planned to recruit a total of 150 patients; however, due to competing recruitment only 144 patients were enrolled. The study was conducted in accordance with local guidelines and in line with the principles of the Declaration of Helsinki and Good Clinical Practice Guidelines. Ethics approval was obtained from the following Human Research Ethics committees (HREC): Australian Capital Territory Health HREC (ETH.7.12.168; August 2012); Calvary Health Care Adelaide HREC (Reference number: 12-CHREC-F002; April 2012); Cancer Institute NSW Clinical Research Ethics Committee (HREC/12/CIC/3; May 2012); Central Northern Adelaide Health Service Ethics of Human Research Committee (HREC/12/TQEHLMH/63; October 2012); Melbourne Health HREC (HREC/11/MH/383; March 2012); St John of God Health Care Ethics Committee (HREC#573; October 2012); St Vincent's Hospital HREC (Reference number: 12/109; June 2012); Sydney Adventist Hospital Group HREC (HREC-2012-020; August 2012); Sydney Local Health District Ethics Review Committee (Royal Prince Alfred Hospital Zone; X12-0243; August 2012); and Tasmanian Health and Medical HREC (H12421; May 2012). All participating patients provided written informed consent prior to participation in the study.

Here we present results for the primary endpoint which investigated the prognostic value of the host inflammatory response as assessed by the NLR (≤ 5 versus > 5) on Progression Free Survival (PFS); and the secondary endpoints which investigated the safety profile of bevacizumab, efficacy by treatment phase, the role of NLR as predictor of Overall Survival (OS); the association between post-baseline changes in NLR, PFS and OS. The incidence of serious adverse events related to the primary tumor in the primary *in situ* tumor patient cohort is also presented.

### Proteomic analysis

Pre-treatment, baseline plasma samples were independently prepared in duplicate by reduction and alkylation and then digested overnight at 37°C with trypsin. For each preparation, 1 μg of peptide was analyzed as technical duplicates by nano liquid chromatography-selected reaction monitoring mass spectrometry assays of 66 peptides representing 32 acute phase and inflammation related plasma proteins as previously described.[13, 14] Individual peptide peak areas were obtained following normalization to total peak area, and the means and variances reported.

### Statistical methodologies

The statistical and analytical plan has been previously published.[12] The analysis populations included the Full Analysis Set (FAS), defined as subjects who received at least one dose of bevacizumab; the “primary *in situ* population” was defined as all subjects in the FAS with a primary *in situ* tumor; and the “resected primary tumor population” was defined as all subjects in the FAS without a primary *in situ* tumor. PFS was defined as the time from the start of initial treatment to documentation of first disease progression or death from any cause, whichever occurred first. PFS in Phase-B was defined as the time from the start of Phase-B treatment to documentation of second disease progression or death from any cause, whichever occurred first. OS was defined as the time from the start of initial treatment to the date of death, regardless of the cause of death. OS in Phase-B was defined as the time from the start of treatment in Phase-B to death of any cause.

All analyses were conducted (using the Statistical Analysis System, SAS v9.3) on the FAS unless otherwise stated. Testing of statistical hypotheses was conducted at two-sided alpha of 0.05.

The NLR at the start of treatment was dichotomized between ≤ 5 and > 5 and tested in a standard Proportional Hazards Cox regression model [15] for an association with PFS (primary analysis) and OS (secondary analysis), in a model adjusted for the default covariates, which included the WHO PS (0 versus. 1), metastatic disease in the liver (Yes/No), number of different sites of metastatic disease (≤ 3 versus. > 3), and presence of metastatic disease in the liver with no other sites involved (Yes/No). The association between longitudinal NLR measurements and PFS and OS was assessed by including NLR (≤ 5 versus > 5) as a time-varying covariate in the primary model, adjusted for all the covariates that were defined as covariates in the primary model.

## Results

### Patients

**Fig 1:**
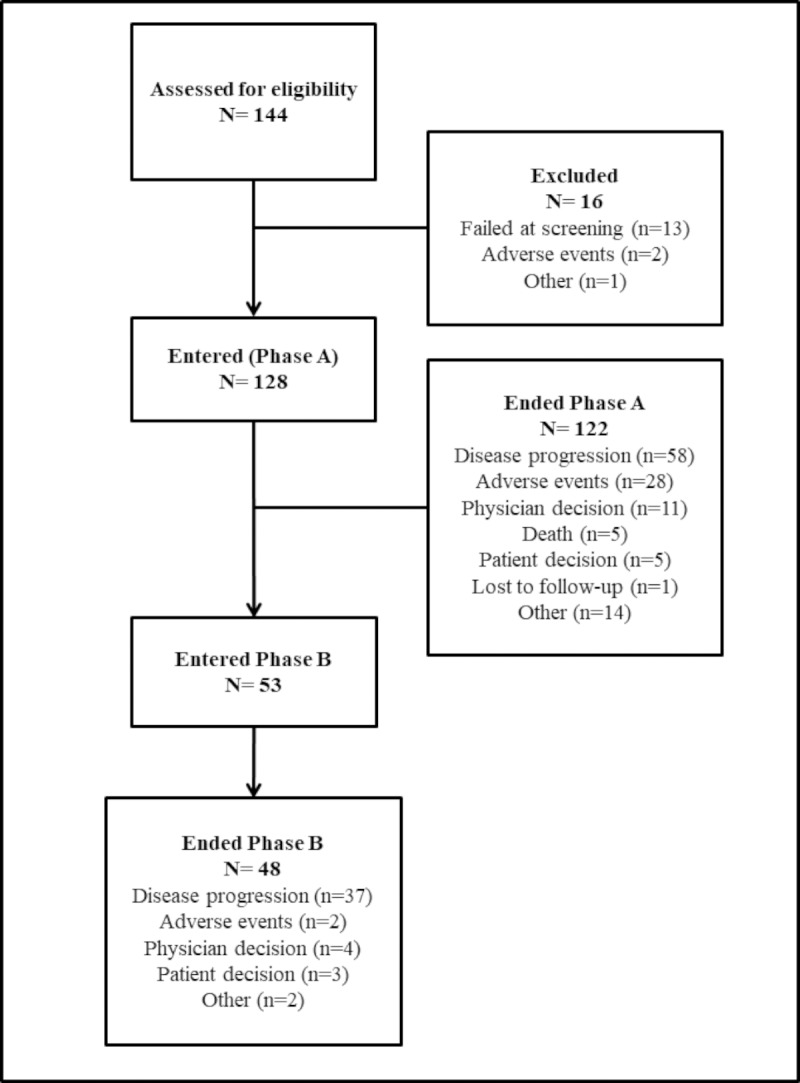
Patient disposition.

A total of 144 patients were screened (signed the informed consent) from 17 sites; 16 patients were excluded (Fig 1). A total of 128 patients were enrolled in Phase-A (June-2012 to September-2016) and received treatment. Fifty-eight patients ended Phase-A due to disease progression; of these 53 patients continued into Phase-B (Fig 1). The median age of the overall population was 64 years (range: 26–84). There were more female (*n* = 70; 55%; Table 1). The majority (*n* = 100; 78%) had a Charlson comorbidity index ≤ 1. Seventy-one patients (56%) had WHO PS 0 and 56 (44%) patients had PS of 1. Overall, 96 (75%) patients had baseline (b) NLR ≤ 5 while 32 (25%) patients had bNRL > 5, with median bNLR of 3 (range: 1.2–20.0). Fifty-one patients had primary *in situ* tumor and 77 had resected primary tumor.

**Table 1:** Summary of demographics and patient characteristics at baseline (full analysis set population).

	NLR ≤ 5 (N = 96)	NLR > 5 (N = 32)	Total (N = 128)
**Age (years)**			
n	96	32	128
median (min; max)	64 (31; 82)	63 (26; 84)	64 (26;84)
**Gender, (n, %)**			
Male	44 (46)	14 (44)	58 (45)
Female	52 (54)	18 (56)	70 (55)
**Tumor Status (n, %)**			
Primary in situ (PIS)	33 (34)	18 (56)	51 (40)
Resected primary tumor (RPT)	63 (66)	14 (44)	77 (60)
**WHO Performance Status (n, %)**			
Missing	1 (1)	0	1 (1)
0	56 (58)	15 (47)	71 (56)
≥ 1	39 (41)	17 (53)	56 (44)
**Charlson Comorbidity Index (n, %)**			
Missing	3 (3)	2 (6)	5 (4)
≤ 1	73 (76)	27 (84)	100 (78)
> 1	20 (20)	3 (9)	23 (18)
**NLR**			
Mean (SD)	3 (1)	9 (3)	4 (3)
Median (range	3 (1–5)	8 (5–20)	3 (1–20)
**PLR (n, %)**			
≤ 150	39 (41)	2 (6)	41 (32)
> 150 and ≤ 300	52 (54)	10 (31)	62 (48)
> 300	5 (5)	20 (63)	25 (20)
**Glasgow Prognostic Index (n, %)**			
0	29 (30)	0	29 (23)
1	37 (39)	0	37 (29)
2	13 (14)	2 (6)	15 (12)
3	16 (17)	11 (34)	27 (21)
4	1 (1)	12 (38)	13 (10)
5	0	7 (22)	7 (6)
**Site of Metastatic Disease (n (%))**			
Liver	65 (68)	24 (75)	89 (70)
Lung	37 (39)	16 (50)	53 (41)
Bone	8 (8)	8 (25)	16 (13)
Lymph nodes	40 (42)	14 (44)	54 (42)
Peritoneal/omental fat	15 (16)	6 (19)	21 (16)

Abbreviations: max = maximum; min = minimum; NLR = neutrophil/lymphocyte ratio, PLR = platelet/lymphocyte ratio; SD = standard deviation.

### Treatment exposure

Patients received median 15 cycles (range: 1–91) of bevacizumab treatment; 11 cycles (range: 1–91) in Phase-A and 8 cycles (range: 1–79) in Phase-B. At study end, 6 patients remained on treatment in Phase-A and 5 patients in Phase-B. Bevacizumab dose interruption due to adverse events (AEs) were recorded in 78 (61%) patients overall; 70 (55%) patients in Phase-A and 20 (38%) patients in Phase-B. The major reasons for treatment discontinuation were disease progression (*n* = 58; 45%) and AEs (*n* = 28; 22%) in Phase-A and disease progression (*n* = 37; 70%) in Phase-B (Fig 1).

XELOX was administered in 38 patients in Phase-A, with median 32 weeks (range: 3–189). Eighty patients received mFOLFOX6; median number of weeks was 23 (range: 2–189). In Phase-B, 53 patients received a median of 18 weeks of FOLFIRI (range: 0–198).

### Efficacy analyses

Using the Cox proportional hazards model (Table 2) for the primary analyses, NLR was not shown to be prognostic of PFS; the Hazard Ratio (HR) [NLR > 5 versus NLR ≤ 5] was 1.4 (95% CI: 0.9–2.2; *p* = 0.101). An HR of 1.4 equates to a predicted difference in 12-month PFS from 56% for patient with bNLR ≤ 5 vs 44%, had that same patient had bNLR >5 (S1 Table).

**Table 2:** Multivariate cox proportional hazards analysis of the influence of baseline characteristics on PFS and OS (full analysis set).

	Progression Free Survival			Overall Survival		
Covariate	HR	95% CI	*p*	HR	95% CI	*p*
**NLR (> 5 versus ≤ 5)**	1.4	0.9,2.2	0.101	1.6	1.0, 2.7	0.052
**WHO PS (≥ 1 versus 0)**	1.6	1.1, 2.4	0.013	1.8	1.1, 2.8	0.011
**Metastatic liver disease (yes versus no)**	1.5	1.0, 2.4	0.079	1.4	0.8, 2.4	0.206
**Number of metastatic sites (> 3 versus ≤ 3)**	1.0	0.5, 2.0	0.886	1.5	0.8, 3.1	0.219
**Metastatic liver disease with no other sites (yes versus no)**	1.0	0.6, 1.7	0.916	0.8	0.4, 1.5	0.440

The HR for OS (bNLR > 5 versus ≤ 5) was 1.6 (95% CI: 1.0–2.7; *p* = 0.052). The predicted OS probability at 12 months from the primary model for subjects with WHO PS 0, no metastatic liver disease and ≤3 sites of metastatic disease, was 87% for subjects with bNLR ≤ 5 and 79% for subjects with bNLR > 5 (S2 Table).

To determine whether post baseline normalization of NLR was a determinant of response to treatment, the longitudinal NLR was added as a time-varying covariate to the primary model. The model generated a PFS HR = 1.3 (95% CI: 0.9–1.9; *p* = 0.188) and an OS HR = 2.2 (95% CI: 1.2–4.0; *p* = 0.016). While these results do not support the hypothesis that normalization of NLR is a predictor of response, they suggest that NLR status of the patient at any time is associated with an increased rate of mortality.

To determine whether any of the baseline characteristics or other laboratory values were confounding for the effect of NLR, the demographic and laboratory values were each individually included in the primary model (S3 Table). None of these changed the association to being statistically significant. Only the inclusion of the Glasgow Prognostic Index as a linear variable changed the direction of the association, but the effect was still not statistically significant.

Time to event measures for each of the following secondary outcome variables, PFS in Phase-A and Phase-B, OS in the FAS population and Phase-B OS are summarised in Table 3.

**Table 3:** Secondary efficacy analyses.

Outcome (population)	N	Median (mo) (95% CI)	Range (min, max)	12-mo predicted Proportion (95% CI)
**PFS until 1^st^ Progression (FAS)**	128	9.2 (7.9, 10.8)	1.0, 42.5	35.8% (27.4, 44.3)
**OS (FAS)**	128	25.0 (19.2, 29.7)	1.0, 47.5	75.6% (67.0, 82.3)
**Survival beyond first progression (Progressed in Phase-A)**	101	12.6 (8.8, 15.9)	0.2, 45.7	52.0% (41.4, 61.6)
**PFS (Phase B)**	53	6.7 (3.0, 8.2)	0.0, 44.1	15.9% (7.1, 28.1)
**OS (Phase B)**	53	14.9 (8.2, 17.5)	0.0, 44.8	59.4% (44.0, 71.9)

Abbreviations: FAS = full analysis set; mo = Months; PFS = Progression Free Survival; OS = Overall Survival

### Safety analyses

All 128 patients experienced at least one AE. Most patients experienced mild (*n* = 125; 98%) or moderate (*n* = 119; 93%) AEs. Grade 3–5 AEs were reported in 102 (79.7%) patients overall; 97 (75.8%) patients in Phase-A and 24 (45.3%) patients in Phase-B. The incidence of the most frequent AEs by primary tumor status is presented in Table 4.

**Table 4:** Summary of non-serious adverse events reported by ≥ 20% of patients (full analysis set).

Preferred Term	PIS (N = 51) n (%)	RPT (N = 77) n (%)	Total (N = 128) n (%)
**Nausea**	37 (72.5)	50 (64.9)	87 (68.0)
**Fatigue**	33 (64.7)	47 (61.0)	80 (62.5)
**Neuropathy peripheral**	28 (54.9)	52 (67.5)	80 (62.5)
**Diarrhea**	28 (54.9)	44 (57.1)	72 (56.3)
**Constipation**	26 (51.0)	26 (33.8)	52 (40.6)
**Abdominal pain**	14 (27.5)	30 (39.0)	44 (34.4)
**Palmar-plantar erythrodysaesthesia syndrome**	14 (27.5)	24 (31.2)	38 (29.7)
**Vomiting**	14 (27.5)	26 (33.8)	40 (31.3)
**Neutropenia**	15 (29.4)	18 (23.4)	33 (25.8)
**Mucosal inflammation**	14 (27.5)	17 (22.1)	31 (24.2)
**Epistaxis**	9 (17.6)	21 (27.3)	30 (23.4)
**Decreased appetite**	16 (31.4)	12 (15.6)	28 (21.9)
**Gastroesophageal reflux disease**	15 (29.4)	12 (15.6)	27 (21.1)
**Paraesthesia**	10 (19.6)	16 (20.8)	26 (20.3)
**Alopecia**	7 (13.7)	19 (24.7)	26 (20.3)

Abbreviations: NLR = neutrophil/lymphocyte ratio; PIS = primary in situ tumor; RPT = resected primary tumor. The denominator for percentages is the number of patients in the FAS for each Primary Intact or Resected group. Sorted in descending order of frequency based on the total column. Note: This table contains counts of subjects. If a subject experienced more than one episode of an adverse event, the subject is counted only once within a preferred term.

Of particular interest within the study context, any grade anal abscess and enterovesical fistula were reported in two patients each; and anal fistula, gastrointestinal perforation, intestinal perforation, large intestine perforation and rectal perforation in one patient each. Anal abscess, enterovesical fistula, and anal fistula were mostly reported in primary *in situ* tumor patients (4 of 5 cases) and in patients with baseline NLR ≤ 5 (4 of 5 cases). Similarly, gastro-intestinal perforations were mostly observed in patients with primary *in situ* tumor (3 of 4 cases) and in patients with baseline NLR ≤ 5 (3 of 4 cases).

AEs possibly related to bevacizumab were reported in 88 (69%) patients; 38 (30%) patients experienced at least 1 Grade 3–5 AE possibly related to bevacizumab, the most frequently reported of which were pulmonary embolism (*n* = 13; 10%) and hypertension (*n* = 8; 6%), followed by neutropenia (*n* = 7; 6%), and proteinuria (*n* = 3; 2%).

### Death on study

Eighty-two subjects died on study, the majority due to disease progression. Fatal AEs were reported in 7 (6%) patients and included acute renal failure, intestinal obstruction, pulmonary embolism, pneumonia, and sepsis in the primary *in situ* tumor population, and gangrene and aspiration pneumonia in the resected primary tumor population. The pulmonary embolism was considered related to bevacizumab.

### Proteomic analysis

Fifty-one (40%) patients were included in the proteomic analyses. We examined whether the baseline abundances of 32 high-medium abundance plasma proteins had a relationship with PFS (S4 Table). Nine acute phase reactants were found to be significantly related with PFS (*p* < 0.05) after adjusting for the effect of NLR, and warrant further investigation in larger cohorts: A1AGLP [HR = 1.13 (95% CI: 1.00–1.28; *p* = 0.047)], A1MICG [HR = 0.09 (95% CI: 0.01–0.99; *p =* 0.049)] AACT [HR = 1.62 (95% CI: 1.04–2.53; *p* = 0.033)], APOC3 [HR = 0.77 (95% CI: 0.60–1.00; *p* = 0.049)], CRLPLSMN [HR = 14.65 (95% CI: 1.06–202.32; *p* = 0.045)], CRP (Logged and raw) [HR = 1.48 (95% CI: 1.09–2.02; *p* = 0.013], FIBB [HR = 0.80 (95% CI: 0.67–0.96; *p* = 0.014)], KNG1 [HR = 0 (95% CI: 0.00–0.49; *p* = 0.024)] and PREALB [HR = 0.01 (95% CI: 0.00–0.59; *p* = 0.027).

There were three Grade 3–5 AEs in the first cycle amongst patients in the proteomic sub-study population, therefore significance of the proteomic markers to predict Grade 3–5 AEs in the first cycle could not be assessed.

## Discussion

This multi-center study was the first to prospectively evaluate the relationship between the host inflammatory response, measured by NLR, and outcomes in subjects with previously untreated mCRC who received bevacizumab-based first- and second-line treatment. Progression was based not on the RECIST criteria, but on investigators’ routine clinical assessment which enabled the primary endpoint to reflect the clinical course of disease under routine clinical practice. The observed median PFS and OS in both phases of our study is consistent with the published literature, reporting that patients continue to derive benefits from bevacizumab when used beyond progression.[16–18]

Although this study is significantly smaller than the retrospective, published, literature on NLR in other cancers, it did have enough power to detect a HR of 1.7 [10]. While our results did not prove the claim that NLR > 5 is prognostic for lower PFS [10, 19], the size of the association (HR of 1.4) is consistent with data published by Chua *et al* [10] and other subsequent data.[20, 21]

The observed association between longitudinal NLR measurements and OS [HR = 2.2 (95% CI: 1.2–4.0; *p* = 0.016)] indicates that NLR status of the patient at any time is associated with an increased rate of mortality and warrants further investigation. Results were after adjustment for the baseline disease characteristics included in the primary model. This is consistent with results from a recent meta-analysis [21] which analysed data from 9363 colorectal cancer patients and showed that elevated NLR was a negative predictor of outcome.[22]

The detected associations of PFS with the A1AGLP, A1MICG, AACT, APOC3, CRLPLSMN, CRP (Logged and raw), FIBB, KNG1 and PREALB proteomic markers were found through post-hoc exploratory analysis. Further validation of the associations is required, especially interesting for those proteins where large effects were observed (CRLPLSMN, KING1, PREALB).

The safety profile observed in the study was consistent with the known safety profile of bevacizumab, with no apparent differences between the primary *in situ* tumor and resected primary tumor populations. The numbers of reported perforations were not higher than the published data.

## Conclusion

Although this study did not confirm the prognostic value of NLR in metastatic colorectal cancer patients, treated with bevacizumab, there was a trend towards worse PFS and OS with higher bNLR which is consistent with previous studies.[21]. Treatment related toxicities were consistent with prior experience with no apparent differences between the primary *in situ* tumor and resected primary tumor populations. No new safety signals were reported for this study.

## Supporting information

S1 TREND checklist TREND statement checklist. (PDF)

S1 File (PDF)

S1 Table Predicted PFS probabilities from the primary model (full analysis set). (DOCX)

S2 Table Predicted overall survival probabilities from the primary model (full analysis set). (DOCX)

S3 Table Impact of potential confounders on the primary endpoint (full analysis set). (DOCX)

S4 Table The association between baseline proteomic markers and PFS (full analysis set). (DOCX)

## List of abbreviations

AACT: Acetoacetyl-coenzyme A thiolase
AE: Adverse events
A1AGLP: Alpha-1 acid glycoprotein
A1MICG: Alpha-1 microglobulin
APOC3: Apolipoprotein C-III
CRLPLSMN: Ceruloplasmin
CRP: C-reactive protein
FAS: Full analysis set
FIBB: Fibrinogen binding protein
FOLFIRI: Folinic acid, fluorouracil, irinotecan regimen
HR: Hazards ratio
KNG1: Kininogen 1
mCRC: metastatic colorectal cancer
mFOLFOX6: Modified folinic acid, fluorouracil, oxaliplatin regimen
NF-ΚB: Nuclear factor kappa-light-chain-enhancer of activated B cells
NLR: Neutrophil/lymphocyte ratio
OS: Overall survival
PFS: Progression free survival
PREALB: Prealbumin
RECIST: Response evaluation criteria in solid tumors
STAT-3: signal transducer and activator of transcription-3
WHO: World health organization
XELOX: Capecitabine plus oxaliplatin regimen
