# Supplementary material for: The prognostic role of inflammatory markers in patients with metastatic colorectal cancer treated with bevacizumab: A translational study [ASCENT]
Source: PLoS One. 2020 Mar 6;15(3):e0229900. doi: 10.1371/journal.pone.0229900 (PMC7059922; doi:10.1371/journal.pone.0229900)
Supplement: S1 File — (PDF) [file pone.0229900.s002.pdf]

ROCHE PRODUCTS PTY LIMITED
CLINICAL STUDY PROTOCOL

An <u>A</u>ustralian Translational <u>S</u>tudy to Evaluate the Prognostic Role of Inflammatory Markers in Patients with Metastatic <u>C</u>olor<u>e</u>ctal Ca<u>n</u>cer <u>T</u>reated with Bevacizumab (Avastin™) [ASCENT]

PROTOCOL NUMBER ML25753
ASCENT

AVASTIN® (BEVACIZUMAB)
VERSION – 1.2

Sponsor: Roche Products Pty Limited

PROTOCOL APPROVAL	
Protocol Number / Version:	ML25753 / Version 1.2
Date: 27th November 2012	
Protocol approved by:	
[redacted]<br>Roche Products Pty Limited, Australia<br>Date of approval 27-Nov-2012	[redacted]
[redacted] PhD, MBiostat<br>Statistician<br>[redacted], Australia<br>Date of approval 17 Dec 2012	[redacted] 17 Dec 2012

[redacted]

PROTOCOL ACCEPTANCE FORM

**TITLE:**	ASCENT
**PROTOCOL NUMBER:**	ML25753
**VERSION NUMBER:**	1.2 Dated 27 November 2012
**TEST PRODUCT:**	Bevacizumab RO4876646
**MEDICAL MONITOR:**	Dr [redacted]
**SPONSOR:**	Roche Products Pty Limited

I agree to conduct the study in accordance with the current protocol.

____

Principal Investigator's Name (print)

____ ________________

Principal Investigator's Signature Date

Please return a copy of the form as instructed by your local study monitor.

Table of contents

ML25753 STUDY SYNOPSIS **10**

1. BACKGROUND AND RATIONALE **25**

1.1 Colorectal Cancer 25
1.2 Bevacizumab 25
1.2.1 Bevacizumab in mCRC *26*
1.2.2 Bevacizumab beyond progression (BBP) *26*
1.2.3 Bevacizumab in patients with a primary in situ tumor *26*
1.3 Inflammation as a potential biomarker for bevacizumab in mCRC 27
1.3.1 Inflammation-based biomarkers *29*
1.3.2 Neutrophil:lymphocyte ratio and mCRC treatment outcomes *30*
1.4 Rationale for the Study 30

2. OBJECTIVES **31**

2.1 Primary Objective 31
2.2 Secondary Objectives 31
2.3 Exploratory Objectives 34

3. STUDY DESIGN **35**

3.1 Overview of Study Design 35
3.1.1 Primary In Situ Subgroup *36*
3.1.2 Rationale for Study Design *37*
3.1.3 Rationale for Dose Selection *37*
3.1.4 Study Duration *38*
3.1.5 End of Study *38*
3.2 Number of Subjects 38
3.3 Centers 38

4. STUDY POPULATION **38**

4.1 Overview 38
4.2 Inclusion Criteria 38
4.3 Exclusion Criteria 39
4.4 Concomitant Medication and Treatment 41
4.4.1 Antineoplastic treatments *41*
4.4.2 Surgery *41*
4.4.3 Radiotherapy *41*
4.4.4 Anticoagulant and anti-platelet therapies *42*
4.5 Criteria for Premature Withdrawal 43
4.6 Replacement Policy 43
4.6.1 For Patients *43*
4.6.2 For Centers *43*

5. SCHEDULE OF ASSESSMENTS AND PROCEDURES **45**

5.1 Screening Examination and Eligibility Screening Form 49
5.2 Procedures for Enrollment of Eligible Subjects 49
5.3 Clinical Assessments and Procedures 49
5.3.1 Screening Procedures *49*
5.3.2 Baseline Procedures *50*
5.3.3 Treatment Period *51*

5.3.4 *Safety follow-up visit* ... 53
5.3.5 *Survival follow-up visits* ... 54
5.3.6 *Tumor Response and Disease Progression* ... 55
5.3.7 *Performance status* ... 56
5.3.8 *Clinical Safety Assessments* ... 57
5.4 LABORATORY ASSESSMENTS ... 57
5.4.1 *Standard laboratory assessments* ... 57
5.4.2 *Plasma samples for proteomic sub-study* ... 58
5.4.3 *Tumor Tissue Banking* ... 58
5.5 PATIENT REPORTED OUTCOME(S) ... 58
5.5.1 *Quality of Life Assessments* ... 58

6. INVESTIGATIONAL MEDICINAL PRODUCT ... 59

6.1 DOSE AND SCHEDULE OF IMP ... 59
6.1.1 *Phase A* ... 59
6.1.2 *Phase B* ... 60
6.2 DOSE MODIFICATIONS, INTERRUPTIONS AND DELAYS ... 61
6.2.1 *Bevacizumab* ... 61
6.2.2 *Drug holidays* ... 65
6.2.3 *Bevacizumab monotherapy* ... 65
6.2.4 *Surgical procedures/wound healing complications* ... 66
6.2.5 *Infusion Reactions* ... 66
6.2.6 *Chemotherapy* ... 66
6.3 PREPARATION AND ADMINISTRATION OF IMP ... 68
6.3.1 *Preparation of Bevacizumab* ... 68
6.3.2 *Administration of Bevacizumab* ... 69
6.3.3 *Bevacizumab Storage* ... 69
6.3.4 *Incompatibilities* ... 69
6.4 FORMULATION, PACKAGING AND LABELING ... 70
6.5 BLINDING AND UNBLINDING ... 70
6.6 ACCOUNTABILITY OF IMP AND ASSESSMENT OF COMPLIANCE ... 70
6.6.1 *Accountability of IMP* ... 70
6.6.2 *Assessment of Compliance* ... 71
6.7 DESTRUCTION OF THE IMP ... 71

7. SAFETY INSTRUCTIONS AND GUIDANCE ... 71

7.1 ADVERSE EVENTS AND LABORATORY ABNORMALITIES ... 71
7.1.1 *Clinical AEs* ... 71
7.1.2 *Treatment and Follow-up of AEs* ... 74
7.1.3 *Laboratory Test Abnormalities* ... 75
7.1.4 *Toxicity Management Guidelines* ... 75
7.2 HANDLING OF SAFETY PARAMETERS ... 75
7.2.1 *Reporting of Serious Adverse Events (immediately reportable)* ... 75
7.2.2 *Reporting of Non-serious Events of Special Interest* ... 77
7.2.3 *Pregnancy* ... 77
7.3 WARNINGS AND PRECAUTIONS ... 77

8. STATISTICAL CONSIDERATIONS AND ANALYTICAL PLAN ... 78

8.1 PRIMARY VARIABLE ... 78
8.2 SECONDARY VARIABLES ... 78
8.2.1 *Secondary Safety Variables* ... 78
8.2.2 *Secondary Efficacy variables* ... 79

8.3 STATISTICAL AND ANALYTICAL METHODS 80
8.3.1 Statistical Method *80*
8.3.2 Primary Analysis *81*
8.3.3 Safety Data Analysis *81*
8.3.4 Other Analyses *82*
8.4 SAMPLE SIZE 83

9. DATA COLLECTION, MANAGEMENT AND QUALITY ASSURANCE 83

9.1 ASSIGNMENT OF PREFERRED TERMS AND ORIGINAL TERMINOLOGY 83

10. ETHICAL ASPECTS 84

10.1 LOCAL REGULATIONS/DECLARATION OF HELSINKI 84
10.2 INFORMED CONSENT 84
10.3 INDEPENDENT ETHICS COMMITTEES (IEC) 84
10.4 ROLE OF THE SCIENCE ADVISORY COMMITTEE (SAC) 85
10.5 FINANCIAL DISCLOSURE 85

11. CONDITIONS FOR MODIFYING THE PROTOCOL 85

12. CONDITIONS FOR TERMINATING THE STUDY 86

13. STUDY DOCUMENTATION, ECRFS AND RECORD KEEPING 86

13.1 INVESTIGATOR'S FILES / RETENTION OF DOCUMENTS 86
13.2 SOURCE DOCUMENTS AND BACKGROUND DATA 87
13.3 AUDITS AND INSPECTIONS 87
13.4 CASE REPORT FORMS OR ELECTRONIC CASE REPORT FORMS 87

14. MONITORING THE STUDY 87

15. CONFIDENTIALITY OF TRIAL DOCUMENTS AND SUBJECT RECORDS 88

16. CLINICAL STUDY REPORT (CSR) 88

17. PUBLICATION OF DATA AND PROTECTION OF TRADE SECRETS 88

17.1 CRITERIA FOR AUTHORSHIP 89
17.2 ML25753 PUBLICATION POLICIES AND RULES 89

18. APPENDICES 90

18.1 PROTEOMIC SUB-STUDY 90
18.2 ICH GUIDELINES FOR CLINICAL SAFETY DATA MANAGEMENT, DEFINITIONS AND STANDARDS FOR EXPEDITED REPORTING, TOPIC E2 93
18.3 WHO PERFORMANCE STATUS 95
18.4 EQ-5D 96
18.5 AQOL 8-D 98
18.6 FACT-C (VERSION 4) 99
18.7 NYHA CLASSIFICATION OF HEART FAILURE 102
18.8 COMMON TERMINOLOGY CRITERIA FOR ADVERSE EVENTS V4.0 103
18.9 SUGGESTED TREATMENT ALGORITHM FOR THE MANAGEMENT OF BEVACIZUMAB-INDUCED HYPERTENSION 104
18.10 PAIRING ANTIHYPERTENSIVE AGENTS 105
18.11 CAPECITABINE DOSE CALCULATION ACCORDING TO BODY SURFACE AREA (BSA) 106
18.12 GENERAL GUIDANCE FOR DOSE MODIFICATIONS OF IRINOTECAN/5-FU 107
18.13 MODIFIED CHARLSON COMORBIDITY INDEX 108

19. REFERENCES 109

GLOSSARY OF ABBREVIATIONS

AE	Adverse Event
ALP	Alkaline Phosphatase
ALT (SGPT)	Alanine Aminotransferase
ANC	Absolute Neutrophil Count
aPTT	Activated Partial Thromboplastin Time
AST (SGOT)	Aspartate Aminotransferase
ATE	Arterial Thromboembolic Event
BBP	Bevacizumab Beyond Progression
BP	Blood Pressure
BSA	Body Surface Area
BUN	Blood Urea Nitrogen
CEA	Carcinoembryonic Antigen
CHF	Congestive Heart Failure
CI	Confidence Interval
CNS	Central Nervous System
CR	Complete Response
CRP	C-reactive Protein
CSR	Clinical Study Report
CT	Computer Tomography
CVAD	Central Venous Access Device
CXR	Chest X-Ray
DDC	Duration of Disease Control
ECG	Electrocardiogram
eCRF	Electronic Case Report Form
EDC	Electronic Data Capture
EDTA	Ethylenediaminetetraacetic Acid
EEG	Electroencephalogram
EGF	Epidermal Growth Factor

EGFR	Epidermal Growth Factor Receptor
EMEA	European Agency for the Evaluation of Medicinal Products
EoT	End of Treatment
ESF	Eligibility Screening Form
ESMO	European Society for Medical Oncology
EU	European Union
FACIT	Functional Assessment of Chronic Illness Therapy
FDA	Food and Drug Administration
FOLFIRI	Infusional 5-Fluoarouracil, Leucovorin and Irinotecan
GCP	Good Clinical Practice
G-CSF	Granulocyte Colony-Stimulating Factor
GM-CSF	Granulocyte-Macrophage Colony-Stimulating Factor
GERCOR	Groupe Cooperateur Multidisciplinaire en Oncologie
GGT	Gamma Glutamyl Transferase
GI	Gastrointestinal
GPS	Glasgow Prognostic Score
HPLC	High Performance Liquid Chromatography
HR	Hazard Ratio
ICH	International Conference on Harmonization
ICMJE	International Committee of Medical Journal Editors
IHC	Immunohistochemistry
INN	International Non-proprietary Name
INR	International Normalized Ratio
IRB/IEC	Institutional Review Board/Independent Ethics Committee
iv	Intravenous
LDH	Lactate Dehydrogenase
LMWH	Low Molecular Weight Heparin
mCRC	Metastatic Colorectal Cancer
MedDRA	Medical Dictionary for Regulatory Activities
mFOLFOX6	Modified infusional 5-Fluorouracil, Leucovorin and Oxaliplatin (2 weekly schedule)
mGPS	Modified Glasgow Prognostic Score
MTD	Maximum Tolerated Dose

MRI	Magnetic Resonance Imaging
NCDB	National Cancer Data Base
NCI	National Cancer Institute
NCI-CTC	National Cancer Institute-Common Toxicity Criteria
NCI-CTCAE	National Cancer Institute-Common Toxicity Criteria for Adverse Events
NCIC-CTG	National Cancer Institute of Canada Clinical Trials Group
NLR	Neutrophil/Lymphocyte Ratio
NYHA	New York Heart Association
ORR	Overall Response Rate
OS	Overall Survival
OS-B	Overall Survival during phase B
PBS	Pharmaceutical Benefits Scheme
PD	Progressive Disease
PE	Pharmacoeconomic
PFS	Progression Free Survival
PFS-B	Progression Free Survival during Phase B
PLR	Platelet/Lymphocyte Ratio
PS	Performance Status
PK	Pharmacokinetic
p.o.	Oral Administration
PR	Pulse Rate
PR	Partial Response
QoL	Quality of Life
RBC	Red Blood Cell
RECIST	Response Evaluation Criteria in Solid Tumors
RPLS	Reversible Posterior Leukoencephalopathy Syndrome
SAE	Serious Adverse Event
SAC	Scientific Advisory Committee
SBP	Survival Beyond Progression
SD	Stable Disease
SMT	Study Management Team
SSR	Six Monthly Safety Report

TA	Therapeutic Dose of Anticoagulation Treatment
TE Fistula	Tracheoesophageal Fistula
TFS	Time to Failure of Strategy
TMA	Tissue Microarray
TTF	Time to Treatment Failure
TTP	Time to Tumor Progression
ULN	Upper Limit of Normal
UPC	Urine Protein:Creatinine
VEGF	Vascular Endothelial Growth Factor
VTE	Venous Thromboembolic Event
WBC	White Blood Cell
WHO	World Health Organization
XELOX	Oral Capecitabine plus Infusional Oxaliplatin

ML25753 STUDY SYNOPSIS

**TITLE**	An **A**ustralian Translational **S**tudy to Evaluate the Prognostic Role of Inflammatory Markers in Patients with Metastatic **C**olor**e**ctal Ca**n**cer **T**reated with Bevacizumab (Avastin™) [ASCENT]
**SPONSOR**	Roche Products Pty Limited
**CLINICAL PHASE**	Phase IV
**INDICATION (for study drug)**	Patients with previously untreated metastatic colorectal cancer (mCRC) with a WHO performance status of 0 or 1 who are not candidates for curative resection and are candidates for oxaliplatin-based chemotherapy
**OBJECTIVES:**	
**PRIMARY**	The primary objective of the study is to assess the prognostic value of the host inflammatory response as assessed by the Neutrophil:Lymphocyte Ratio (NLR ≤ 5 vs > 5) on Progression Free Survival.
**SECONDARY**	To further characterize the safety profile of study treatment and evaluate its efficacy following treatment initiation, initial response and when continued after progression.<br>• To assess effectiveness outcomes as measured by :<br>– Progression-free survival until 1st progression (PFS)<br>– Progression-free survival during Phase B (PFS-B)<br>– Time to failure of strategy (TFS)<br>– Duration of disease control (DDC)<br>– Overall survival from the start of treatment (OS)<br>– Survival beyond 1st progression (SBP)<br>– Overall survival during Phase B (OS-B)<br>– Best overall response rate (ORR) from the start of treatment as assessed by the investigator in each treatment phase<br>– Rate of liver resection<br>• To assess the overall incidence of adverse events, including:<br>– Relatedness to bevacizumab<br>– Severity (CTCAE Grade 3-5)<br>– Seriousness<br>– Adverse events of special interest<br>• To validate the NLR as a predictor of OS in patients treated with bevacizumab<br>• To assess the association between post-baseline changes in NLR and PFS and OS

	• In the subgroup of patients with a primary in situ tumor, to assess the incidence of serious adverse events related to the primary in situ tumor. These include, but are not limited to, the following:<br>– Colonic bleeding requiring surgical intervention<br>– Perforation (contained or free) requiring surgery<br>– Bowel obstruction requiring surgery<br>– Fistula formation requiring surgery<br>– Any events related to the primary in situ tumor resulting in patient death<br>– Colonic obstruction not requiring surgery<br>a) Requiring hospitalization for medical management<br>b) Requiring stent placement, laser treatment or fulguration<br>– Gastrointestinal (GI) bleeding requiring transfusion<br>– Fistula formation not requiring surgery<br>a) Self-draining entercutaneous fistula<br>b) Intra-abdominal abscess requiring percutaneous drainage<br>– Other events related to the primary tumor requiring hospitalization, but not surgery<br>• To assess patient reported Quality of Life (QoL)
**EXPLORATORY**	• To further characterize the relationship between blood-based markers of systemic inflammation and therapeutic outcomes in mCRC patients treated with bevacizumab-based treatment.<br>• To further characterize the influence of standard biochemical parameters on therapeutic outcomes, including baseline and ongoing changes.<br>• To further characterize markers of IL-6 mediated inflammatory pathways, angiogenesis and other possible markers considered relevant to efficacy or safety outcomes.
**TRIAL DESIGN**	This is an open-label, prospective, single arm, phase IV, Australian multi-center study evaluating the relationship between the host inflammatory response as measured by NLR and treatment outcomes in patients with previously untreated mCRC who will receive bevacizumab-based first- and second-line treatment.<br><br>The trial consists of two phases of treatment:

- *Phase A treatment*: XELOX or mFOLFOX6 plus bevacizumab administered from study start until 1st disease progression;
- *Phase B treatment*: FOLFIRI plus bevacizumab administered from 1st disease progression until 2nd disease progression.

Bevacizumab infusions will be administered on a three-weekly basis in combination with XELOX or on a two-weekly basis in combination with mFOLFOX6 throughout Phase A treatment until 1st disease progression or occurrence of unmanageable toxicity. Upon documented disease progression, Phase A treatment will be discontinued and bevacizumab will be continued on a two-weekly basis in combination with FOLFIRI (Phase B treatment) until 2nd disease progression or unmanageable toxicity. Upon 2nd disease progression, all study treatment will be discontinued and patients will enter follow-up.

Phase B treatment (bevacizumab plus FOLFIRI) must commence within 4 weeks of the date of documented 1st disease progression.

Patients will attend study specific visits every 8 or 9 weeks (to coincide with chemotherapy regimen) throughout Phase A and B. All patients must undergo a safety assessment as soon as possible, but no later than 30 days after the last dose of study treatment in Phase A, and an end of treatment (EoT) safety assessment no later than 30 days after the last dose of study treatment in Phase B. If patients are withdrawn from the study or have withdrawn consent, the last date of study treatment and reasons why treatment has been withdrawn early will be recorded in the eCRF.

Patients will have subsequent follow-up visits every 12 weeks until study end. At the study end, all patients will have an end of study follow-up visit to evaluate progression, survival status and safety.

Patients, who have discontinued study treatment for reasons other than progressive disease whilst in either Phase A or Phase B, will enter follow-up.

Primary In Situ Tumor Substudy
In order to evaluate the safety and effectiveness of bevacizumab-based therapy in patients with a primary in situ tumor an exploratory cohort of approximately 45 patients with a primary in situ colorectal tumor not requiring surgical intervention prior to initial chemotherapy will be enrolled into the study.

	Patients are required to meet all of the eligibility criteria for the main ASCENT study in addition to meeting the eligibility criteria defined for the primary in situ tumor cohort.
**NUMBER OF SUBJECTS**	Approximately 150 patients will be enrolled into the study, (approximately 105 resected primary tumor population patients and approximately 45 primary in situ patients). Recruitment to both patient groups will be competitive recruitment.
**TARGET POPULATION**	Patients with histologically confirmed, previously untreated mCRC eligible to commence treatment with bevacizumab in combination with oxaliplatin-based chemotherapy.

Resected primary tumor population
Inclusion Criteria

1. Signed informed consent obtained prior to any study specific procedures and willingness to comply with the study requirements (including biomarker sampling and tumor sampling for biomarkers).
2. Patients must be ≥ 18 years old.
3. Histologically confirmed, previously untreated metastatic colorectal cancer and not a candidate for curative resection.
4. WHO performance status of 0 -1.
5. Life expectancy of ≥ 3 months.
6. Eligible for XELOX, mFOLFOX6, FOLFIRI and bevacizumab treatment in accordance with local standards of care and PBS guidelines.

Exclusion Criteria

1. Previous chemotherapy for metastatic colorectal cancer.
2. Previous neoadjuvant or adjuvant chemotherapy completed within 6 months prior to commencement of study treatment.
3. Radiotherapy within 28 days prior to enrolment or from which patients have not yet recovered. Patients may be given palliative radiotherapy to peripheral sites (e.g. bone metastasis) within 28 days prior to enrolment but must have recovered from reversible acute side effects.
4. Patients with a history of non-colorectal malignancies are eligible if they have been disease-free for ≥ 5 years prior to study enrolment and are deemed by the physician to be at low risk for recurrence. However, patients with the following cancers are eligible if diagnosed and treated

within the previous 5 years: carcinoma in situ of the colon, melanoma in situ, basal cell and squamous cell carcinoma of the skin, and carcinoma in situ of the cervix.

5. Presence of active inflammatory bowel disease.
6. History of gastrointestinal perforations.
7. Symptomatic or bulky peritoneal disease.
8. Evidence of bleeding diathesis or significant coagulopathy (in absence of therapeutic anticoagulation). History of a significant bleeding event or considered to be at risk of such events.
9. Significant vascular disease (e.g. aortic aneurysm requiring surgical intervention).
10. Peripheral arterial thrombosis or other arterial thrombotic events within 6 months prior to commencement of study treatment.
11. Inadequately controlled hypertension (defined as values consistently > 150/100 mmHg despite use of at least three standard antihypertensive medications).
12. Prior history of hypertensive crisis or hypertensive encephalopathy.
13. Clinically significant (i.e. active) cardiovascular disease (e.g. NYHA Class II or greater congestive heart failure).
14. Concurrent major surgery unrelated to a primary in situ colorectal tumor and including invasive procedures defined as follows:
    a) Core biopsy or other minor procedure, excluding placement of a vascular access device, within 7 days prior to enrolment,
    b) Major surgical procedure, open biopsy, or significant traumatic injury within 28 days prior to enrolment,
    c) Planned major surgical procedures unrelated to a primary in situ colorectal tumor during the study.
15. Minor surgical procedures within 2 days prior to enrolment (including central venous access device placement for chemotherapy administration, tumor biopsies, needle aspirations, dental procedures).
16. Chronic active infection.
17. Investigational treatment within 28 days prior to enrolment.
18. Previous anti-angiogenic therapy (e.g. anti-VEGF or VEGF-R, tyrosine kinase inhibitor).
19. Previous anti-EGFR therapy (e.g. cetuximab or panitumumab).
20. Chronic daily treatment with oral corticosteroids (dose >

	10 mg/day methylprednisolone equivalent) excluding inhaled steroids.<br>21. Known hypersensitivity to any of the study drugs.<br>22. Pregnant or lactating females (a serum pregnancy test to be assessed within 7 days prior to study treatment, or within 14 days with a confirmatory urine pregnancy test within 7 days prior to study treatment).<br>23. Women of childbearing potential (defined as < 2 years after last menstruation and not surgically sterile) not using appropriate method of contraception and not willing to use an effective method of contraception during the study and for 6 months after the last dose of study medication. Oral or injectable contraceptive agents cannot be the sole method of contraception.<br>24. Male patients must be surgically sterile or agree to use a barrier method of contraception.<br>25. Systemic inflammatory disorder (eg rheumatoid arthritis).<br><br>**Patients with primary tumor in situ**<br><br>***<u>Inclusion Criteria</u>***<br>*Resected primary tumor population inclusion criteria apply in addition to the following criteria:*<br>1. Intact primary tumor of the colon or rectum not requiring surgical intervention prior to commencing chemotherapy.<br>2. Minimally or asymptomatic primary tumor (without obstruction, perforation or active bleeding requiring transfusion).<br><br>***<u>Exclusion criteria</u>***<br>*Resected primary tumor population exclusion criteria apply in addition to the following criteria:*<br>1. Prior endoscopic management of the current malignancy other than biopsy, including endoscopic stent placement, fulguration, or laser treatment.<br>2. Acute diverticulitis.<br>3. Presence of intra-abdominal abscess.<br>4. Active gastroduodenal ulcer(s).
**STUDY DURATION**	150 patients will be recruited into the study, or recruitment will cease after 24 months, whichever occurs first.
**END OF STUDY**	The end of the study is defined as 24 months after the date of the commencement of treatment for the last patient enrolled, or once all patients have died or have withdrawn from the study, whichever occurs first, but may be prematurely terminated by the sponsor. Hence the duration of the study will be approximately 48 months.

INVESTIGATIONAL PRODUCT(S) DOSE/ ROUTE/ REGIMEN

In this study, bevacizumab administered beyond 1st disease progression (Phase B) is considered to be the "investigational study drug". Bevacizumab administered as Phase A treatment is considered to be standard-of-care "non-investigational drug". XELOX, mFOLFOX6 and FOLFIRI are considered standard of care "non-investigational combination drug". Collectively, they will be known as the „study treatment".

In Phase A treatment, PBS-supplied bevacizumab will be administered at a dose of either 5.0 mg/kg or 7.5 mg/kg iv to coincide with XELOX (where bevacizumab will be administered every 3 weeks) or mFOLFOX6 (where bevacizumab will be administered every 2 weeks.)

In Phase B treatment, bevacizumab will be administered at a dose of 5.0 mg/kg iv on day 1 every 2 weeks in combination with FOLFIRI.

Bevacizumab treatment in Phase A and Phase B will continue until documented disease progression in each phase, unmanageable toxicity or patient withdrawal from the study.

For all bevacizumab medication administered prior to first progression, the approved regulatory (PBS) requirements will be applicable. During Phase B treatment, bevacizumab will be supplied by Roche Products Pty Limited.

All other study treatment will be supplied through the PBS scheme.

Study treatment phases

- *Phase A treatment*: Eligible patients will receive bevacizumab in combination with XELOX or mFOLFOX6 from the start of the study until 1st disease progression or the occurrence of an unmanageable toxicity or withdrawal from the study.
- *Phase B treatment*: Upon documented 1st disease progression, patients will continue to receive bevacizumab in combination with FOLFIRI until 2nd disease progression, or the occurrence of an unmanageable toxicity or withdrawal from the study.
- Phase B (bevacizumab plus FOLFIRI) must commence within 4 weeks of the date of documented 1st progression.

Study treatment administration

<u>XELOX + bevacizumab (Phase A treatment)</u>

Frequency: every 3 weeks

Drug dosages:

- Oxaliplatin: 130 mg/m^2 iv day 1

- Capecitabine: 1000 mg/ m² p.o twice daily days 1 to 14
- Bevacizumab: 7.5 mg/kg iv day 1
- *If oxaliplatin is discontinued, capecitabine and bevacizumab should be continued (maintenance therapy) until 1st disease progression or unmanageable toxicity*

<u>mFOLFOX6 + bevacizumab (alternative Phase A treatment)</u>

Frequency: every 2 weeks

Drug dosages:

- Oxaliplatin: 85 mg/m² iv day 1
- Leucovorin (LV): 400 mg/m² iv day 1 (*investigators may elect to use low-dose leucovorin i.e. either 20 mg/m² or 50 mg total dose*)
- Fluorouracil (5-FU): 400 mg/m² iv day 1
- Fluorouracil (5-FU): 2400 mg/m² continuous iv infusion over 46 hours day 1
- Bevacizumab: 5.0 mg/kg iv day 1
- *If oxaliplatin is discontinued, 5-FU/LV and bevacizumab should be continued (maintenance therapy) until 1st disease progression or unmanageable toxicity*
- *If oxaliplatin is discontinued, 5-FU/LV can be replaced with capecitabine and the 7.5 mg/kg once every 3 weeks bevacizumab schedule at the discretion of the investigator*

<u>FOLFIRI + bevacizumab (Phase B treatment)</u>

Frequency: every 2 weeks

Drug dosages:

- Irinotecan**:** 180 mg/m² iv day 1
- Leucovorin (LV): 400 mg/m² iv day 1 *(investigators may elect to use low-dose leucovorin i.e. either 20 mg/m² or 50 mg total dose)*
- Fluorouracil (5-FU): 400 mg/m² iv day 1
- Fluorouracil (5-FU): 2400 mg/m² continuous iv infusion over 46 hours day 1
- Bevacizumab: 5.0 mg/kg iv day 1
- *If irinotecan discontinuation is required, 5-FU/LV plus bevacizumab should be continued until 2nd disease progression or unmanageable toxicity.*
- *If irinotecan is discontinued, 5-FU/LV can be replaced with capecitabine and the 7.5 mg/kg once every 3 weeks bevacizumab schedule at the discretion of the investigator.*

	**Oxaliplatin reintroduction**<br>Reintroduction of oxaliplatin after discontinuation is not permitted in either Phase A or Phase B. Oxaliplatin based therapy can be re-initiated at investigator discretion after entry into the follow-up phase.<br><br>**5-FU/Capecitabine dosing**<br>In case of 5-FU or capecitabine dose reductions, the dose of fluoropyrimidine during re-introduction should not exceed the dose as last administered.<br><br>**Drug holidays**<br>– If deemed clinically appropriate by the investigator, study treatment may be temporarily suspended for up to four weeks for reasons other than toxicity and/or surgery during each of Phase A and Phase B (eight weeks holiday in total across both Phases A and B).<br>– If study treatment is suspended for more than the permitted four weeks for reasons other than toxicity and/or surgery in either treatment phase, patients will be withdrawn from treatment and enter follow-up.<br><br>**Bevacizumab monotherapy**<br>– If all chemotherapy is suspended for up to four weeks due to toxicity, bevacizumab alone may continue until chemotherapy is restarted or until the decision is made to permanently discontinue all chemotherapy.<br>– If all chemotherapy must be permanently discontinued due to toxicity, bevacizumab should be discontinued and the patient should enter follow up.
**COMPARATOR DRUG DOSE/ ROUTE/ REGIMEN**	There is no comparator drug in this study.
**TREATMENT MODIFICATION**	• Grade 4 non-hematological adverse events (AE) (except nausea and vomiting) will lead to discontinuation of study treatment and patients will enter follow up.<br>• The bevacizumab dose should not be reduced or modified with the exception of ≥ 10% weight change, where the treatment dosage should be adjusted

	accordingly.<br>• Missed bevacizumab doses will not be administered subsequently. The next bevacizumab dose should be administered at the next scheduled cycle of treatment.<br>• Dose modifications for the selected chemotherapy should be made according to local practice guidelines.

ASSESSMENTS OF EFFECTIVENESS:

Comparisons will be made between patients with baseline NLR $\leq$5 and those with baseline NLR >5 for the following parameters:

- PFS
  - *Measured from start of treatment to documentation of 1st disease progression or death from any cause, whichever occurs first.*
  - *PFS will be determined for all patients who have received a dose of study drug.*
- PFS in Phase B (PFS-B)
  - *Measured from start of Phase B treatment to documentation of 2nd disease progression or death from any cause, whichever occurs first.*
- DDC (PFS + PFS-B)
- Time to Failure of Strategy (TFS)
  - *Measured from start of treatment to documentation of 1st disease progression without entering Phase B, or 2nd progression having entered Phase B.*
- Overall survival (OS)
  - *Measured from start of treatment to death of any cause.*
- Survival beyond Progression (SBP)
  - *Measured from date of 1st disease progression to death of any cause.*
- Overall Survival in Phase B (OS-B)
  - *Measured from date of 1st treatment in Phase B to death of any cause.*
- Best overall response rate (ORR) in each Phase
  - *Defined as complete or partial response to therapy in either treatment phase as determined by the investigator.*
- Rate of curative liver resection
  - *Defined as the proportion of patients undergoing potentially curative liver resection.*

SAFETY

All clinical safety examinations will be performed as indicated in the Schedule of Assessments. Additional assessments may be performed as clinically indicated.
General physical examination, measurement of vital signs, laboratory safety assessments and recording of AEs/SAEs will be completed at all study visits.

Determination of relatedness to bevacizumab is at the discretion of the investigator.

SAEs caused by a protocol mandated intervention will be collected from informed consent and prior to initiation of any study medications.

AEs (including AEs of special interest) suspected to be related to bevacizumab occurring during the study will be reported from the first dose of bevacizumab and up to 6 months after the last bevacizumab dose.
SAEs (including AEs of special interest) suspected to be related to bevacizumab must be collected and reported regardless of the time elapsed from the last study drug administration, even if the study has been closed.

All AE/SAEs related to bevacizumab or otherwise, will be monitored until resolution or stabilization.

All AEs/SAEs unrelated to bevacizumab occurring during the study will be reported from consent and up to 28 days after last bevacizumab dose.

All concomitant medications including concomitant therapies of interest and antineoplastic therapy (agents with known antineoplastic properties), will be collected in the eCRF at each study visit. Concomitant therapies of interest include:

- anti-hypertensive medications
- anti-platelet drugs
- anticoagulation medications
- cholesterol-lowering medications
- anti-inflammatory drugs e.g. NSAIDS, COX-2s or corticosteroids (commenced whilst on study)
- growth factors

Safety assessments in line with local standard of care or those that are symptom-directed should be performed at the investigator"s discretion.

Clinical and laboratory AEs will be reported and graded according to the National Cancer Institute"s Cancer Toxicity Criteria for Adverse Events (NCI-CTCAE), version 4.0.

A negative serum pregnancy test (in women of childbearing potential) within 7 days prior to commencement of study treatment (or within 14 days with a confirmatory urine pregnancy test within 7 days prior to commencement of study treatment) is required for study entry.

- **QUALITY OF LIFE (QoL)**

Quality of Life assessments used in the study will include:

- EuroQol 5-D
- AQoL-8D
- FACT-C

Patients will be asked to complete each questionnaire at baseline, at each study visit during treatment phases and at the end of treatment follow-up visit. The completion of these questionnaires at each survival follow up visit is optional.

- **EXPLORATORY BIOMARKERS**

Consent for blood plasma and tumor tissue sample collection is mandatory for inclusion in the study. Results of all biomarker assays will be correlated with clinical outcomes.

Plasma biomarkers

These samples will be used for research purposes to identify dynamic biomarkers that are predictive of response to bevacizumab and chemotherapy treatment (in terms of dose, safety and tolerability) and will help to better understand the pathogenesis, course and outcome of colorectal cancer and related diseases. An initial pilot study will be undertaken using plasma samples from the first 50 patients to enroll into the study. Samples for all remaining patients will be collected and stored for future analysis.

A serial collection of plasma samples will be carried out. These samples will be collected at defined time points according to the Schedule of Assessments and appendices

A correlative analysis will be undertaken to evaluate the relationship between acute-phase plasma proteins, NLR and treatment outcomes as well as the influence of standard biochemical parameters on therapeutic outcomes.

The following will be assessed:

- the relationship between liver-derived acute phase proteins and therapeutic outcomes;
- the relationship between the neutrophil/lymphocyte (NLR) and platelet/lymphocyte (PLR) ratios and therapeutic outcomes;
- the influence of the modified Glasgow Prognostic Score (mGPS) on therapeutic outcomes;
- adjusted calcium, bilirubin, alkaline phosphatase, aspartate transaminase, alanine transaminase and γ-glutamyl transferase;
- GERCOR 2-stage prognostic model (baseline LDH and WHO performance status).

Tumor biomarkers

Archival tumor tissue samples will be collected in the form of formalin-fixed paraffin blocks (or parts of tumor blocks). Tissue can be from either the primary tumor or a metastatic site. A fresh biopsy is not required for enrolment into the study. If any additional tumor tissue becomes available as part of the patient‟s routine medical care during study ML25753, samples of this tissue are also to be collected.

Tumor tissue blocks will be used to set up a tissue microarray (TMA) for immunohistochemistry (IHC) analysis and potentially for the extraction of DNA and RNA. The following markers will be assessed:

- markers of IL-6-mediated inflammatory pathways
- markers of angiogenesis (VEGF, VEGFR1/R2, neuropilin-1, CD31)
- markers of tumor biology, tumor necrosis, vascularity, cell turnover, and expression of molecules in the VEGF families and their signaling pathway molecules
- other possible markers considered relevant to efficacy or safety outcomes if indicated at the time of analysis.

Sample size justification

It is anticipated that the hazard ratio for NLR in the primary model will be larger than the hazard ratio (1.6) observed by Chua *et al.* in their multivariate analysis. Assuming the true incidence of NLR > 5 at baseline is 30%, the median progression free survival is 10.5 months, and all patients are followed for 24 months, 150 patients provides approximately 80% power to detect a Hazard Ratio of 1.7.

Primary parameter

- The Neutrophil/Lymphocyte ratio at the start of treatment will be dichotomized between ≤ 5 and > 5 and tested in a Cox regression model for an association with Progression Free Survival, in a model adjusted for the default covariates.

The following covariates will be used by default:

- WHO Performance Status (0 vs. 1)
- Metastatic disease in the liver (Yes/No)
- Number of different sites of metastatic disease (≤ 3 vs. > 3)
- Presence of metastatic disease in the liver with no other sites involved (Yes/No)

The justification for this choice of covariates is that general health (WHO Performance Status) and disease burden are known to be associated with progression free survival independently of the inflammatory processes being modeled by the NLR. It was decided not to adjust for hypoalbuminemia, or anemia, both of which are associated with PFS, because it is believed they may be modeling

the same inflammatory process as NLR. Age was not included because it was considered likely to be correlated with, and less predictive of outcome, than Performance Status.

Other measures of tumor burden, extent of involvement of metastatic sites, and laboratory analytes will be investigated in exploratory analyses.

Secondary parameters

- Incidence rates of safety and tolerability outcomes in patients on bevacizumab including:
  - All adverse events
  - All CTCAE Grade 3-5 adverse events
  - All adverse events suspected to be related to bevacizumab
  - Adverse events of special interest, including:
    - GI perforation - any Grade
    - GI Fistula / Abscess – any Grade
    - Bleeding – Grade 3 or above
      - Bleeding (CNS) - Grade 3 or above
      - Bleeding (Non-CNS) - Grade 3 or above
    - Wound healing complications/dehiscence – Grade 3 or above
    - ATE – any Grade
    - VTE – Grade 3 or above
    - Hypertension – Grade 3 or above
    - RPLS – any Grade
    - Febrile neutropenia – any Grade
    - Proteinuria – Grade 4 or above
    - CHF – Grade 3 or above
  - All serious adverse events.
- Quality of Life (QoL) outcomes.
- Health outcomes in patients on bevacizumab as measured by:
  - Progression-free survival for all patients (PFS)
  - Progression-free survival in Phase B (PFS-B)
  - Duration of Disease Control (DDC)
  - Time to Failure of Strategy (TFS)
  - Survival Beyond Progression (SBP)
  - Overall survival from start of treatment (all patients who receive at least one dose of bevacizumab) (OS)
  - Overall survival for patients receiving bevacizumab beyond progression (all patients receiving at least one dose of bevacizumab in Phase B) (OS-B)
  - Best overall response rate overall and in each treatment Phase (ORR)
  - Rate of curative liver resection.

- In patients with a primary in situ tumor:
  - The incidence of serious adverse events, including, but not limited to, those causing major morbidity in patients with an unresected minimally symptomatic or asymptomatic primary colorectal tumor. Specifically, bleeding, perforation, bowel obstruction, or fistula formation requiring surgery, adverse events related to the primary tumor requiring hospitalization or a major non-surgical intervention are events.

Analysis populations

The Full Analysis Set will include all patients who receive at least one dose of bevacizumab.

The "Primary In Situ population" will include all patients in the Full Analysis Set with a primary in situ tumor.

The "Resected Primary Tumor population" will include all patients in the Full Analysis Set without a primary in situ tumor.

Statistical analysis

All analyses will be conducted on the Full Analysis Set unless otherwise stated. There are no pre-defined hypotheses regarding the frequency or severity of adverse events or treatment effectiveness. All testing of statistical hypotheses will be conducted at two-sided alpha of 0.05.

PART I: STUDY DESIGN AND CONDUCT

1. BACKGROUND AND RATIONALE

1.1 Colorectal Cancer

Colorectal cancer (CRC) is the second most commonly diagnosed cancer in Australia for both sexes combined, with a projected annual incidence of more than 13000 cases and 4100 deaths in 2010. Approximately 25% of patients present with metastatic CRC (mCRC) at initial diagnosis and almost 50% of patients with CRC will develop future metastases [1]. For the majority of patients diagnosed with mCRC palliative fluoropyrimidine-based chemotherapy is the most appropriate treatment option in order to prolong survival and improve quality of life (QoL). Although the prognosis for patients with mCRC still remains poor, treatment outcomes have improved significantly in the last decade as a result of the introduction of new systemic treatments and expanded use of hepatic metastectomy with median survival now well in excess of two years [2].

The backbone of first- and second-line palliative chemotherapy for mCRC consists of a fluoropyrimidine (infusional 5-FU or oral capecitabine) in various combinations and schedules. Combination chemotherapy with fluoropyrimidine /oxaliplatin (FOLFOX or XELOX) or 5-FU/LV/irinotecan (FOLFIRI) provides higher response rates, longer progression-free survival (PFS) and better overall survival (OS) than fluoropyrimidine alone. Both FOLFOX/XELOX and FOLFIRI have similar efficacy regardless of the sequence used but have differential toxicity profiles [3]. Survival is correlated significantly with the percentage of patients receiving all active chemotherapeutic agents, emphasizing the importance of exposure to all active drugs during treatment [4].

1.2 Bevacizumab

Bevacizumab is a humanised monoclonal antibody targeting VEGF or VEGF-A which is a ligand with a central role in signalling pathways controlling tumor angiogenesis [5]. The mode of action of bevacizumab can be summarised as follows:

- Prevents the formation of new blood vessels, thereby inhibits the growth of existing tumors and prevents metastases from developing blood supply.
- Normalizes existing tumor blood vessels. The subsequent effects include reduction of the tortuousness of tumor blood vessels and normalisation of vessel permeability. The latter effect is important because tumors usually exhibit high interstitial pressure which can prevent chemotherapeutic agents penetrating tumors and accessing tumor cells, where they exert their effects. Normalization of permeability, and therefore intratumoral pressure gradients, thus, promotes chemotherapy access [6].
- Ultimately, produces blood vessel breakdown, probably through inhibition of the anti-apoptotic effects of VEGF on immature endothelial cells.

VEGF also has activities beyond angiogenesis, affecting immune function via inhibition of dendritic cell maturation, formation of lymph vessels and lymphatic metastasis. VEGF is continually expressed throughout the lifecycle of many solid tumors meaning that

continuous VEGF suppression is likely to be the key to achieving and maintaining optimal tumor control [7].

1.2.1 Bevacizumab in mCRC

The use of bevacizumab in combination with fluoropyrimidine-containing chemotherapy is a well established first-line and second-line treatment for patients with mCRC [8-14]. Although bevacizumab is well established in the mCRC treatment algorithm a number of data gaps remain to be addressed, notably, the need for reproducible, validated, inexpensive and easy to administer biomarkers to aid prognostication and patient selection. The optimal duration of treatment and the role of bevacizumab in certain patient subgroups considered at particular risk of bevacizumab-mediated toxicity also require further investigation.

1.2.2 Bevacizumab beyond progression (BBP)

A wealth of preclinical models support the notion that VEGF is continually expressed throughout the lifecycle of the tumor and that sensitivity to anti-VEGF antibodies remains even after disease progression [7]. The continuation of bevacizumab after disease progression on bevacizumab-based first-line treatment is common practice in countries such as the US [15].

The use of BBP has been addressed in a recently completed, randomized phase III trial (ML18147)[17]. This phase III study evaluated bevacizumab continued with second-line chemotherapy in mCRC patients who received initial bevacizumab plus first-line chemotherapy. The study met its primary endpoint of a significant increase in OS. In the study, the relative risk of death was reduced by 19 percent for people who continued with bevacizumab plus second-line chemotherapy compared with those who received chemotherapy alone (HR = 0.81, p = 0.0062). Patients who continued with bevacizumab plus second-line chemotherapy also experienced a significant improvement in PFS: the risk of their cancer progressing was reduced by 32 percent (HR = 0.68, p<0.0001). Adverse events in ML18147 were consistent with those seen in previous pivotal trials of bevacizumab across tumor types. This study, however, was not open in Australia and did not collect data on QoL.

The results of a second randomized trial evaluating the continuation of bevacizumab beyond progression in mCRC patients who received bevacizumab as part of first-line treatment have been reported [18]. The study met its primary endpoint in significantly improving PFS in those patients who received bevacizumab plus chemotherapy versus patients receiving chemotherapy alone (HR = 0.65, p=0.0062). These results are in line with those reported in ML18147 and support the continuation of bevacizumab beyond progression in mCRC.

1.2.3 Bevacizumab in patients with a primary in situ tumor

There has been a recent shift in clinical practice away from preemptive resection of the primary tumor in patients with synchronous unresectable disease. In the presence of a primary in situ tumor, concerns remain about combining cytotoxic chemotherapy with bevacizumab in patients with a primary in situ tumor because of risk of gastrointestinal (GI) perforation. Bevacizumab has been associated with a 1-2% incidence of GI

perforation in prospective clinical trials and observational studies [12-14, 16, 19-21]. Potential risk factors for GI perforation include [22-25]:

- Primary in situ tumor
- Acute diverticulitis
- Intra-abdominal abscess
- Gastrointestinal obstruction
- Abdominal carcinomatosis
- Previous abdominal or pelvic radiation
- Pre-existing palliative endoluminal stents

There is a paucity of data concerning the use of bevacizumab in the setting of a primary in situ tumor [26, 27] and the presence of an unresected primary tumor is a common reason to withhold bevacizumab in Australian clinical practice [28]. The recent NSABP C-10 trial contributed important safety information regarding bevacizumab use in this setting [26]. With a primary end point of major morbidity (defined as obstruction, perforation, bleeding or death), this single-arm phase II trial enrolled patients with an asymptomatic, unresected colonic primary who received mFOLFOX6 plus bevacizumab in a Simon two-stage design, with stage I powered to rule out a 40% rate of major morbidity. In this cohort, 10/86 (~12%) patients required surgery directed at the primary tumor (obstruction, n = 8; perforation, n = 1; pain, n = 1) and there was a 14% major morbidity rate, suggesting that primary in situ tumors and bevacizumab use are not incompatible. Despite the NSABP C-10 trial, there are still data gaps present when considering bevacizumab in the setting of a primary in situ tumor. NSABP C-10 was confined to patients with an in situ and asymptomatic colonic primary and excluded those with an in situ rectal primary. Patients may also present in clinical practice with a minimally symptomatic primary colorectal lesion. As a result there is a need for further data in patients presenting with mCRC who have an in situ and minimally symptomatic colorectal primary to guide clinical decision making.

1.3 Inflammation as a potential biomarker for bevacizumab in mCRC

Biomarkers have an increasingly important role in both cancer research and clinical practice. Biomarkers can be used to assess prognosis and to predict how individual patients will respond to specific treatments [29, 30]. Despite concerted international research efforts, no validated and easy to administer biomarker yet exists to prognosticate on or predict treatment outcomes for patients treated with bevacizumab. A broad range of blood- and tumor tissue-based markers have been explored during the development phase of bevacizumab (preclinical > 10 000; clinical > 100) with most of the existing data focused on VEGF pathway markers e.g. tumor VEGF expression [31] or oncogene mutations such as K-Ras [32, 33]. Relatively little attention has been paid to the role of biomarkers associated with the tumor microenvironment and host factors such as the inflammatory response. Both the tumor microenvironment and the inflammatory response are considered key aspects of cancer biology and tumorigenesis [34] and are important regulators of angiogenesis.

Tumor development and growth occur as a result of interactions between the tumor, host derived stromal tissues including blood vessels and host immune/inflammatory cells. It has been increasingly recognized that, in addition to tumor stage and proliferative activity, disease progression is dependent on a complex interaction between the tumor and the host inflammatory response (see Figure 1). Inflammation can affect every aspect of tumor development and progression as well as the response to therapy [35, 36]. In the tumor microenvironment, inflammation contributes to proliferation and survival of malignant cells, angiogenesis, metastasis, subversion of adaptive immunity, reduced response to hormones and chemotherapeutic agents. It has been suggested that cancer-related inflammation represents the 7th hallmark of cancer [37]. Infiltration of small tumors by inflammatory cells that produce proangiogenic ligands makes a contribution to the angiogenic switch that drives tumor growth. Tumor development and progression induced by an inflammatory response is thought to be mediated by an interaction between proinflammatory cytokines and pathways including NF-κB and STAT3 [38].

VEGF is overexpressed in cancer as a result of hypoxia, oncogenic signaling, or inflammation [6]. During tumor development, oncogenic mutations and inflammation drive the excessive production of angiogenic growth factors. This proangiogenic state results in hyperactive vessel growth and abnormal vascular changes leading to impaired tumor perfusion and renders the tumor more hypoxic and acidic. This creates a continuous, self-perpetuating cycle of abnormal angiogenesis resulting in compromised anti-tumor defense, augmented tumor tissue swelling, resistance to treatment and accelerated tumor invasion and dissemination.

Despite the benefit provided by bevacizumab-based regimens for patients with metastatic colorectal cancer, clinical resistance usually develops. Extensive preclinical work has suggested that alternate proangiogenic factors may modulate sensitivity to anti-VEGF therapy and allow regrowth of tumor-associated vasculature [39, 40]. Additional studies have implicated infiltrating inflammatory monocytic cells in the angiogenic switch, recruited by cytokines derived from tumor or tumor-associated stroma [41]. A recent preclinical study found that a subset of murine transplant tumors growing in mice showed no responsiveness to an anti-mouse VEGF monoclonal antibody that mimicked bevacizumab [41]. The non-responsive tumors, which had not previously been treated with chemotherapy, were characterized by a pre-existing infiltration of inflammatory mediators, principally $CD11b^{+}Gr1^{+}$ myeloid cells (defined as coexpressing the macrophage marker CD11b and the neutrophil marker Gr1), which were shown to express a number of pro-angiogenic factors. By contrast, the responsive tumor types had comparatively low levels of such inflammatory mediators. Changes in myeloid cell-associated proinflammatory cytokines also appear to be associated with clinical resistance to bevacizumab-based treatment [42]. Plasma levels of IL-18, a pro-inflammatory cytokine expressed by macrophages and monocytes which has been shown to stimulate migration of endothelial cells and induce angiogenesis *in vivo*, were shown to rise significantly along with other angiogenic cytokines and myeloid recruitment factors at progression after first-line treatment with bevacizumab plus FOLFIRI [42]. To date no studies have evaluated the correlation between blood-based inflammatory markers such as NLR and clinical resistance to bevacizumab.

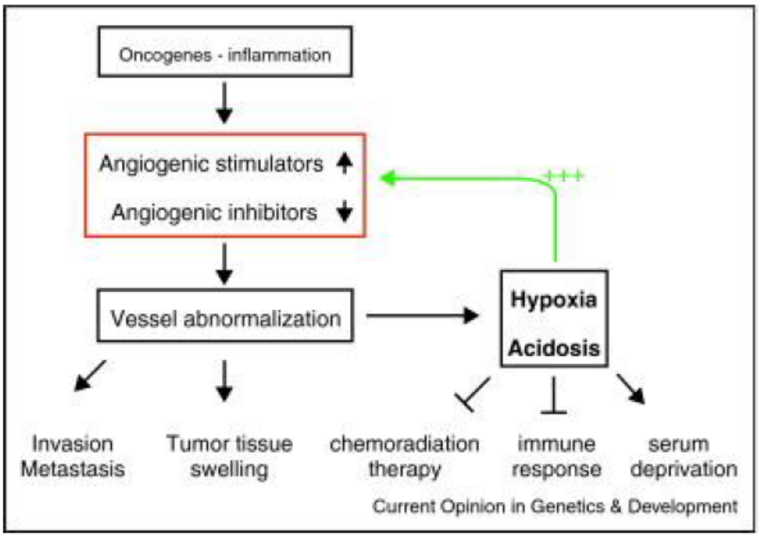

Figure 1: Links between cancer biology, angiogenesis and inflammation [6]

1.3.1 Inflammation-based biomarkers

Multiple systemic responses occur in response to inflammation caused by infection, tissue injury, immunological disorders or cancer [43]. These responses involve alterations in neuroendocrine metabolism (including the endocrine hormones), hematopoietic changes (including the relative numbers of circulating white cells and platelets), changes in protein and energy metabolism (including loss of muscle protein) and changes in acute-phase proteins (including C-reactive protein and albumin). The liver is a key player in the initiation and maintenance of the systemic inflammatory response. Hepatocytes are stimulated to synthesize, and release into the systemic circulation, a variety of acute-phase proteins that initiate, sustain or curtail the systemic inflammatory response.

Clinically, the most common reported measures of the systemic inflammatory response in cancer patients are biochemical or hematological markers (see Table 1). These measures include an elevated C-reactive protein (CRP) concentration or increased white cell, neutrophil and platelet counts. Hypoalbuminemia is also recognized to be part of the systemic inflammatory response. The neutrophil:lymphocyte ratio (NLR) combines circulating neutrophil and lymphocyte counts and the platelet:lymphocyte ratio (PLR) combines circulating platelet and lymphocyte counts. Combinations of biochemical factors have been used to derive simple inflammation-based prognostic scores. The Glasgow Prognostic Score (GPS) combines circulating CRP and albumin concentrations.

Scoring system	*Score*
**Neutrophil:lymphocyte ratio**	
**Neutrophil count:lymphocyte count < 5:1**	*0*
**Neutrophil count:lymphocyte count ≥ 5:1**	*1*

**Platelet:lymphocyte ratio**	
**Platelet count:lymphocyte count < 150:1**	*0*
**Platelet count:lymphocyte count 150 – 300:1**	*1*
**Platelet count:lymphocyte count > 300:1**	*2*
**The Glasgow Prognostic Score**	
**C-reactive protein ≤ 10 mg/l and albumin ≥ 35 g/l**	*0*
**C-reactive protein ≤ 10 mg/l and albumin < 35 g/l**	*0*
**C-reactive protein > 10 mg/l**	*1*
**C-reactive protein > 10 mg/l and albumin < 35 g/l**	*2*

Table 1: Systemic inflammation-based scoring systems

1.3.2 Neutrophil:lymphocyte ratio and mCRC treatment outcomes

In patients with liver-only colorectal metastases receiving neoadjuvant chemotherapy prior to hepatic metastectomy, an elevated NLR (> 5) predicted diminished survival [44]. In addition, those patients in whom NLR normalized after chemotherapy had significantly improved 1-, 3- and 5-year survival which was similar to patients with NLR ≤ 5 at baseline [44]. In a recent study by Chua *et al* [45], in patients with unresectable mCRC, an elevated pre-treatment NLR was found in ~30% of patients [45]. In this patient cohort, who underwent first-line combination chemotherapy, NLR was found to be an independent predictor of clinical benefit, progression and survival [45]. The NLR was statistically significantly associated with OS (P < 0.0001). Patients with NLR ≤ 5 had median OS of 19.1 months (95% CI 15.3–22.8) compared with patients with NLR > 5 (median OS 11.3 months; 95%CI 8.3–14.3). In addition, normalization of the NLR after one cycle of chemotherapy was associated with improved PFS [45]. These data support the use of NLR as a marker of systemic inflammatory response and as an independent predictor of outcome in patients receiving chemotherapy for mCRC.

Given the established link between systemic inflammation and tumor angiogenesis the potentially valuable role of inflammatory markers as predictive or prognostic tools in the setting of bevacizumab is of interest. The use of blood-based markers such as NLR as prognostic/predictive biomarkers in patients receiving bevacizumab-based chemotherapy has not yet been evaluated.

1.4 Rationale for the Study

No validated or reproducible biomarker yet exists to assist clinicians to prognosticate on the likely treatment outcomes for patients with mCRC treated with bevacizumab-containing regimens. Relatively little attention has been paid to the role of host/tumor microenvironment factors such as the inflammatory response and anti-angiogenic therapy. Chronic inflammation is thought to play an important role in cancer development, angiogenesis and disease progression and pathways mediating inflammation and tumor angiogenesis are tightly interlinked. Preliminary data suggest that the host inflammatory response, measured by differential white cell counts such as the neutrophil/lymphocyte ratio (NLR) may predict outcomes in patients with mCRC treated with first-line combination chemotherapy. This simple and readily available method of assessing a patient"s inflammatory status may also provide useful prognostic

information when combination chemotherapy is combined with bevacizumab. The influence of the host inflammatory response, as measured by NLR, has not yet been studied in the setting of bevacizumab and may represent a clinically useful prognostic marker.

The use of BBP has been addressed in a recently completed, randomized phase III trial (ML18147). This study however was not open in Australia and did not collect data on QoL. Furthermore, patients participating in randomized controlled trials often represent a subset of the general patient population and strict eligibility criteria in relation to disease and patient characteristics may influence the "generalizability" of both safety and efficacy results from clinical trials to the broader patient population. A secondary objective of ML25753 is to provide clinically relevant information regarding the safety, effectiveness and QoL of BBP in combination with standard chemotherapy regimens in a more generalized, community-based population of mCRC patients in Australia.

An increasing proportion of patients with mCRC at first presentation are treated with systemic chemobiologic therapy without pre-emptive resection of the primary tumor. Limited data currently exists to guide treatment decisions in this setting and uncertainty exists around the risk/benefit of bevacizumab-based treatment in patients with a primary in situ tumor. Although the recent NSABP-C10 study has provided useful data in the setting of an asymptomatic colonic primary tumor, similar studies have not yet been undertaken in patients with a minimally symptomatic primary or those with an in situ rectal primary lesion. It is therefore necessary to further study the safety and effectiveness of bevacizumab in the setting of an in situ primary rectal lesion. The ML25753 study will evaluate treatment outcomes of approximately 45 patients presenting with mCRC with a minimally symptomatic or asymptomatic primary colon or rectal tumor.

2. OBJECTIVES

2.1 Primary Objective

The primary objective of the study is to assess the prognostic value of the host inflammatory response as assessed by the Neutrophil:Lymphocyte Ratio (NLR ≤ 5 vs. > 5) on Progression Free Survival.

2.2 Secondary Objectives

To further characterize the safety profile of study treatment and evaluate its efficacy following treatment initiation, initial response and when continued after progression.

Secondary objectives consist of the following:

- To assess effectiveness outcomes as measured by (see Figure 2):
  - Progression-free survival until 1st progression (PFS)
  - Progression-free survival during Phase B (PFS-B)
  - Time to failure of strategy (TFS)
  - Duration of disease control (DDC)
  - Overall survival from the start of treatment (OS)

- Survival beyond 1st progression (SBP)
- OS during Phase B (OS-B)
- Best overall response rate as assessed by the investigator in each treatment phase and overall
- Rate of liver resection

Figure 2 Secondary Efficacy Objectives

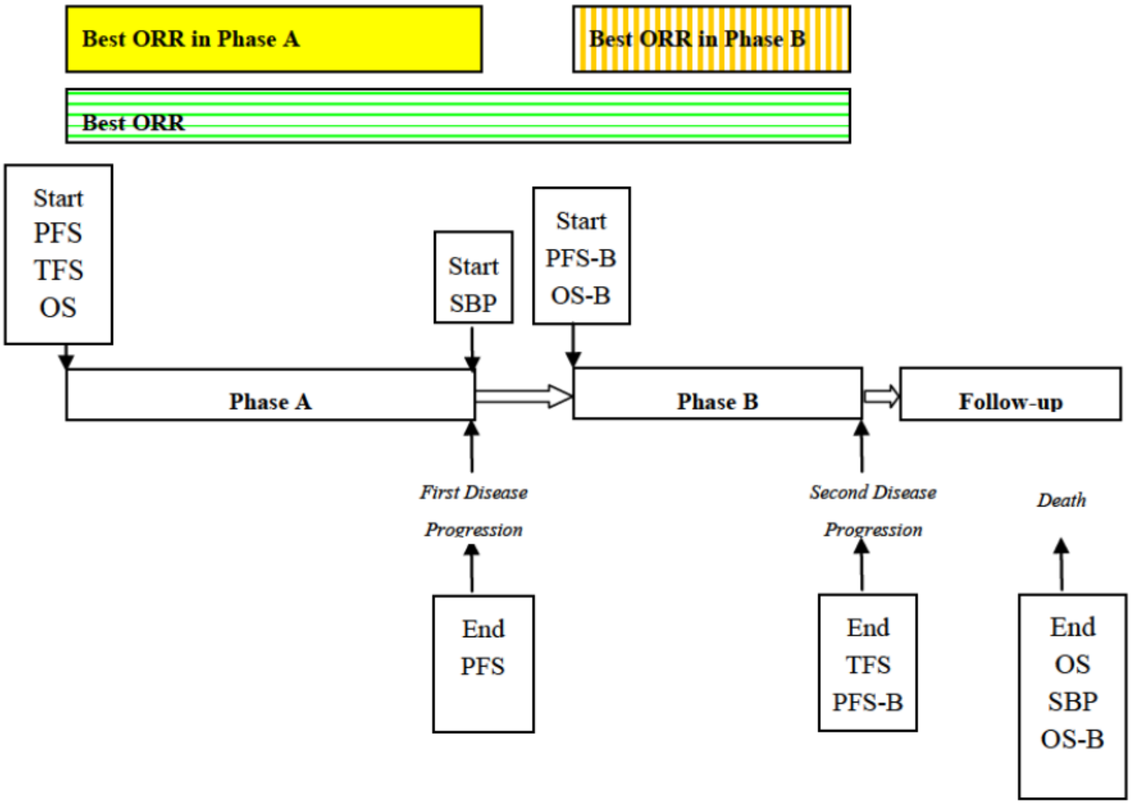

- To assess the overall incidence of adverse events, including (see Figure 3):
  - Relatedness to bevacizumab
  - Severity (CTCAE Grade 3-5)
  - Seriousness
  - Adverse events of special interest (see Section 7.2.2)

Figure 3 Secondary Safety Objectives

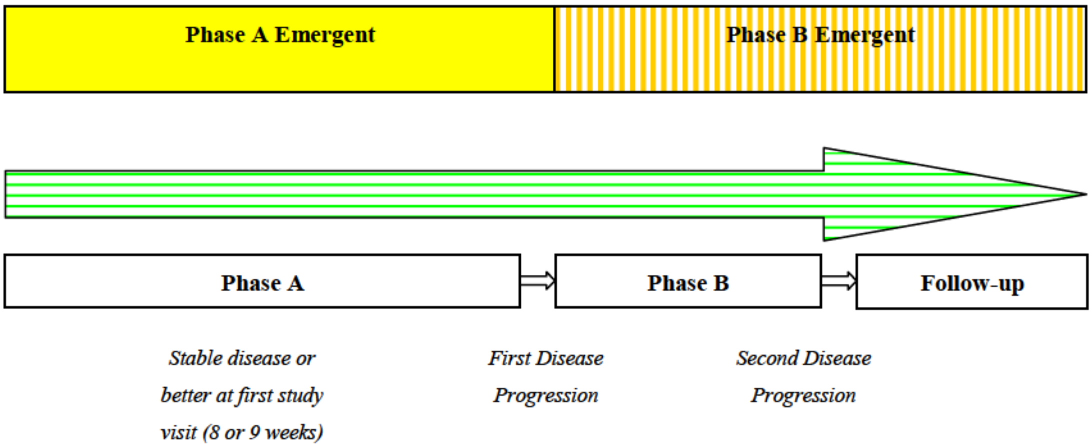

Includes all patients who received at least one dose of bevacizumab during treatment phase

Includes all patients who received at least one dose of bevacizumab in Phase A

Includes all patients who received at least one dose of bevacizumab in Phase B within 4 weeks of 1st progression

- To validate the NLR as a predictor of OS in patients treated with bevacizumab
- To assess the association between post-baseline changes in NLR and PFS and OS
- In the subgroup of patients with a primary in situ tumor, to assess the incidence of serious adverse events related to the primary in situ tumor. These include, but are not limited to, the following:
  - Colonic bleeding requiring surgical intervention
  - Perforation (contained or free) requiring surgery
  - Bowel obstruction requiring surgery
  - Fistula formation requiring surgery
  - Any events related to the primary in situ tumor resulting in patient death
  - Colonic obstruction not requiring surgery
    - a) Requiring hospitalization for medical management
    - b) Requiring stent placement, laser treatment or fulguration
  - Gastrointestinal (GI) bleeding requiring transfusion
  - Fistula formation not requiring surgery
    - a) Self-draining entercutaneous fistula
    - b) Intra-abdominal abscess requiring percutaneous drainage
  - Other events related to the primary tumor requiring hospitalization, but not surgery

- To assess patient reported Quality of Life (QoL)

2.3 Exploratory Objectives

Consent for blood plasma sample and tumor tissue collection is mandatory for inclusion in the study. The objective of biomarker profiling is to enable development of treatments specifically targeted for optimal patient benefit (personalized healthcare). Plasma and tumor samples for biomarker analysis will be stored centrally. For details refer to Sections 5.4.2, 5.4.3 and 18.1. The plasma and tumor specimens collected will be used to:

- study the role of systemic inflammation on treatment efficacy and/or adverse reactions related to chemotherapy regimens and bevacizumab treatment
- study the association of plasma biomarkers with efficacy and/or adverse reactions related to chemotherapy regimens and bevacizumab treatments
- study early, intermediate and late escape biomarkers and mechanisms related to bevacizumab and chemotherapy regimen
- increase knowledge of understanding of disease biology
- develop biomarkers or diagnostic assays and to establish the performance characteristics of these assays.

Plasma biomarkers

These samples will be used for research purposes to identify dynamic biomarkers that are predictive of response to bevacizumab and chemotherapy treatment (in terms of dose, safety and tolerability) and will help to better understand the pathogenesis, course and outcome of colorectal cancer and related diseases. An initial pilot study will be undertaken using plasma samples from the first 50 patients to enroll in the study. Samples for all remaining patients will be collected and stored for future analysis.A serial collection of plasma samples will be carried out. These samples will be collected at defined time points according to the Schedule of Assessments and appendices

A correlative analysis will be undertaken to evaluate the relationship between acute-phase plasma proteins, NLR and treatment outcomes (see Section 18.1).

Exploratory objectives include the following:

- To further characterize the relationship between blood-based markers of systemic inflammation and therapeutic outcomes in patients with mCRC treated with bevacizumab combined with standard fluoropyrimidine-based chemotherapy, including baseline and ongoing changes in:
    - the relationship between liver-derived acute phase proteins and therapeutic outcomes;
    - the relationship between the neutrophil/lymphocyte (NLR) and platelet/lymphocyte (PLR) ratios and therapeutic outcomes;

- the influence of the modified Glasgow Prognostic Score (mGPS) on therapeutic outcomes.

- To further characterize the influence of standard biochemical parameters on therapeutic outcomes, including baseline and ongoing changes in:

  - adjusted calcium, bilirubin, alkaline phosphatase, aspartate transaminase, alanine transaminase and γ-glutamyl transferase;
  - GERCOR 2-stage prognostic model (baseline LDH and WHO performance status).

Tumor biomarkers
Archival tumor tissue samples will be collected in the form of formalin-fixed paraffin blocks (or parts of tumor blocks). A fresh biopsy is not required for enrolment into the study. If any additional tumor tissue becomes available as part of the patient"s routine medical care during study ML25753, samples of this tissue are also to be collected.

Tumor tissue blocks will be used to set up a tissue microarray (TMA) for immunohistochemistry (IHC) analysis and potentially for the extraction of DNA and RNA. The following markers will be assessed:

- markers of IL-6-mediated inflammatory pathways
- markers of angiogenesis (VEGF, VEGFR1/R2, neuropilin-1, CD31)
- markers of tumor biology, tumor necrosis, vascularity, cell turnover, and expression of molecules in the VEGF families and their signaling pathway molecules
- other possible markers considered relevant to efficacy or safety outcomes if indicated at the time of analysis.

3. STUDY DESIGN

3.1 Overview of Study Design

This is an open-label, prospective, single arm, phase IV, Australian multi-center study evaluating the relationship between the host inflammatory response as measured by NLR and treatment outcomes in patients with previously untreated mCRC who will receive bevacizumab-based first- and second-line treatment. The trial design is illustrated in Figure 4.

The trial consists of two phases of treatment:

- *Phase A treatment*: XELOX or mFOLFOX6 plus bevacizumab administered from study start until 1st disease progression;
- *Phase B treatment*: FOLFIRI plus bevacizumab administered from 1st disease progression until 2nd disease progression.

Bevacizumab infusions will be administered on either a three-weekly basis in combination with XELOX or a two-weekly basis in combination with mFOLFOX6 throughout Phase A treatment until 1st disease progression or occurrence of unmanageable toxicity. Upon documented disease progression, Phase A treatment will be discontinued and bevacizumab will be continued in combination with FOLFIRI (Phase B treatment) until 2nd disease progression or unmanageable toxicity. Upon 2nd disease progression, all study treatment will be discontinued and patients will enter follow-up.
Phase B treatment (bevacizumab plus FOLFIRI) must commence within 4 weeks of the date of documented 1st disease progression.

Patients will attend study specific visits every 8 or 9 weeks (to coincide with chemotherapy regimen) throughout Phase A and B. All patients must undergo a safety assessment as soon as possible, but no later than 30 days after the last dose of study treatment in Phase A, and an end of treatment (EoT) safety assessment no later than 30 days after the last dose of study treatment in Phase B. If patients are withdrawn from the study or have withdrawn consent, the last date of study treatment and reasons why treatment has been withdrawn early will be recorded in the eCRF.

Patients will have subsequent follow-up visits every 12 weeks until study end. At the study end, all patients will have an end of study follow-up visit to assess disease progression, survival status and safety.

Patients who have discontinued study treatment for reasons other than progressive disease whilst in either Phase A or Phase B will enter follow-up.

3.1.1 Primary In Situ Subgroup

In order to evaluate the safety and effectiveness of bevacizumab-based therapy in patients with a primary in situ tumor an exploratory cohort of approximately 45 patients with an unresected primary colorectal tumor not requiring surgical intervention prior to initial chemotherapy will be enrolled into the study.

Patients are required to meet all of the eligibility criteria for the main ASCENT study in addition to meeting the eligibility criteria defined for the primary in situ tumor cohort.

Figure 4 Overview of study design

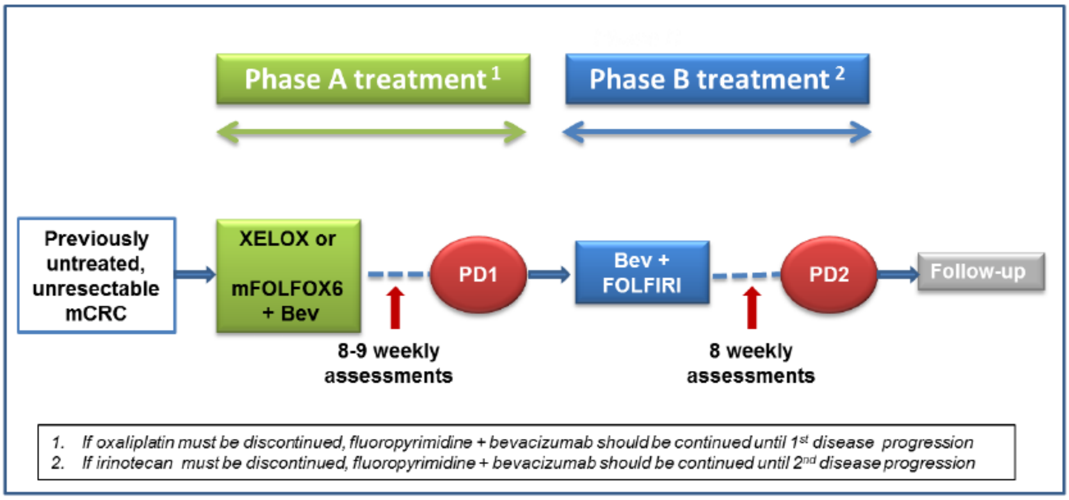

The definition of disease progression is provided in Section 5.3.6.

3.1.2 Rationale for Study Design

The ML25753 study is designed as a single-arm, open-label trial adding bevacizumab to standard first-line and second-line combination chemotherapy regimens in order to:

- Study the relationship between the host inflammatory response, as determined by the NLR, and treatment outcomes;
- Explore the safety and effectiveness of the BBP paradigm in a generalized population of patients with mCRC including patients with a primary in situ tumor.

The study design will allow the serial measurement of NLR throughout first-line and second-line treatment using routine pre-chemotherapy differential blood counts. The results will indicate the potential value of NLR as a biomarker of treatment outcome or resistance to bevacizumab treatment. Plasma will be collected at baseline, during treatment and upon disease progression to study the relationship between acute-phase inflammatory proteins and treatment outcomes and to further investigate the biological underpinnings of the host inflammatory response. Baseline samples of tumor tissue will be collected to evaluate the role of tumor-based markers on treatment outcomes and to determine the relationship between host inflammatory response and tumor-based markers.

3.1.3 Rationale for Dose Selection

Doses and regimens of XELOX, mFOLFOX6 and FOLFIRI administered throughout Phase A or Phase B are as per local treatment recommendation and PBS requirements.

The recommended dose of bevacizumab for the treatment of mCRC in the first-line setting is 5.0 mg/kg given once every 2 weeks or 7.5 mg/kg given once every 3 weeks as an intravenous infusion. The dose and regimen of bevacizumab administered throughout Phase A are as per local recommendations and PBS requirements.

The dose of bevacizumab administered throughout Phase B (5.0 mg/kg once every 2 weeks) is in line with the dose used in a Phase III study (ML18147) and in two observational studies (BRiTE and ARIES) evaluating the safety and efficacy of bevacizumab continued beyond progression.

3.1.4 Study Duration

150 patients will be recruited into the study, or recruitment will cease after 24 months, whichever occurs first. The end of the study is defined as 24 months after the date of the commencement of treatment for the last patient enrolled. Hence the duration of the study will be approximately 48 months.

3.1.5 End of Study

The study will formally end 24 months after the date of the commencement of treatment for the last patient enrolled or once all patients have died or have withdrawn from the study, whichever occurs first, but may be prematurely terminated by the sponsor.

3.2 Number of Subjects

Approximately 150 patients will be enrolled into the study (approximately 105 resected primary tumor population patients and approximately 45 primary in situ tumor patients) or recruitment will cease after 24 months, whichever occurs first. Recruitment to both patient groups will be competitive recruitment.

3.3 Centers

16 centers are participating in the study across Australia.

4. STUDY POPULATION

4.1 Overview

The target population for this study includes male and female adult patients with histologically confirmed mCRC eligible to commence first-line treatment with bevacizumab in combination with XELOX or mFOLFOX6.

4.2 Inclusion Criteria

Resected primary tumor population

A patient may be included in the resected primary tumor population if the answer to all of the following statements is "yes".

1. Signed informed consent obtained prior to any study specific procedures and willingness to comply with the study requirements (including plasma and tumor sampling for biomarkers).
2. Patients must be ≥ 18 years old.
3. Histologically confirmed, previously untreated metastatic colorectal cancer and not a candidate for curative resection.
4. WHO performance status of 0 - 1.
5. Life expectancy of ≥ 3 months

6. Eligible for XELOX, mFOLFOX6, FOLFIRI and bevacizumab treatment in accordance with local standards of care and PBS guidelines.

Primary In Situ Subgroup

Patients with a primary in situ tumor are required to satisfy all inclusion criteria as outlined above for the resected primary tumor population in addition to criteria specific for this patient subgroup as outlined below:

1. Primary in situ tumor of the colon or rectum not requiring surgical intervention prior to commencing chemotherapy
2. Minimally or asymptomatic primary tumor (without obstruction, perforation or active bleeding requiring transfusion).

4.3 Exclusion Criteria

Resected primary tumor population

A patient will be excluded in the resected primary tumor population if the answer to any of the following statements is "yes".

1. Previous chemotherapy for metastatic colorectal cancer.
2. Previous neoadjuvant or adjuvant chemotherapy completed within 6 months prior to commencement of study treatment.
3. Radiotherapy within 28 days prior to enrolment or from which patients have not yet recovered. Patients may be given palliative radiotherapy to peripheral sites (e.g. bone metastasis) within 28 days prior to enrolment but must have recovered from reversible acute side effects.
4. Patients with a history of non-colorectal malignancies are eligible if they have been disease-free for $\geq$ 5 years prior to study enrolment and are deemed by the physician to be at low risk for recurrence. However, patients with the following cancers are eligible if diagnosed and treated within the previous 5 years: carcinoma in situ of the colon, melanoma in situ, basal cell and squamous cell carcinoma of the skin, and carcinoma in situ of the cervix.
5. Presence of active inflammatory bowel disease.
6. History of gastrointestinal perforations.
7. Symptomatic or bulky peritoneal disease.
8. Evidence of bleeding diathesis or significant coagulopathy (in absence of therapeutic anticoagulation). History of a significant bleeding event or felt by the investigator to be at risk of such events.
9. Significant vascular disease (e.g. aortic aneurysm requiring surgical intervention).
10. Peripheral arterial thrombosis or other arterial thrombotic events within 6 months prior to commencement of study treatment.

11. Inadequately controlled hypertension (defined as values consistently > 150/100 mmHg despite use of at least three standard antihypertensive medications).
12. Prior history of hypertensive crisis or hypertensive encephalopathy.
13. Clinically significant (i.e. active) cardiovascular disease (e.g. NYHA Class II or greater congestive heart failure). See Section 18.7 for NYHA classification.
14. Concurrent major surgery unrelated to a primary in situ colorectal tumor and including invasive procedures defined as follows:
    a) Core biopsy or other minor procedure, excluding placement of a vascular access device, within 7 days prior to enrolment.
    b) Major surgical procedure, open biopsy, or significant traumatic injury within 28 days prior to enrolment.
    c) Planned major surgical procedures unrelated to a primary in situ colorectal tumor during the study.
15. Minor surgical procedures within 2 days prior to enrolment (including central venous access device placement for chemotherapy administration, tumor biopsies, needle aspirations, dental procedures).
16. Chronic active infection.
17. Investigational treatment within 28 days prior to enrolment.
18. Previous anti-angiogenic therapy (e.g. anti-VEGF or VEGF-R, tyrosine kinase inhibitor).
19. Previous anti-EGFR therapy (e.g. cetuximab or panitumumab).
20. Chronic daily treatment with oral corticosteroids (dose > 10 mg/day methylprednisolone equivalent) excluding inhaled steroids.
21. Known hypersensitivity to any of the study treatment.
22. Pregnant or lactating females (a serum pregnancy test to be assessed within 7 days prior to study treatment, or within 14 days with a confirmatory urine pregnancy test within 7 days prior to study treatment).
23. Women of childbearing potential (defined as < 2 years after last menstruation and not surgically sterile) not using appropriate method of contraception and not willing to use an effective method of contraception during the study and for 6 months after the last dose of study medication. Oral or injectable contraceptive agents cannot be the sole method of contraception.
24. Male patients must be surgically sterile or agree to use a barrier method of contraception.
25. Systemic inflammatory disorder (eg rheumatoid arthritis).

Primary In Situ Subgroup

Patients with a primary in situ tumor are required to comply with exclusion criteria as outlined above for the **resected primary tumor** population. In addition, patients with a primary in situ tumor will need to comply with the following exclusion criteria:

1. Prior endoscopic management of the current malignancy other than biopsy, including endoscopic stent placement, fulguration, or laser treatment.
2. Acute diverticulitis.
3. Presence of intra-abdominal abscess.
4. Active gastroduodenal ulcer(s).

4.4 Concomitant Medication and Treatment

Where applicable, patients on study should receive full supportive care including transfusion of blood and blood products, erythropoietin as clinically indicated, antibiotics for infective complications and anti-hypertensives for the management of hypertension etc. G-CSF can be used according to local practice guidelines. Bisphosphonates may be given according to their product license and routine clinical practice, at the investigator"s discretion. Anaphylaxis precautions should be observed during administration of bevacizumab and/or chemotherapy as per local practice. All concomitant medications will be reported in the electronic case report form (eCRF).

4.4.1 Antineoplastic treatments

Treatment with other concomitant, systemic anti-tumor agents, not identified in the protocol as study treatment or other concurrent investigational agents of any type is not permitted in this trial during treatment phase and before protocol defined 2nd disease progression. The patient may only be entered into another therapeutic clinical trial after withdrawal from ML25753 study treatment or study completion.

When patients enter follow-up phase, other antineoplastic treatment regimens may be commenced.

4.4.2 Surgery

In case the patient becomes eligible for curative resection of metastatic disease, bevacizumab treatment should be stopped at least 5 weeks before the planned day of surgery while chemotherapy may be continued at the discretion of the treating physician. If there is evidence of residual disease after resection, the patient may resume bevacizumab treatment (with / without chemotherapy) 28 days after surgery or complete wound healing until disease progression. In the absence of residual disease, post-operative ("adjuvant") treatment is at the discretion of the treating physician and the patient will be withdrawn from the study treatment and enter follow-up.

If bevacizumab is withheld for ≥ 6 weeks, the Roche Clinical Scientist should be contacted to discuss continuation of treatment or entry into survival follow-up.

4.4.3 Radiotherapy

Palliative radiotherapy is permitted during study treatment. Study treatment should be interrupted for the duration of radiotherapy and until recovery from acute reversible

effects. Patients who require palliative radiotherapy or initiation of bisphosphonates during study treatment should be evaluated carefully for possible disease progression before commencing these treatments.

If bevacizumab is withheld for ≥ 6 weeks, the Roche Clinical Scientist should be contacted to discuss continuation of treatment or entry into survival follow-up.

4.4.4 Anticoagulant and anti-platelet therapies

The use of oral coumarin-derived anticoagulants, unfractionated heparin or low molecular weight heparins and antiplatelet therapy is permitted at study entry (clopidogrel ≤ 75 mg/day; aspirin < 325 mg/day)]. Anti-coagulation for maintenance of patency of permanent indwelling i.v. catheters is permitted.

Therapeutic anticoagulation: Patients on therapeutic doses of anticoagulation (TA) treatment are permitted to enter the study. A recent pooled analysis of concurrent bevacizumab and TA in three large placebo-controlled clinical studies indicated that bevacizumab did not increase the risk of severe bleeding in cancer patients who received TA [46]. The use of full-dose oral or parenteral anticoagulants is permitted at study entry providing the INR or aPTT is within therapeutic limits (according to the medical standard in the institution) and the patient has been on a stable dose of anticoagulants for at least two weeks prior to commencement of study treatment. Patients who experience thromboembolic events during study treatment are permitted to receive TA and to remain on study medication.

Oral Coumarin-Derived Anticoagulants: Patients receiving concomitant capecitabine and oral coumarin-derived anticoagulants should have their anticoagulant status (INR or prothrombin time) monitored frequently in order to adjust the anticoagulant dose accordingly. Altered coagulation parameters and/or bleeding, including death, have been reported in patients taking capecitabine concomitantly with coumarin-derived anticoagulants such as warfarin and phenprocoumon. A pharmacokinetic (PK) interaction has been observed. The use of low molecular weight heparin (LMWH) instead of coumarin is at the discretion of the investigator.

Aspirin: Prophylactic low-dose aspirin is a recommended standard of care in patients at high-risk of cerebrovascular, coronary artery and peripheral vascular disease, and is supported by an extensive body of literature. As low-dose aspirin does not appear to increase the risk of grade 3-4 bleeding when used with bevacizumab plus chemotherapy, the use of prophylactic low-dose aspirin in patients who are at high risk of an arterial thromboembolic event is allowed. The use of mucosoprotective agents (i.e. omeprazole) should be considered.

4.4.4.1 Prophylactic Ca^{2+}/ Mg^{2+} infusions

Neuropathy caused by oxaliplatin alters patients‟ quality of life and may result in the postponement or interruption of the administration of chemotherapy. The use of Ca^{2+}/Mg^{2+} infusions before and after oxaliplatin administration has been shown in randomized multicenter trials to significantly reduce the incidence and severity of oxaliplatin-induced sensory neurotoxicity in colorectal cancer patients [47-49].

Prophylactic Ca^{2+}/Mg^{2+} infusions may be administered for the prevention of oxaliplatin-induced neurotoxicity at the discretion of the investigator e.g. calcium gluconate (1 g) and magnesium sulfate (1 g) in 100 mL of 5% glucose/dextrose administered over 15 minutes before and just after oxaliplatin (Ca^{2+}/Mg^{2+} salts may be administered in the same infusion bag).

Note: Bevacizumab infusions should not be administered or mixed with dextrose or glucose solutions.

4.5 Criteria for Premature Withdrawal

Subjects have the right to withdraw from the study at any time for any reason.

In the case that the patient decides to prematurely discontinue study treatment ("refuses treatment"), he/she should be asked if he/she can still be contacted for further information, especially regarding progression of their disease and subsequent anti-cancer therapies. The outcome of that discussion should be documented in both the medical records and in the eCRF. If lost to follow-up, the investigator should contact the patient or a responsible relative by telephone followed by registered mail or through a personal visit to establish as completely as possible the reason for the withdrawal. A complete final evaluation at the time of the patient"s withdrawal should be made with an explanation of why the patient is withdrawing from the study.

When applicable, patients should be informed of circumstances under which their participation may be terminated by the investigator without the patient"s consent.

The investigator has the right to withdraw patients from the study treatment in the event of:

- Intercurrent illness
- Adverse events
- Pregnancy
- Patient non-compliance with study procedures
- Colonic obstruction requiring stent placement
- Administrative reasons or other reasons.

Any administrative or other reasons for withdrawal must be documented and explained to the patient.If the reason for removal of a patient from the study is an Adverse Event, the principal specific event will be recorded in the eCRF. The patient should be followed until the Adverse Event has resolved, if possible. An excessive rate of withdrawals can render the study non-interpretable; therefore, unnecessary withdrawal of patients should be avoided. Should a patient decide to withdraw, all efforts will be made to complete and report the observations prior to withdrawal as thoroughly as possible.

4.6 Replacement Policy

4.6.1 For Patients

Patients will not be replaced in this study.

4.6.2 For Centers

A center may be replaced for the following administrative reasons:

- ***Excessively slow recruitment***: The sponsor has the right to close or replace centers that fail to recruit patients within a reasonable time-frame.
- ***Poor protocol adherence***: Repeated violation of inclusion and exclusion criteria, poor source data quality or poor data reporting, major delay in query reporting or repeated violation of safety reporting (format and timing) or any other serious violation of the procedures outlined in the protocol will give reason for closure or replacement of the affected center through the sponsor.

5. SCHEDULE OF ASSESSMENTS AND PROCEDURES

Please refer to Table 2 detailing the Schedule of Assessments.

Table 2 Schedule of Assessments

	SCREENING^	BASELINE	DAY 3	TREATMENT PERIOD		FOLLOW -UP	
**Week or cycle**	**-2**	**-1**	**3**	**Phase A treatment***	**Phase B treatment ***	**Safety follow-up within 30 days after 1st disease progression AND / OR End of treatment (EoT) safety follow up visit within 30 days after 2nd disease progression or Withdrawal**	**Survival follow-up 12 weekly visits (± 5 days) Until End of Study**
**Study day / Study Assessments**	**-14 to Day 0**	**-7 to Day 0**		**Study visits every 8 or 9 weeks (± 3 days)****			
Informed consent	X						
Confirmation of eligibility criteria	X	X					
Consent of collection of tumor block	X						
Demographics[a]	X						
Medical History[b]	X						
Physical examination[c]		X		Every study visit		X	
Vital signs[d]	X	X		Every study visit		X	
ECG	X						
Hematology[e]		X		Every study visit, per institutional guidelines at other visits		X	
Biochemistry[f]		X		Every study visit, per institutional guidelines at other visits		X	
Coagulation tests[g]		X		Every study visit, per institutional guidelines at other visits		X	

Urinalysis[h]		X		As per institutional guidelines		X	
Pregnancy test[i]		X		As clinically indicated			
Blood plasma sampling[j]		X	X	See notes for plasma sampling schedule		X	
Colorectal Cancer History[k]	X						
Prior cancer treatment history[l]	X						
Cardiovascular risk assessment[m]		X		As clinically indicated			
Tumor assessment or evaluation of progression[n]		X		Every study visit		X	
Concomitant medications [o]	X	X		Every study visit		X	X
WHO performance status		X		Every study visit, per institutional guidelines at other visits		X	
Modified Charlson Comorbidity Index		X		Every study visit		X	
Quality of Life questionnaires[p]		X		Every study visit		X	X
Primary tumor-related clinical assessment for primary in situ tumor subgroup[q]		X		As clinically indicated		X	
Adverse Events[r]	X	X		Every study visit as per institutional guidelines		X	X[s]
Survival status[t]							X[u]
Drug administration[v]				Every treatment cycle:<br><br>Bevacizumab 7.5mg/kg IV on day 1 every 3 weeks in combination with XELOX<br>*Or*<br>Bevacizumab 5.0mg/kg IV on day 1 every 2 weeks in combination with mFOLFOX6	Every treatment cycle: -<br><br>Bevacizumab 5.0mg/kg IV on day 1 every 2 weeks in combination with FOLFIRI		

* Patients will have a safety follow up visit no later than 30 days after the last dose of study treatment in Phase A, and another EoT safety assessment no later than 30 days after the last dose of study treatment in Phase B. If patients are withdrawn from the study or have withdrawn consent, the last date of study treatment and reasons why treatment has been withdrawn

early will be recorded in the eCRF.

** To coincide with chemotherapy regimens. Laboratory tests may be performed within 5 days prior to administration of study treatment. Results must be available for review prior to administration of study treatment.

^ Patients who are screen failures may be rescreened at any time. All screening procedures must be repeated regardless of the time elapsed since the previous screening period.

[a] Includes age, gender.

[b] Relevant medical history only. Includes confirmation of histological diagnosis of carcinoma of the colon or rectum.

[c] Includes weight measurement. Height is only measured at baseline.

[d] Includes blood pressure, pulse rate and body temperature measurements at baseline and screening visits. Subsequent treatment visits require blood pressure as a minimum.

[e] Hematology: perform tests as per local standard of care and results should be available for review prior to administration of study drug. Tests must include hemoglobin, RBC, platelet count, WBC with differential count (neutrophils, lymphocytes, monocytes, eosinophils, basophils) and ANC. Tests may be performed within 14 days of baseline visit.

[f] Biochemistry: perform tests as per local standard of care and results should be available for review prior to administration of study drug. Tests must include sodium, potassium, chloride, calcium, blood urea nitrogen (BUN), uric acid, total protein (an unscheduled lab test for direct bilirubin should be completed and reviewed in the case of abnormal total bilirubin) and albumin, alkaline phosphatase, AST/SGOT, ALT/SGPT, GGT, LDH, total bilirubin, blood glucose, creatinine, C-Reactive Protein (CRP) and CEA. Tests may be performed within 14 days of baseline visit.

[g] Coagulation tests: as per local standard of care and results should be available for review prior to administration of study drug. For patients on full-dose coumarin derivatives at baseline INR/aPTT must be checked and be within therapeutic limits (according to institutional standards) at least before each study drug administration. Tests may be performed within 14 days of baseline visit.

[h] Urinalysis: by dipstick and within 14 days prior to commencing study treatment and as per institutional guidelines during treatment phase.

[i] Pregnancy test (women of child-bearing potential only): Serum pregnancy test within 7 days prior to the first study treatment or within 14 days with a confirmatory urine pregnancy test within 7 days prior to the first study treatment.

[j] Blood for plasma (one ~6.0 mL sample in EDTA) will be obtained at the following time points: Baseline (day -1 → -7 prior to any study treatment), day 3 of cycle 1 (Phase A treatment), day 14/21 dependent on chemotherapy selected (prior to cycle 2 of Phase A treatment), end of Phase A treatment (end of treatment safety follow-up visit) and end of Phase B treatment (end of treatment safety follow-up visit).

[k] Colorectal cancer history including metastatic sites, history and occurrence of resection of primary tumor or liver-only metastatic disease

[l] Includes chemotherapy, surgery and radiation.

[m] At baseline only and as per clinically indicated thereafter: To include age, aspirin use, blood pressure and antihypertensive treatment, total cholesterol and lipid lowering treatment, blood sugar (on diet/ oral hypoglycemics/ insulin), smoking history, and previous ATE (angina pectoris, myocardial infarction, transient ischemic attack, cerebrovascular accident or peripheral vascular disease).

[n] Method of tumor assessment should be consistent throughout all visits and performed until disease progression. Baseline tumor assessment to be performed within 28 days prior to commencement of treatment.

[o] During survival follow-up visits, only antineoplastic agents including biological agents are to be recorded in the eCRF.

[p] Quality of Life assessments include EuroQol 5-D, AQol-8D and FACT-C at baseline then every 8 or 9 weekly study visit (prior to treatment) and upon disease progression. Completion of these questionnaires at each survival follow-up visit is optional.

[q] For patients with a primary in situ tumor: primary tumor-related clinical assessment including tumor site, history or presence of abdominal inflammation, prior pelvic or abdominal radiation, presence or absence of active peptic ulcer disease.

[r] During screening, AEs are not recorded in the eCRF unless they are SAEs which are related to protocol-mandated procedures. After screening, all AEs suspected to be related to

bevacizumab must be recorded from the time of first bevacizumab administration. AEs (including AEs of special interest refer to Section 7.2.2) will be collected at every treatment visit and 8-9 weekly study visit and recorded in the eCRF. AEs suspected to be related to bevacizumab occurring during the study will be reported from consent and up to 6 months after the last bevacizumab dose. Bevacizumab treatment should be permanently discontinued in patients experiencing any of the adverse events listed in Table 3 of Section 6.2.1. AEs unrelated to bevacizumab occurring during the study will be reported from consent and up to 28 days after last bevacizumab dose. All AEs related to bevacizumab or otherwise, should be monitored until resolution or stabilization.

[s] SAEs suspected to be related to bevacizumab must be collected and reported regardless of the time elapsed from the last study drug administration, even if the study has been closed. Existing events must be monitored until resolution or stabilization.

[t] Following 2nd disease progression, the patient will enter follow-up for survival status and use of other antineoplastic agents including biological agents. Patients who are withdrawn from the study prior to 1st disease progression will also enter follow-up for progression of their disease, survival status and use of antineoplastic agents.

[u] Survival follow-up continues for a minimum of 24 months from the date of first study drug administration in Phase A.

[v] Bevacizumab treatment will continue until progression (PD), unmanageable toxicity or patient withdrawal. In Phase A, each treatment cycle will be either every three weeks in combination with XELOX or every two weeks in combination with mFOLFOX6. In Phase B, FOLFIRI plus bevacizumab will be administered every two weeks. If a patient experiences toxicities specific to either oxaliplatin or irinotecan, patients should continue treatment with a fluoropyrimidine and bevacizumab. If all chemotherapy is suspended temporarily due to toxicity, treatment with bevacizumab alone may continue until chemotherapy is restarted or until the decision is made to permanently discontinue chemotherapy. If all chemotherapy must be discontinued permanently due to related toxicity, bevacizumab should be discontinued and the patient should enter follow up. AEs pre, during and post infusions will be reported in the eCRF.

Notes If there is suspicion of disease progression based on clinical or laboratory findings before the next scheduled assessment, an unscheduled assessment should be performed according to institutional guidelines and captured in the eCRF.

5.1 Screening Examination and Eligibility Screening Form

Written informed consent for participation in the study must be obtained before performing any study specific screening tests or evaluations. Informed Consent Forms for enrolled patients and for patients who are not subsequently enrolled will be maintained at the study site.

A screening examination should be performed –within 14 days prior to the baseline visit. Patients who fulfill all the inclusion and none of the exclusion criteria will be accepted into the study.

Patients who are deemed screen failures may be rescreened at any time. There is no limit to the number of times a patient may be re-screened. With the exception of consent, all screening procedures must be repeated regardless of the time elapsed since the previous screening period. Biomarker plasma sampling is a mandatory criterion for the inclusion of patients in this trial (see to Section 5.4.2). Patients must provide consent to the collection of tumor samples for central banking and future analysis.

An Eligibility Screening Form (ESF) documenting the investigator"s assessment of each screened patient with regard to the protocol"s inclusion and exclusion criteria is to be completed by the investigator.

A patient enrollment and identification code list and screen failure log must be maintained by the investigator. Additionally, screen failures will be recorded in the eCRF with specific inclusion or exclusion criteria the patient did not meet for entry into the study.

5.2 Procedures for Enrollment of Eligible Subjects

Once a patient has met all the inclusion and none of the exclusion criteria, and completed all screening procedures as per Schedule of Assessments (see Table 2), the patient will be enrolled into the study to receive first-line therapy with XELOX or mFOLFOX6 plus bevacizumab in accordance with the local standard of care and PBS guidelines.

5.3 Clinical Assessments and Procedures

Refer to Table 2 detailing the schedule of assessments.

5.3.1 Screening Procedures

Within 14 days prior to first dosing of bevacizumab the following assessments will be completed:

- Informed consent.
- Confirmation of eligibility criteria.
- Consent to tumor block collection for tumor banking and biomarker analysis.
- Demographics, including age, gender.

- Relevant medical history, including confirmation of histological diagnosis of carcinoma of the colon or rectum.
- Vital signs including blood pressure, pulse rate and body temperature.
- Electrocardiogram (ECG).
- Colorectal cancer history including metastatic sites, history and occurrence of resection of primary tumor or liver-only metastatic disease.
- Prior cancer treatment history including chemotherapy, surgery, radiation.
- All concomitant medications.
- Adverse events.

5.3.2 Baseline Procedures

Within 7* days prior to first dose of bevacizumab the following assessments will be completed:

- Confirmation of eligibility criteria.
- Physical examination, including weight and height.
- Vital signs including blood pressure, pulse rate and body temperature.
- Cardiovascular risk assessment including age, aspirin use (at baseline and during the study), blood pressure and antihypertensive treatment, total cholesterol and lipid lowering treatment, blood sugar (on diet/oral hypoglycemics/insulin), smoking history, and previous ATE (angina pectoris, myocardial infarction, transient ischemic attack, cerebrovascular accident or peripheral vascular disease).
- Blood sampling for hematology and biochemistry must be performed as per local guidelines and results must be available for review prior to administration of study treatment.*Tests may be performed within 14 days prior to the first dose of bevacizumab.
  - Hematology tests must include hemoglobin, RBC, platelet count, WBC with differential count (neutrophils, lymphocytes, monocytes, eosinophils, basophils) and ANC.
  - Biochemistry tests must include sodium, potassium, chloride, calcium, blood urea nitrogen (BUN), uric acid, total protein and albumin, alkaline phosphatase, AST/SGOT, ALT/SGPT, GGT, LDH, total bilirubin (an unscheduled test for direct bilirubin should be completed in the case abnormal total bilirubin), blood glucose, creatinine, C-Reactive Protein (CRP) and CEA.

- An additional 6.0 ml blood sample will be collected in EDTA for plasma isolation (refer to section 5.4.2).

- *Coagulation tests as per local standard of care and within 14 days prior to study treatment. The results must be available for review prior to administration of study treatment. For patients on full-dose coumarin derivatives at baseline INR/aPTT must be checked and be within therapeutic limits (according to institutional standards) at least before each study drug administration.
- *Urinalysis (dipstick) within 14 days prior to study treatment.
- Pregnancy test must be carried out in women of child-bearing potential. Serum pregnancy test within 7 days prior to the first study treatment or within 14 days with a confirmatory urine pregnancy test within 7 days prior to the first study treatment.
- *Tumor assessment: method of tumor assessment should be consistent throughout all study visits and performed until disease progression. Tumor assessment must be performed within 28 days prior to first dose of bevacizumab.
- Concomitant medications.
- WHO performance status.
- Modified Charlston Comorbidity Index.
- Quality of Life questionnaires including EuroQol 5-D, AQol-8D and FACT-C.
- Adverse events.
- For patients with a primary in situ tumor: primary tumor-related clinical assessment including tumor site, history or presence of abdominal inflammation, prior pelvic or abdominal radiation, presence or absence of active peptic ulcer disease.

5.3.3 Treatment Period

Treatment is to commence within 7 days of the baseline visit. Patients will then attend study specific visits every 8 or 9 weeks during the entire treatment period, to coincide with chemotherapy regimen, which will take place either three-weekly or two weekly (dependent on chemotherapy chosen) during Phase A treatment and two-weekly during Phase B treatment, as described in Section 6.1.1.

The following assessments will be completed at each study specific visits:

- Physical examination, including weight.
- Vital signs including blood pressure.
- Tumor assessment and evaluation of progression as described in Section 5.3.6. Method of tumor assessment must be consistent throughout all visits and performed until disease progression.

- Documentation of 1st disease progression at any study-specific visit following entry into Phase A treatment will determine entry into Phase B treatment.

- Blood sampling for hematology, biochemistry must be performed within 5 days prior to administration of study treatment as per local standard of care and clinical indication. Results must be available for review prior to administration of study treatment.
  - Hematology tests must include hemoglobin, RBC, platelet count, WBC with differential count (neutrophils, lymphocytes, monocytes, eosinophils, basophils) and ANC.
  - Biochemistry tests must include sodium, potassium, chloride, calcium, blood urea nitrogen (BUN), uric acid, total protein and albumin, alkaline phosphatase, AST/SGOT, ALT/SGPT, GGT, LDH, total bilirubin (an unscheduled lab test for direct bilirubin should be completed and reviewed in the case of abnormal total bilirubin), blood glucose, creatinine, C-Reactive Protein (CRP) and CEA.
- Coagulation tests as per local standard of care and clinical indication. When applicable, the results must be available for review prior to administration of study drug. For patients on full-dose coumarin derivatives INR/aPTT must be checked and be within therapeutic limits (according to institutional standards) at least before each study drug administration.
- An additional 6.0 ml blood sample will be collected in EDTA for plasma isolation at the following treatment visits:
  - Day 3 of cycle 1 (Phase A treatment)
  - Day 14/21 of Phase A treatment dependent on chemotherapy selected (prior to cycle 2 of Phase A treatment).
- Concomitant medications.
- WHO performance status.
- Adverse Events.
- Modified Charlston Comorbidity Index.
- Quality of Life questionnaires including EuroQol 5-D, AQol-8D and FACT-C.
- <u>For patients in the primary in situ subgroup</u>: primary tumor-related clinical assessment including tumor site, presence of abdominal inflammation, presence or absence of active peptic ulcer disease.

The following assessment will be completed as per institutional guidelines or as clinically indicated:

- Urinalysis (dipstick).
- Pregnancy test in women of child-bearing potential.
- Cardiovascular risk assessment including age, aspirin use (at baseline and during the study), blood pressure and antihypertensive treatment, total cholesterol and lipid lowering treatment, blood sugar (on diet/oral hypoglycemics/insulin), smoking history, and previous ATE (angina pectoris, myocardial infarction, transient ischemic attack, cerebrovascular accident or peripheral vascular disease).
- For primary in situ patients: primary tumor related clinical assessments: including presence of abdominal inflammation or peptic ulcer disease.

Unscheduled tests should be performed for any of the above assessments if clinically indicated.

The following assessments will be completed for each treatment cycle visits (either 2 or 3 weekly dependent on the chemotherapy selected for Phase A and 2 weekly during Phase B) which will occur between the 8-9 weekly study visits:

- Blood sampling for hematology, biochemistry and coagulation (as per institutional guidelines or as clinically indicated)
- Adverse Events (can be obtained via telephone)
- Concomitant medications (can be obtained via telephone)
- Vital signs including blood pressure

5.3.4 Safety follow-up visit

All patients must undergo a safety assessment as soon as possible, but no later than 30 days after the last dose of study treatment in Phase A, and an end of treatment (EoT) safety assessment no later than 30 days after the last dose of study treatment in Phase B.

If patients are withdrawn from the study or have withdrawn consent, the last date of study treatment and reasons why treatment has been withdrawn early will be recorded in the eCRF. The safety follow-up and EoT safety follow up assessments will include:

- Physical examination, including weight.
- Vital signs including blood pressure, pulse rate and body temperature.
- Blood sampling for hematology, biochemistry and plasma isolation.

  - Hematology tests must include hemoglobin, RBC, platelet count, WBC with differential count (neutrophils, lymphocytes, monocytes, eosinophils, basophils) and ANC.
  - Biochemistry tests must include sodium, potassium, chloride, calcium, blood urea nitrogen (BUN), uric acid, total protein and albumin, alkaline phosphatase, AST/SGOT, ALT/SGPT, GGT, LDH, total bilirubin (an unscheduled lab test for direct bilirubin should be completed and reviewed in the case of abnormal total bilirubin), blood glucose, creatinine, C-Reactive Protein (CRP) and CEA.
  - Blood sampling will include a 6.0 mL blood sample for plasma isolation to be collected at the end of Phase A treatment (EoT safety follow-up visit) and end of Phase B treatment (EoT safety follow-up visit).

- Coagulation tests.
- Urinalysis (dipstick).
- Concomitant medications.
- WHO performance status.
- Adverse events.
- Modified Charlston Comorbidity Index.
- Quality of Life questionnaires including EuroQol 5-D, AQol-8D and FACT-C.
- For patients in the primary in situ subgroup: primary tumor-related clinical assessment including tumor site, presence of abdominal inflammation, presence or absence of active peptic ulcer disease.

5.3.5 Survival follow-up visits

After patients complete the end of treatment safety assessment, all patients will be followed for survival information every 12 weeks (±5 days) until death, loss to follow-up, or study closure. The study will close 24 months after the date of first study treatment of the last patient enrolled.

Assessments during survival follow-up can be obtained via telephone or in-clinic visits and are to include:

- Survival status
- Concomitant medications: only antineoplastic agents including biological agents are to be recorded in the eCRF during the survival follow-up visits.
- Adverse events: SAEs and AEs suspected to be related to bevacizumab must be reported. Existing events must be monitored until resolution or stabilization.

- The completion of Quality of Life questionnaires including EuroQol 5-D, AQol-8D and FACT-C are optional at this visit.
- Tumor assessment and evaluation of progression as described in Section 5.3.6. Method of tumor assessment must be consistent throughout all visits and performed until disease progression. Patients who have not progressed whilst on study treatment will be followed for progressive disease in order to be evaluable for the primary endpoint.

5.3.6 Tumor Response and Disease Progression

Method of tumor assessment should be consistent throughout all study visits and performed until disease progression.

Response Evaluation Criteria in Solid Tumors (RECIST) criteria are considered suboptimal methods to predict benefits from bevacizumab-containing regimens [12, 50, 51], to monitor treatment response, and to assess whether and when to discontinue treatment. RECIST criteria were developed to assess tumor shrinkage after cytotoxic chemotherapy and may be limited in assessing response to biologic agents such as bevacizumab, which have a cytostatic mechanism of action. Following administration of bevacizumab fibrosis, necrosis or cavitation of the tumor/metastasis without a change in size are sometimes observed [32, 51, 52]. Liver metastases from mCRC patients treated with bevacizumab which demonstrated a major pathologic response (< 50% residual cancer cells remaining) were characterized by replacement of tumor cells by fibrosis [50]. CT scans alone are not able to differentiate between vital tumor tissue or for example fibrotic tissue [52]. Improvements in PFS have been reported in patients with mCRC after treatment with bevacizumab regardless of a RECIST-defined response [52].The effect of bevacizumab may be underestimated when using tumor shrinkage alone as a response assessment tool and caution should be exercised if response/progression are assessed using only radiological assessment methods.

5.3.6.1 Tumor Response

Tumor response will be based on the investigator assessment of the response as per routine clinical practice.

5.3.6.2 Disease Progression

In this study, disease progression is determined according to standard practice based on radiological, biochemical (carcinoembryonic antigen [CEA]) or clinical factors. Determination of disease progression should be unequivocal and is defined as <u>ANY</u> of the following:

- An unequivocal and clinically meaningful increase in the size of known tumors.
- The appearance of one or more new lesions.
- Death due to disease without prior objective documentation of progression.
- Elevated CEA accompanied by other radiological or clinical evidence of progression.
- Symptomatic deterioration.

„Unequivocal progression" is considered as an overall level of substantial worsening in the patient"s disease of a magnitude such that the investigator would feel it important to change therapy. Details regarding the investigator"s determination of disease progression will be documented in the eCRF.

The appearance of new malignant lesions denotes disease progression. The finding of a new lesion should be unequivocal: i.e. not attributable to differences in scanning technique, change in imaging modality or findings thought to represent something other than tumor. For equivocal findings of progression (e.g. very small and uncertain new lesions; cystic changes or necrosis in existing lesions), treatment may continue until the next scheduled study visit. If at the next scheduled study visit, progression is confirmed, the date of progression should be the earlier date when progression was suspected.

Symptomatic deterioration is defined as a global deterioration in health status attributable to the disease requiring a change in therapy without objective evidence of progression. Disease progression determined by symptomatic deterioration should be a rare exception and every effort should be made to document the objective progression even after discontinuation of treatment.

CEA elevation alone should not be defined as disease progression unless accompanied by clear radiological, clinical or symptomatic progression. Patients on treatment who are well but have rising CEA levels should continue study treatment until radiological, clinical or symptomatic progression of disease. Caution should be exercised when interpreting a rising CEA level during the first 4-6 weeks of new therapy, as spurious early rises may occur, especially after oxaliplatin.

Disease progression will be assessed at every study visit during the treatment period (Phase A and Phase B) and at the EoT safety visits, as described in Sections 5.3.3, 5.3.4 and 5.3.5.

Primary tumor-related clinical assessment

For patients in the primary in situ tumor sub-study, baseline and post-baseline tumor-related clinical assessments should include primary tumor site, history or presence of abdominal inflammation, prior pelvic or abdominal radiation, presence or absence of active peptic ulcer disease.

5.3.7 Performance status

Performance Status (PS) will be measured using the WHO Performance Status Scale (see Section 18.3), as per criterion retained in PBS recommendations.

It is recommended, where possible, that a patient"s PS will be assessed by the same person throughout the study.

Performance status will be assessed at baseline, at every study visit during the treatment phase (per institutional guidelines at other visits), and at the EoT safety follow up visits.

5.3.8 Clinical Safety Assessments

The NCI CTC-AE version 4.0 will be used to evaluate the clinical safety of the treatment in this study. Subjects will be assessed for adverse events at each study visit and as necessary throughout the study. Determination of relatedness to bevacizumab is at the discretion of the investigator.

A relevant medical history, including confirmation of histological diagnosis of carcinoma of the colon or rectum, colorectal cancer treatment history (including metastatic sites, history and occurrence of resection of primary tumor or liver-only metastasis), concurrent illnesses, concomitant medications and prior treatments for cancer, as well as demographics will be performed at screening.

Physical examination and vital signs (including blood pressure, pulse rate and body temperature) will be performed at baseline, at every visit during the treatment period and during the follow-up period. Height will only be measured at baseline.

An electrocardiogram (ECG) should be performed on all patients at screening. A cardiovascular risk assessment including age, aspirin use, blood pressure and antihypertensive treatment, total cholesterol and lipid lowering treatment, blood sugar (on diet/ oral hypoglycaemics/ insulin), smoking history, and previous ATE (angina pectoris, myocardial infarction, transient ischaemic attack, cerebrovascular accident or peripheral vascular disease) will be performed at baseline and throughout the treatment period as clinically indicated.

Information in relation to all concomitant medications will be collected at screening, baseline, and throughout the entire study period. During survival follow-up visits, only antineoplastic agents including biological agents are to be recorded in the eCRF.

The modified Charlson Comorbidity Index will also be established at baseline, at every study visit during the treatment phase, and at the EoT safety follow-up visits.

5.4 Laboratory Assessments

5.4.1 Standard laboratory assessments

All laboratory assessments will be performed locally and results are to be available for review prior to administration of study treatment.

Normal ranges for the study laboratory parameters performed at site must be provided to Roche for entry into the eCRF prior to commencement of patient enrolment.

The total volume of blood loss for laboratory assessments will be approximately 15 mL per visit.

The following laboratory safety assessments and procedures will be performed according to the Schedule of Assessments (Table 2):

- Hematology: hemoglobin, red blood cells (RBC), platelet count, white blood cells (WBC) with differential count (neutrophils, lymphocytes, monocytes, eosinophils, basophils), and absolute neutrophil count (ANC).

- Biochemistry: sodium, potassium, calcium, chloride, blood urea nitrogen (BUN), uric acid, total protein and albumin, alkaline phosphatase, AST/SGOT, ALT/SGPT, GGT, LDH, total bilirubin (direct bilirubin in the case abnormal total bilirubin), blood glucose, creatinine, C-Reactive Protein (CRP) and CEA.
- Urinalysis at baseline and as per institution guidelines.
- Serum pregnancy test within 7 days prior to the first study treatment or within 14 days with a confirmatory urine pregnancy test within 7 days prior to the first study treatment

5.4.2 Plasma samples for proteomic sub-study

A substudy will be undertaken to evaluate the relationship between acute-phase plasma proteins and therapeutic outcomes following treatment with bevacizumab combined with standard fluoropyrimidine-based chemotherapy(ies).

Blood samples for plasma isolation, (approximately 6 mL for each sample collection) will be collected from every patient at baseline, day 3, cycle 1 of Phase A treatment, day 14/21 of Phase A dependent on the chemotherapy selected (prior to drug administration), end of Phase A treatment and end of Phase B treatment.

5.4.3 Tumor Tissue Banking

Formalin-fixed tumor tissue embedded in paraffin blocks (or parts of tumor blocks) will be collected when it becomes available. The first sample would be taken from the original primary tumor, or from the tumor tissue taken before the patient joined study ML25753. If any additional tumor tissue becomes available as part of the patient"s routine medical care during study ML25753, samples of this tissue are also to be collected. These tissues may be from the primary tumor, a metastatic site if the primary tumor is unavailable, or, if possible, from both primary tumor and a metastatic site.

Tumor tissue blocks will be used to set up a tissue microarray (TMA) for IHC analysis and potentially for the extraction of DNA and RNA. Markers of IL-6-mediated inflammatory pathways, markers of angiogenesis (VEGF, VEGFR1/R2, neuropilin-1, CD31), markers of tumor biology, tumor necrosis, vascularity, cell turnover, and expression of molecules in the VEGF families and their signaling pathway molecules and other possible markers considered relevant to efficacy or safety outcomes may be assessed.

5.5 Patient Reported Outcome(s)

5.5.1 Quality of Life Assessments

The EuroQol 5-D (EQ-5D), AQolL-8D and FACT-C will be used for quality of life assessment in the study. Patients will be asked to complete each questionnaire at baseline, at each 8 or 9 weekly study visit during treatment phases and at the end of treatment safety follow up visit.

- EQ-5D is a standardised measure of health status providing a simple, generic measure of health for clinical and economic appraisal. It provides a simple descriptive profile

and a single index value for health status that can be used in the clinical and economic evaluation of health care as well as in population health surveys (see Section 18.4).

- AQoL-8D provides a global utility score and consists of 8 separately scored dimensions including Independent Living, Life Satisfaction, Mental Health, Coping, Relationships, Self Worth, Pain and Senses (http://www.aqol.com.au).
- FACT-C is one part of the FACIT Measurement System, which comprehensively assesses the health-related QoL of cancer patients and patients with other chronic illnesses. It is composed of 27 items of the general version of the Functional Assessment of Cancer Therapy (FACT-G) as a general core QoL measure and has a disease-specific subscale containing nine colorectal cancer-specific items (see Section 18.6).

6. INVESTIGATIONAL MEDICINAL PRODUCT

In this study, bevacizumab administered beyond 1st disease progression (Phase B) is considered to be the "investigational study drug". Bevacizumab administered as Phase A treatment is considered to be standard-of-care "non-investigational drug". XELOX, mFOLFOX6 and FOLFIRI are considered standard of care "non-investigational combination drug". Collectively, they will be known as the „study treatment". Study treatment will be administered in two phases defined as follows:

- *Phase A treatment* is defined as study treatment (XELOX or mFOLFOX6 plus bevacizumab) administered from commencement of the study until 1st disease progression. Phase A treatment includes all study treatment including periods of fluoropyrimidine/bevacizumab maintenance therapy up until 1st progression. Phase A treatment will be discontinued upon documentation of 1st disease progression.
- *Phase B treatment* is defined as study treatment (FOLFIRI plus bevacizumab) administered after 1st disease progression until 2nd disease progression is documented. Phase B treatment includes periods of fluoropyrimidine/bevacizumab maintenance therapy.

Study treatment in each phase will continue until documented disease progression, unmanageable toxicity or patient withdrawal from the study.

6.1 Dose and Schedule of IMP

6.1.1 Phase A

During Phase A: PBS supplied-bevacizumab will be administered in combination with either XELOX or mFOLFOX6.

6.1.1.1 XELOX + bevacizumab

Frequency: every 3 weeks

Drug dosages:

- Oxaliplatin: 130 mg/m^2 iv day 1
- Capecitabine: 1000 mg/ m^2 p.o twice daily days 1 - 14

- Bevacizumab: 7.5 mg/kg iv day 1

If oxaliplatin is discontinued, capecitabine and bevacizumab should be continued (maintenance therapy) until 1st disease progression or unmanageable toxicity.

6.1.1.2 mFOLFOX6 + bevacizumab

Frequency: every 2 weeks

Drug dosages:

- Oxaliplatin: 85 mg/m^2 iv day 1
- Leucovorin (LV): 400 mg/m^2 iv day 1 (*investigators may elect to use low-dose leucovorin i.e. either 20 mg/m^2 or 50 mg total dose*)
- Fluorouracil (5-FU): 400 mg/m^2 iv day 1
- Fluorouracil (5-FU): 2400 mg/m^2 continuous iv infusion over 46 hours day 1
- Bevacizumab: 5.0 mg/kg iv day 1

It is recommended that bevacizumab infusion is to be administered as per regulatory approved guidelines (refer to Product Information and Section 6.2.5).

If oxaliplatin is discontinued, 5-FU/LV and bevacizumab should be continued (maintenance therapy) until 1st disease progression or unmanageable toxicity. If oxaliplatin is discontinued, 5-FU/LV can be replaced with capecitabine and the 7.5 mg/kg once every 3 weeks bevacizumab schedule at the discretion of the investigator.

Reintroduction of oxaliplatin after discontinuation is not permitted in either Phase A or Phase B. Oxaliplatin based therapy can be re-initiated at investigator discretion after entry into the follow-up phase.

Further information regarding capecitabine dose calculation according to body surface area (BSA) can be found in Section 18.11.

6.1.2 Phase B

During Phase B: bevacizumab will be administered in combination with FOLFIRI on a 2-weekly basis as follows:

- Irinotecan**:** 180 mg/m^2 iv day 1
- Leucovorin (LV): 400 mg/m^2 iv day 1 (investigators may elect to use low-dose leucovorin i.e. either 20 mg/m^2 or 50 mg total dose)
- Fluorouracil (5-FU): 400 mg/m^2 iv day 1
- Fluorouracil (5-FU): 2400 mg/m^2 continuous iv infusion over 46 hours day 1
- Bevacizumab: 5.0 mg/kg iv day 1.

If irinotecan discontinuation is required, 5-FU/LV plus bevacizumab should be continued (maintenance therapy) until 2nd disease progression or unmanageable toxicity.

In the event of irinotecan discontinuation, 5-FU/LV can be replaced with capecitabine and treatment with capecitabine and bevacizumab 7.5 mg/kg can be resumed once every 3 weeks until 2nd disease progression at the discretion of the investigator.

6.2 Dose Modifications, Interruptions and Delays

6.2.1 Bevacizumab

The patient"s body weight at baseline is to be used to calculate the required dose, and there should be no dose modifications unless the patient"s body weight changes by ± 10% from baseline.

The bevacizumab dose (7.5 mg/kg or 5.0 mg/kg for Phase A or 5.0 mg/kg for Phase B) should not be reduced or modified due to toxicity.

Any toxicities associated or possibly associated with bevacizumab treatment should be managed according to standard medical practice.

As described below, bevacizumab treatment may be either temporarily or permanently suspended in the case of hypertension, proteinuria, thrombosis/embolism, hemorrhage, CHF or wound healing complications in addition to any other serious bevacizumab-related toxicity (Grade 3 or 4). Criteria for treatment modification and guidelines for the management of toxicities are summarized in Table 3. If adverse events occur that necessitate withholding bevacizumab, the dose will remain unchanged once treatment resumes.

Bevacizumab should be temporarily withheld in the event of febrile Grade 4 neutropenia and/or Grade 4 thrombocytopenia (regardless of the relationship to treatment), since these conditions are predisposing factors for an increased bleeding tendency.

If bevacizumab is withheld for ≥ 6 weeks due to toxicity, the Roche Clinical Scientist should be contacted to discuss continuation of treatment or entry into survival follow-up.

Patients should be assessed clinically for toxicity prior to, during, and after each infusion. If unmanageable toxicity due to bevacizumab occurs at any time during the study, treatment with bevacizumab should be discontinued.

Patients who discontinue treatment with bevacizumab because of toxicity will enter survival follow-up, with an EoT safety visit within 30 days of the last dose of bevacizumab.

Bevacizumab treatment should be permanently discontinued in patients experiencing any of the following events:

- Grade 2 – 4 pulmonary hemorrhage
- Grade 2 – 4 CNS hemorrhage (specify anatomical location)
- Grade 4 non-pulmonary or CNS hemorrhage (specify anatomical location)
- ATE event (any grade; specify anatomical location)

- VTE (symptomatic Grade 4)
- Grade 4 left ventricular systolic dysfunction
- Nephrotic syndrome (grade 4 proteinuria)
- GI perforation (any grade)
- Tracheoesophageal fistula (any grade)
- Grade 4 fistula (specify anatomical location)
- Grade $\geq$ 2 bowel obstruction that has not fully recovered despite medical or surgical intervention
- Wound dehiscence requiring medical or surgical intervention
- Reversible posterior leukoencephalopathy syndrome (RPLS) (any grade) confirmed by MRI
- Any Grade 4 event suspected to be related to bevacizumab
- Uncontrolled hypertension (BP consistently > 150/100 mmHg despite use of up to three standard antihypertensive medications) grade 3 or above

Table 3 Bevacizumab Dose Management Due to Adverse Events

Event	Action to be Taken
**Hypertension**	
Grade 1 or 2	No bevacizumab dose modifications.
Grade 3	If not controlled to 150/100 mmHg with medication, discontinue bevacizumab.
Grade 4 (including hypertensive encephalopathy)	Discontinue bevacizumab.
**Hemorrhage**	
Grade 1 or 2 non-pulmonary or non-CNS events	No bevacizumab dose modifications.
Grade 3 non-pulmonary or non-CNS hemorrhage	Patients who are also receiving full-dose anticoagulation will be discontinued from receiving bevacizumab. All other patients will have bevacizumab held until all of the following criteria are met:<br>• The bleeding has resolved and hemoglobin is stable.<br>• There is no bleeding diathesis that would increase the risk of therapy.<br>• There is no anatomic or pathologic condition that significantly increases the risk of hemorrhage recurrence.<br>Patients who experience a repeat Grade 3 hemorrhagic event will be discontinued from receiving bevacizumab.
Grade 4 non-pulmonary or non-CNS hemorrhage	Discontinue bevacizumab.
Grade 1 pulmonary or CNS hemorrhage	Patients who are also receiving full-dose anticoagulation will be discontinued from receiving bevacizumab. All other patients will have bevacizumab held until all of the following criteria are met:<br>• The bleeding has resolved and hemoglobin is stable.<br>• There is no bleeding diathesis that would increase the risk of therapy.<br>• There is no anatomic or pathologic condition that significantly increases the risk of hemorrhage recurrence.
Grade 2, 3, or 4 pulmonary or CNS hemorrhage	Discontinue bevacizumab.

CNS = central nervous system; GI = gastrointestinal; INR = international normalized ratio; LMWH = low molecular weight heparin; MRI = magnetic resonance imaging; UPC = urine protein:creatinine.

Table 3 (cont'd) Bevacizumab Dose Management Due to Adverse Events

Event	Action to be Taken
**Venous Thromboembolic Event**	
Grade 1 or 2	No bevacizumab dose modifications.
Grade 3 or asymptomatic Grade 4	Hold bevacizumab treatment. If the planned duration of full-dose anticoagulation is < 2 weeks, bevacizumab should be held until the full-dose anticoagulation period is over. If the planned duration of full-dose anticoagulation is > 2 weeks, bevacizumab may be resumed during the period of full-dose anticoagulation if all of the following criteria are met:<br>• The patient must have an in-range INR (usually between 2 and 3) if on warfarin; LMWH, warfarin, or other anticoagulant dosing must be stable prior to restarting bevacizumab treatment.<br>• The patient must not have had a Grade 3 or 4 hemorrhagic event while on anticoagulation.
Symptomatic Grade 4	Discontinue bevacizumab.
**Arterial Thromboembolic Event** (new onset, worsening, or unstable angina, myocardial infarction, transient ischemic attack, cerebrovascular accident, and any other arterial thromboembolic event)	
Any grade	Discontinue bevacizumab.
**Congestive Heart Failure (left ventricular systolic dysfunction)**	
Grade 1 or 2	No bevacizumab dose modifications.
Grade 3	Hold bevacizumab until resolution to Grade ≤ 1.
Grade 4	Discontinue bevacizumab.
**Proteinuria**	
Grade 1 or 2	No bevacizumab dose modifications.
Grade 3 (UPC ratio > 3.5, 24-hr urine collection > 3.5 g/24 hr)	Hold bevacizumab treatment until Grade ≤ 2, as determined by either UPC ratio ≤ 3.5 or 24-hr collection ≤ 3.5 g.
Grade 4 (Nephrotic syndrome)	Discontinue bevacizumab.
**GI Perforation**	
Any grade	Discontinue bevacizumab.
**Fistula**	
Any grade TE fistula	Discontinue bevacizumab.
Grade 4 fistula other than TE	Discontinue bevacizumab.

CNS = central nervous system; GI = gastrointestinal; INR = international normalized ratio; LMWH = low molecular weight heparin; MRI = magnetic resonance imaging; UPC = urine protein:creatinine.

Table 3 (cont'd) Bevacizumab Dose Management Due to Adverse Events

Event	Action to be Taken
**Bowel Obstruction**	
Grade 1	Continue patient on study for partial obstruction <u>not</u> requiring medical intervention.
Grade 2	Hold bevacizumab for partial obstruction requiring medical intervention. Patient may restart upon complete resolution.
Grade 3 or 4	Hold bevacizumab for complete obstruction. If surgery is necessary, patient may restart bevacizumab after full recovery from surgery, and at Investigator's discretion.
**Wound Dehiscence**	
Any grade (requiring medical or surgical therapy)	Discontinue bevacizumab.
**Reversible Posterior Leukoencephalopathy**	
Any grade (confirmed by MRI)	Discontinue bevacizumab.
**Other Unspecified Bevacizumab-Related Adverse Events**	
Grade 3	Hold bevacizumab until recovery to Grade ≤ 1
Grade 4	Discontinue bevacizumab.

CNS = central nervous system; GI = gastrointestinal; INR = international normalized ratio; LMWH = low molecular weight heparin; MRI = magnetic resonance imaging; UPC = urine protein:creatinine.

Additional information regarding suggested treatment algorithm for the management of bevacizumab-induced hypertension and pairing of anti-hypertensive agents can be found in Section 18.9 and Section 18.10, respectively.

6.2.2 Drug holidays

- If deemed clinically appropriate by the investigator, study treatment may be temporarily suspended for up to four weeks <u>for reasons other than toxicity and/or surgery</u> during each of Phase A and Phase B (eight weeks holiday in total across both Phases A and B).
- If study treatment is suspended for more than the permitted four weeks <u>for reasons other than toxicity and/or surgery</u> in either treatment phase, patients will be withdrawn from treatment and enter follow-up.

6.2.3 Bevacizumab monotherapy

- If all chemotherapy is suspended temporarily due to toxicity, treatment with bevacizumab alone may continue until chemotherapy is restarted or until the decision is made to permanently discontinue all chemotherapy.
- If all chemotherapy must be permanently discontinued due to related toxicity, bevacizumab should be discontinued and the patient should enter follow-up.

6.2.4 Surgical procedures/wound healing complications

Bevacizumab therapy should be withheld for an interval of at least four weeks (28 days) before conducting elective surgery. In the case of unplanned surgical procedures, bevacizumab should be stopped as soon as the indication for surgery is identified. Emergency surgery should be performed as appropriate without delay after a careful risk benefit assessment. Bevacizumab therapy should be restarted ≥ 28 days and ≤ 42 days following major surgery. In patients who experience wound healing complications during bevacizumab treatment, bevacizumab should be withheld until the wound is fully healed. If the wound is not fully healed within 42 days, bevacizumab treatment should be discontinued and patient should continue in follow-up. Continuation of study treatment in patients who have had bevacizumab therapy delayed for more than 2 treatment cycles due to surgical procedures or wound healing must be discussed with the Roche Clinical Scientist.

6.2.5 Infusion Reactions

Bevacizumab infusion should be interrupted in patients who develop dyspnea or clinically significant hypotension. Patients who experience an NCI CTCAE Grade 3 or 4 allergic reaction/hypersensitivity, adult respiratory distress syndrome, or bronchospasm (regardless of grade) will be discontinued from bevacizumab treatment.

The infusion should be slowed to ≤ 50% or interrupted for patients who experience any infusion-associated symptoms not specified above. When the patient"s symptoms have completely resolved, the infusion may be continued at ≤ 50% of the rate prior to the reaction and increased in 50% increments every 30 minutes if well tolerated. Infusions may be restarted at the full rate during the next cycle. Vital signs should be evaluated pre, during and post-bevacizumab infusions.

6.2.6 Chemotherapy

Local standard practice will drive dose reduction or schedule modifications of chemotherapeutic regimens. Every effort should be made to maintain dose intensity. If possible, toxicities should be managed symptomatically. In case of toxicity-related chemotherapy dose reduction no dose re-escalation is permitted.

Premedications (anti-emetics, hydration, antihistamines, corticosteroids, etc.), dosage, and administration for chemotherapy should be according to institutional standards and the respective Product Information.

6.2.6.1 Oxaliplatin toxicity

Discontinuation of oxaliplatin should be considered if ≥ Grade 2 neurotoxicity develops. If oxaliplatin is discontinued, treatment should be continued with capecitabine or 5-FU/LV and bevacizumab until 1st disease progression. Suggested dose modifications are given in Table 4.

Table 4 Suggested dose modification for oxaliplatin-induced neuropathy

	Duration of toxicity		
Toxicity	***1 – 7 days***	***> 7 days***	***Persisting between cycles***
Cold-induced dysesthesia	No change	No change	No change
Paresthesia	No change	No change	Reduce oxaliplatin dose by 25%
Paresthesia with pain	No change	Reduce oxaliplatin dose by 25%	Stop oxaliplatin and continue Capecitabine and bevacizumab
Paresthesia with functional impairment	No change	Reduce oxaliplatin dose by 50%	Stop oxaliplatin and continue Capecitabine and bevacizumab

Prophylactic use of Ca^{2+}/Mg^{2+} infusions to attenuate oxaliplatin-mediated neurotoxicity is permitted at the discretion of the investigator (see Section 4.4.4.1).

Reintroduction of oxaliplatin during the study is not permitted. Oxaliplatin may be reintroduced at the investigator"s discretion after documentation of 2nd disease progression.

6.2.6.2 *Capecitabine toxicity*

Dose-limiting side effects of capecitabine therapy include diarrhea, abdominal pain, nausea, stomatitis and hand-foot syndrome. Most adverse events are reversible and do not require permanent treatment withdrawal, although it may be necessary to interrupt treatment or reduce the dose, depending on their severity. If non-hematological Grade 2, 3 or 4 toxicity occurs, Table 5 should be used for guidance with regard to treatment interruptions, discontinuations and dose adjustments.

Table 5 Suggested capecitabine dose adjustments for non-hematological toxicities

	Grade 2 and 3	Grade 4
1st occurrence	Interrupt treatment, then on recovery to grade 0-1, continue capecitabine at 75% of original dose with prophylaxis where possible	Discontinue treatment permanently, unless Investigator considers it to be in the best interest of the patient to continue at 50% of original dose
2nd occurrence	Interrupt treatment then on recovery to grade 0-1, continue capecitabine at 50% of original dose	
3rd occurrence	Stop treatment permanently- unless it is considered by the Investigator to be in the best interest of the patient to stay on treatment	

As the incidence of myelosuppression with capecitabine is extremely low, no dose reductions of capecitabine are required for hematological toxicities unless Grade ≥ 4 neutropenia or Grade 4 thrombocytopenia. If this occurs, capecitabine should be withheld until these toxicities are < Grade 2.

Should capecitabine dose be reduced at any stage during treatment, it should not be reintroduced at the dose that caused toxicity.

6.2.6.3 5-FU and irinotecan toxicity

Suggested dose modifications for irinotecan and 5-FU in the event of the first appearance of toxicities are given in Section 18.12. If toxicity reoccurs, despite a prior dose reduction, the dose of 5-FU can be further adjusted if the investigator considers this to be in the best interest of the patient.

6.3 Preparation and Administration of IMP

For all bevacizumab medication administered during Phase A treatment, the approved regulatory (PBS) requirements will be applicable. During Phase B treatment, clinical trial bevacizumab stock will be supplied by Roche Products Pty Limited.

6.3.1 Preparation of Bevacizumab

Bevacizumab does not contain any antimicrobial preservative, therefore, care must be taken to ensure the sterility of the prepared solution. A qualified individual responsible for dispensing the study drug should aseptically prepare the correct dose according to the protocol schedule.

For Phase B treatment, the individual will write the date dispensed and patient number on the study drug vial label and on the Drug Accountability logs provided by Roche. This individual will also record in the accountability logs, the study drug batch or lot number received by each patient during the study.

After withdrawal of an appropriate volume of bevacizumab (for either 7.5mg/kg or 5mg/kg dose) based on the patient"s dose level, bevacizumab should be diluted to a minimum volume of 100 mL with 0.9% (w/v) sodium chloride solution prior to

administration as an i.v. infusion. The concentration of the final bevacizumab solution should be kept within the range of 1.4-16.5 mg/mL.

Parenteral medicinal products should be inspected visually for particulate matter and discoloration prior to administration. If any particulate matter or discoloration is seen, the bevacizumab vial should be discarded, and another one should be used, and it should be reported to the monitor immediately.

Any unused portion of bevacizumab left in the vial should be discarded as the product contains no preservatives.

6.3.2 Administration of Bevacizumab

Bevacizumab should be administered according to the approved Australian Product Information. The initial bevacizumab dose should be delivered over 90 minutes as an IV infusion, with subsequent infusions over 60 minutes and then 30 minutes, as tolerated.

Bevacizumab should not be administered as an intravenous push or bolus.

If the patient develops infusion-related adverse events while receiving bevacizumab then that dose and subsequent doses should be infused over a longer time. For example if there are problems when bevacizumab is given over 30 minutes then it should subsequently be given over 60 minutes, and if there were problems when bevacizumab is given over 60 minutes then it should subsequently be given over 90 minutes.

When the infusion bag containing bevacizumab is empty, 50 mL of 0.9% sodium chloride should be added to the iv bag, or an additional 50 mL bag of 0.9% sodium chloride administered and the infusion continued for a volume equal to that of the tubing to ensure complete delivery of the trial drug. The sodium chloride infusion is not included in the study drug infusion time.

Depending on the chosen chemotherapy, pre-medication should be administered according to local practice.

6.3.3 Bevacizumab Storage

Vials are to be stored in a refrigerator at 2 – 8°C**.** Vials are to be kept in the outer carton due to light sensitivity. DO NOT SHAKE AND DO NOT FREEZE VIAL CONTENTS.

Chemical and physical in-use stability has been demonstrated for 48 hours at 2 – 30°C in sodium chloride 9 mg/mL (0.9%) solution for injection. To reduce microbiological hazard, the product should be used immediately. If not used immediately, in-use storage times and conditions are the responsibility of the user and would normally not be longer than 24 hours at 2°C to 8°C, unless dilution has taken place in controlled and validated aseptic conditions.

6.3.4 Incompatibilities

No incompatibilities between bevacizumab and polyvinyl chloride or polyolefin bags or infusion sets have been observed.

Bevacizumab infusions should not be administered or mixed with glucose solutions.

6.4 Formulation, Packaging and Labeling

This section applies to Phase B bevacizumab only.

The packaging and labeling of bevacizumab will be in accordance with all Roche standard and local requirements and conducted according to Good Manufacturing Practice.

Bevacizumab is provided as a clear to slightly opalescent, colorless to pale brown, sterile liquid for intravenous infusion in single-use vials that are preservative-free. Bevacizumab will be supplied either in 5 mL (100 mg, 25 mg/mL) glass vials with a 4 mL fill, or in 20 mL (400 mg, 25 mg/mL) glass vials with a 16 mL fill. The formulation contains sodium phosphate, trehalose, polysorbate 20, and Sterile Water for Injection (SWFI).

Upon arrival of investigational products at the site, site personnel should check them for damage and verify proper identity, quantity, integrity of seals and temperature conditions, and report any deviations or product complaints to the monitor immediately upon discovery.

6.5 Blinding and Unblinding

Not applicable, the study is an open label study.

6.6 Accountability of IMP and Assessment of Compliance

6.6.1 Accountability of IMP

This section applies to Phase B bevacizumab only. Accountability of Phase A bevacizumab should be undertaken as local pharmacy practice.

The investigator is responsible for the control of drugs under investigation. Adequate records for the receipts (e.g. Drug Receipt Record) and disposition (e.g. Drug Dispensing Log) of the study drug must be maintained. Accountability and patient compliance will be assessed by maintaining adequate "drug dispensing" and destruction records.

Accurate records must be kept for each study drug provided by the sponsor. These records must contain the following information:

- Documentation of drug shipments received from the sponsor (date received and quantity);
- Disposition of unused study drug not dispensed to patient;
- A Drug Dispensing Log must be kept up to date and should contain the following information:
- the identification of the patient to whom the study medication was administered;
- the date(s), quantity of the study medication administered to the patient, vial strength and batch number administered to the patient at each treatment visit.

All records and drug supplies must be available for inspection by the Monitor at every monitoring visit.

The inventory must be available for inspection by the Monitor. All supplies, including partially used or empty containers and copies of the dispensing & inventory logs, must be kept at site for review by the Roche monitor and returned to the Roche monitor at the end of the study, unless alternate destruction has been authorized by Roche, or required by local or institutional regulations (Section 6.7).

Any chemotherapy drugs supplied by the site pharmacy must be checked for consistency between the eCRF and clinical records.

6.6.2 Assessment of Compliance

Subject compliance will be assessed by maintaining adequate study drug dispensing records. The investigator is responsible for ensuring that dosing is correctly administered in compliance with the protocol. Delegation of this task must be clearly documented and approved by the investigator.

6.7 Destruction of the IMP

Local or institutional regulations may require immediate destruction of used investigational medicinal product (IMP) for safety reasons e.g., cytotoxicity. In these cases, it may be acceptable for investigational site staff to destroy dispensed IMP before a monitoring inspection provided that source document verification is performed on the remaining inventory and reconciled against the documentation of quantity shipped, dispensed, returned and destroyed. Written authorization must be obtained from the sponsor at study start up before destruction.

Written documentation of destruction must contain the following:

- Identity (batch numbers or patient numbers) of investigational product(s) destroyed
- Quantity of investigational product(s) destroyed
- Date of destruction (date discarded in designated hazardous container for destruction)
- Method of destruction (the site must provide the sponsor with documentation of their institutional policy and procedures for handling and disposing of hazardous drugs)
- Name and signature of responsible person who discarded the investigational product in a hazardous container for destruction

7. SAFETY INSTRUCTIONS AND GUIDANCE

7.1 Adverse Events and Laboratory Abnormalities

7.1.1 Clinical AEs

According to the International Conference of Harmonization (ICH), an AE is any untoward medical occurrence in a patient or clinical investigation subject administered a

pharmaceutical product and which does not necessarily have a causal relationship with this treatment. An AE can therefore be any unfavorable and unintended sign (including an abnormal laboratory finding), symptom, or disease temporally associated with the use of a medicinal (investigational) product, whether or not considered related to the medicinal (investigational) product. Pre-existing conditions which worsen during a study are to be reported as AEs.

7.1.1.1 Intensity

Intensity of all adverse events will be graded according to the NCI Common Terminology Criteria for Adverse Events v 4.0 (CTCAE) on a five-point scale (Grade 1 to 5) and reported in detail on the eCRF (see Section 18.8).

Adverse events not listed on the CTCAE should be graded as follows:

CTC Grade	Equivalent To:	Definition
Grade 1	Mild	Discomfort noticed but no disruption of normal daily activity
Grade 2	Moderate	Discomfort sufficient to reduce or affect daily activity; no treatment or medical intervention is indicated although this could improve the overall well-being or symptoms of the patient
Grade 3	Severe	Inability to work or perform normal daily activity; treatment or medical intervention is indicated in order to improve the overall well-being or symptoms; delaying the onset of treatment is not putting the survival of the patient at direct risk.
Grade 4	Life threatening/ disabling	An immediate threat to life or leading to a permanent mental or physical conditions that prevents work or performing normal daily activities; treatment or medical intervention is required in order to maintain survival.
Grade 5	Death	AE resulting in death

7.1.1.2 Drug – Adverse Event relationship

The causality relationship of study treatment to the adverse event will be assessed by the investigator as either:

Yes or No

If there is a reasonable suspected causal relationship to the study treatment, i.e. there are facts (evidence) or arguments to suggest a causal relationship, drug-event relationship should be assessed as **Yes**.

The following criteria should be considered in order to assess the relationship as **Yes:**

- Reasonable temporal association with drug administration

- It may or may not have been produced by the patient"s clinical state, environmental or toxic factors, or other modes of therapy administered to the patient.
- Known response pattern to suspected drug
- Disappears or decreases on cessation or reduction in dose
- Reappears on rechallenge

The following criteria should be considered in order to assess the relationship as **No**:

- It does not follow a reasonable temporal sequence from administration of the drug.
- It may readily have been produced by the patient"s clinical state, environmental or toxic factors, or other modes of therapy administered to the patient.
- It does not follow a known pattern of response to the suspected drug.
- It does not reappear or worsen when the drug is readministered.

Determination of relatedness to bevacizumab is at the discretion of the investigator.

7.1.1.3 Serious Adverse Events (Immediately Reportable to Roche)

A serious adverse event is any experience that suggests a significant hazard, contraindication, side effect or precaution. It is any Adverse Event that at any dose fulfills at least one of the following criteria:

- is fatal; (results in **death**; NOTE: death is an outcome, not an event)
- is Life-Threatening (NOTE: the term "Life-Threatening" refers to an event in which the patient was at immediate risk of death at the time of the event; it does not refer to an event which could hypothetically have caused a death had it been more severe).
- required in-patient hospitalization or prolongation of existing hospitalization;
- results in persistent or significant disability/incapacity;
- is a congenital anomaly/birth defect;
- is medically significant or requires intervention to prevent one or other of the outcomes listed above

****The term sudden death should be used only when the cause is of a cardiac origin as per standard definition. The terms death and sudden death are clearly distinct and must not be used interchangeably**.

The study will comply with all local regulatory requirements and adhere to the full requirements of the ICH Guideline for Clinical Safety Data Management, Definitions and Standards for Expedited Reporting, Topic E2. (see Section 18.2).

7.1.1.4 *Progression of Underlying Malignancy*

Progression of underlying malignancy is not reported as an adverse event if it is clearly consistent with the suspected progression of the underlying cancer as determined by protocol. Hospitalization due solely to the progression of underlying malignancy should NOT be reported as a serious adverse event. Clinical symptoms of progression may be reported as adverse events if the symptom cannot be determined as exclusively due to the progression of the underlying malignancy, or does not fit the expected pattern of progression for the disease under study. See Section 5.3.6 for the definition of disease progression.

If there is any uncertainty about an adverse event being due only to the disease under study, it should be reported as an AE or SAE.

7.1.2 Treatment and Follow-up of AEs

Information in relation to AEs/SAEs will be collected from the date of informed consent.

SAEs caused by a protocol mandated intervention will be collected from informed consent and prior to initiation of any study medications.

AEs (including AEs of special interest) suspected to be related to bevacizumab occurring during the study will be reported from the first dose of bevacizumab and up to 6 months after the last bevacizumab dose.

SAEs (including AEs of special interest) suspected to be related to bevacizumab must be collected and reported regardless of the time elapsed from the last study drug administration, even if the study has been closed.

All AE/SAEs related to bevacizumab or otherwise, will be monitored until resolution or stabilization.

Related AEs: Follow until one of the following occurs:

- Resolved or improved to baseline
- Relationship is reassessed as unrelated
- Death
- Start of new anti-cancer regimen
- Investigator confirms that no further improvement can be expected
- Clinical or safety data will no longer be collected, or final database closure

All AE/SAEs unrelated to bevacizumab occurring during the study will be reported from consent and up to 28 days after last bevacizumab dose. Unrelated AEs/SAEs of Grade 3 or above will be monitored until resolution or stabilization.

<u>Unrelated severe or life threatening AEs:</u> Follow until one of the following occurs:

- Resolved or improved to baseline
- Severity improved to Grade 2
- Death
- Start of new anti-cancer regimen
- Investigator confirms that no further improvement can be expected
- Clinical or safety data will no longer be collected, or final database closure

<u>Unrelated Grade 1 or Grade 2 AEs:</u> Follow until 28 days after the last dose of bevacizumab. The final outcome of each adverse event must be recorded on the eCRF.

7.1.3 Laboratory Test Abnormalities

All laboratory test results will be recorded in the eCRF.

Any treatment-emergent abnormal laboratory result which is clinically significant, i.e., meeting one or more of the following conditions, should be recorded as a single diagnosis on the adverse event page in the eCRF:

- Accompanied by clinical symptoms;
- Leading to a change in study medication (e.g. dose modification, interruption or permanent discontinuation);
- Requiring a change in concomitant therapy (e.g. addition of, interruption of, discontinuation of, or any other change in a concomitant medication, therapy or treatment).

Any laboratory result abnormality fulfilling the criteria for a serious adverse event (SAE) should be reported as such, in addition to being recorded as an AE in the eCRF.

7.1.3.1 Follow-up of Abnormal Laboratory Test Values

In the event of medically significant unexplained abnormal laboratory test values, the tests should be repeated and followed up until they have returned to the normal range and/or an adequate explanation of the abnormality is found. If a clear explanation is established it should be recorded on the eCRF.

7.1.4 Toxicity Management Guidelines

Refer to Section 6.2 for guidance on recommended treatment modifications should toxicity to all or part of chemotherapy regimen occur.

7.2 Handling of Safety Parameters

7.2.1 Reporting of Serious Adverse Events (immediately reportable)

Any clinical adverse event or abnormal laboratory test value that is *serious* (as defined in Section 7.1.1.3 above) must be reported to the sponsor **within *24 hours*** of the investigator becoming aware of the event (expedited reporting). The investigator must

complete the *SAE Reporting Form (gcp_for004369)* and forward it to the SAE Responsible.

After informed consent, but prior to initiation of study medications, only SAEs caused by a protocol-mandated intervention will be collected (e.g., SAEs related to invasive procedures such as biopsies, medication washout, or no treatment run-in). After first study medication, all SAEs must be reported.

Related Serious Adverse Events MUST be collected and reported regardless of the time elapsed from the last study drug administration, even if the study has been closed. After study closure, report any SAEs/AEs that the investigator considers having a causal relationship with bevacizumab, regardless of time elapsed since last bevacizumab dose, via the spontaneous reporting procedures). Suspected Unexpected Serious Adverse Reactions (SUSARs) are reported to investigators at each site and associated IRB/IEC when the following conditions occur:

- The event must be a SAE.
- There must be a certain degree of probability that the event is an adverse reaction from the administered drug.
- The adverse reaction must be unexpected, that is to say, not foreseen in the TGA approved Australian PI (for an authorized medicinal product) or the Investigator"s Brochure (for an unauthorized medicinal product).

When all patients at a particular site are off treatment as defined by the protocol:

- only individual SUSAR reports originating in that particular trial will be forwarded to the site and associated IRB/IEC on an expedited basis;
- individual SUSARs considered to be a significant safety issue and/or which result in Roche recommending a change to the Informed Consent Form (ICF), will be reported in an expedited manner to all investigators and IRBs/IECs;
- SUSAR reports originating from other trials using the same IMP will be provided as six monthly SUSAR Reports (SSRs) to investigators and IRBs/IECs where long-term follow-up studies are carried out, unless they are considered significant.

Unrelated Serious Adverse Events must be collected and reported during the study and for up to 28 days after the last dose of study medication.

This study adheres to the definition and reporting requirements of ICH Guideline for Clinical Safety Data Management, Definitions and Standards for Expedited Reporting, Topic E2. Complete information can be found in Appendix 1.

7.2.2 Reporting of Non-serious Events of Special Interest

Non-serious events of Special Interest must be reported to Roche by the investigator as outlined in Section 7.2.1. The non-serious adverse events to be reported are:

- GI perforation - any Grade
- GI Fistula / Abscess – any Grade
- Bleeding – Grade 3 or above
    - Bleeding (CNS) - Grade 3 or above
    - Bleeding (Non-CNS) - Grade 3 or above
- Wound healing complications/dehiscence – Grade 3 or above
- ATE – any Grade
- VTE – Grade 3 or above
- Hypertension – Grade 3 or above
- RPLS – any Grade
- Febrile neutropenia – any Grade
- Proteinuria – Grade 4 or above
- CHF – Grade 3 or above

7.2.3 Pregnancy

A female patient must be instructed to stop taking the test "drug" and immediately inform the investigator if she becomes pregnant during the study. The investigator should report all pregnancies within **24 hours** to the sponsor, using the *Clinical Trial Pregnancy Reporting Form [gcp_for000023]*. The investigator should counsel the patient, discuss the risks of continuing with the pregnancy and the possible effects on the fetus. Monitoring of the patient should continue until conclusion of the pregnancy. Pregnancies occurring up to 90 days after the completion of the study medication must also be reported to the investigator.

Pregnancy occurring in the partner of a male patient participating in the study should be reported to the investigator. The investigator must report the pregnancy to the sponsor within 24 hours after consent is obtained from the pregnant partner using the *Pregnant Partner Data Release Form [gcp_for000186]*. The partner should be counseled, the risks of continuing the pregnancy discussed, as well as the possible effects on the fetus. Monitoring of the patient should continue until conclusion of the pregnancy.

NOTE: The investigator should fill out a Pregnancy Reporting Form, [gcp_for000023], only if the pregnant partner has signed a Pregnant Partner Data Release Form, [gcp_for000186].

7.3 Warnings and Precautions

No evidence available at the time of the approval of this study protocol indicated that special warnings or precautions were appropriate, other than those noted in the Investigators" Brochure or Product Information.

8. STATISTICAL CONSIDERATIONS AND ANALYTICAL PLAN

8.1 Primary Variable

The primary endpoint, PFS, is defined as the time from the start of initial treatment to documentation of first disease progression or death from any cause, whichever occurs first.

8.2 Secondary Variables

8.2.1 Secondary Safety Variables

- Adverse Events, classified by System Organ Class and Preferred Term, including:

  - All Adverse Events
  - Events that are Treatment Emergent relative to start of Phase A.
  - Events that are Treatment Emergent relative to start of Phase B.
  - Severity of Events according to the CTCAE v4.0 grading system.
  - Adverse Events of Grade 3-5 Severity.
  - Seriousness and reason for determination of seriousness.
  - Investigator determination of relatedness to study therapy.
  - Adverse events of special interest, including:
    - GI perforation - any Grade
    - GI Fistula / Abscess – any Grade
    - Bleeding – Grade 3 or above
      - Bleeding (CNS) - Grade 3 or above
      - Bleeding (Non-CNS) - Grade 3 or above
    - Wound healing complications/dehiscence – Grade 3 or above
    - ATE – any Grade
    - VTE – Grade 3 or above
    - Hypertension – Grade 3 or above
    - RPLS – any Grade
    - Febrile neutropenia – any Grade
    - Proteinuria – Grade 4 or above
    - CHF – Grade 3 or above
  - In patients with a primary in situ tumor: Serious Adverse Events related to the primary tumor.

- Concomitant Medication usage, classified according to ATC code.
- Laboratory Tests
- Electrocardiograms
- WHO Performance Status
- Charlson Morbidity Index
- Study Drug Exposure.

8.2.2 Secondary Efficacy variables

Efficacy variables will include:

- PFS until 1st progression (PFS)
- PFS during Phase B (PFS-B)
- Time to Failure of Strategy (TFS)
- Duration of Disease Control (DDC)
- OS from the start of treatment (OS)
- Survival beyond 1st progression (SBP)
- OS from the start of Phase B (OS-B)
- Best overall response rate (ORR) as assessed by investigator in each treatment phase and overall.
- Rate of liver resection.

Progression-free survival until 1st progression (PFS) is defined as the time from the start of initial treatment to documentation of first disease progression or death from any cause, whichever occurs first. Subjects without documented progression and for whom there exists eCRF evidence that evaluations have been made, will be censored at the date of the last tumor assessment when the patient was known to be progression free. Subjects without post baseline tumor assessments but known to be alive will be censored at the time of treatment initiation.

Progression-free survival in Phase B (PFS-B) is defined as the time from the start of Phase B treatment to documentation of second disease progression or death from any cause, whichever occurs first.

Duration of Disease Control (DDC) is defined as PFS + PFS-B. If a patient does not enter Phase B, then DDC is defined as PFS.

Time to Failure of Strategy (TFS) is defined as time from the start of initial treatment to documentation of 1st disease progression without entering Phase B, or 2nd disease progression having entered Phase B. Patients still on therapy at the end of the study, or withdrawn from treatment without evidence of progression, will be censored at the last date they were known to be progression-free.

Survival Beyond first Progression (SBP) is defined as the time from the date of first disease progression to death of any cause.

Overall Survival (OS) is defined as the time from the start of initial treatment to the date of death, regardless of the cause of death. Subjects who were alive at the time of the analysis will be censored at the date of the last follow up assessment. Subjects without follow up assessment will be censored at the day of last dose and patients with no post baseline information will be censored at the time of enrolment.

Overall Survival in Phase B (OS-B) is defined as the time from the start of treatment in Phase B to death of any cause.

Best Overall Response Rate (ORR) is defined as Complete or Partial response to therapy in either treatment phase as determined by the investigator.

Rate of liver resection defined as the proportion of patients undergoing potentially curative liver resection.

8.3 Statistical and Analytical Methods

8.3.1 Statistical Method

All testing of statistical hypotheses will be conducted at two-sided alpha of 0.05.

8.3.1.1 Covariates

Unless otherwise stated, associations will be adjusted for the following default covariates:

- WHO Performance Status (0 vs. 1)
- Metastatic disease in the liver (Yes/No)
- Number of different sites of metastatic disease (≤ 3 vs. > 3)
- Presence of metastatic disease in the liver with no other sites involved (Yes/No)

The justification for this choice of covariates is that these measure general health (WHO Performance Status) and disease burden are known to be associated with progression free survival independently of the inflammatory processes being modeled by the NLR. It was decided not to adjust for hypoalbuminemia, which was found by Chua *et al.* to be associated with PFS, or anemia, because it is believed they may be modeling same inflammatory process as NLR. Age was not included because it was considered likely to be correlated and with, and less predictive of outcome than, Performance Status.

Other measures of tumor burden, extent of involvement of metastatic sites, and laboratory analytes will be investigated in exploratory analyses.

8.3.1.2 Populations analyzed

The Full Analysis Set will include all patients who receive at least one dose of bevacizumab.

The “Primary In Situ Tumor population” will include all patients in the Full Analysis Set with a primary in situ tumor.

The “Resected Primary Tumor population” will include all patients in the Full Analysis Set without a primary in situ tumor.

8.3.1.3 Interim Analysis

Interim analyses may be conducted to provide interim reports, for example to provide final results on each phase once all data has been collected for a particular phase or to check the distribution of baseline variables.

Regular reviews of safety in the primary in situ tumor population will be performed.

The study will not be terminated or altered on the basis of the results of any interim analyses, and no endpoints will be analysed before enrolment is complete, therefore no adjustment for alpha is planned.

8.3.2 Primary Analysis

The primary analysis will be a Cox Proportional Hazards model of baseline NLR on Progression Free Survival, adjusted for the default covariates (see 8.3.1.1 above). The primary analysis will be conducted on the Full Analysis Set.

Baseline NLR will be analysed in the primary analysis as a dichotomous variable (≤5 vs. >5).

The null hypothesis to be tested using the Wald chi-squared statistic is $H_0 : \beta_1 = 0$, where β_1 is the regression coefficient for the effect of baseline NLR in the primary model.

8.3.3 Safety Data Analysis

Adverse event variables will be presented in frequency tables for all populations during each Phase of treatment and overall. Events occurring between Phase A and Phase B will be summarized as part of Phase A.

For the events of particular interest, tables will additionally be presented for the total number of episodes. Every occurrence of an event in any patient will be counted in the total number of episodes but successive reports of an identical event in the same phase (treatment, follow-up) will be combined (concatenated) into one episode if the end date of the earlier event was the same as the start date of the later event, or if the end date of the earlier event was missing.

The WHO status, and Charlson Comorbidity Index will be summarized at each time point.

The incidence of Grade 3-5 Adverse Events and Events of Special Interest will be cross-tabulated with age and Charlson Comorbidity Index.

Laboratory data will be summarised with descriptive statistics at each sampling time point, and shift frequency tables will be presented. Nadir values will be summarized as continuous variables by phase.

Vital signs, electrocardiograms, physical exams, and cardiovascular risk assessments will be reported in listings.

Treatment exposure will be summarized as the number of cycles received by each patient, as the percentage of the planned dose of each agent given at each cycle, and by starting and cumulative dose.

8.3.4 Other Analyses

8.3.4.1 Demographic and Disease State summaries

Descriptive statistics will be provided for the baseline disease-state and demographic patient characteristics outlined in the Schedule of Events (Section 5) in the Full Analysis Set, the Primary In Situ Tumor Population and the Resected Primary Tumor Population. Comparisons will be provided between patients with NLR ≤ 5 and those with NLR >5 using Fisher"s Exact test for categorical variables and a t-test for continuous variables. These comparisons will be considered exploratory.

8.3.4.2 Robustness of the primary analysis

The confounding effect of baseline factors that were not included in the default set of covariates will be investigated in the primary model using the Full Analysis Set. Confounders to be investigated will include age, sex, baseline laboratory measurements, disease state characteristics, and medical history. The robustness of the primary analysis will be investigated by including these confounders in the primary model on their own and in combination.

8.3.4.3 Secondary efficacy and safety variables

Descriptive statistics will be provided for the secondary safety and efficacy variables for the Full Analysis Set, the Primary In Situ Tumor population and the Resected Primary Tumor Population.

The association between post-baseline changes in NLR and PFS and OS will be assessed in the Full Analysis Set using proportional hazards modeling. The associations with early normalization will be assessed by comparing patients whose NLR has normalized with patients whose NLR was ≤5 at baseline and at the first post-baseline measurements. The associations with longitudinal changes in NLR will be assessed using NLR status as a time-varying covariate.

8.3.4.4 Quality of Life Analysis

Quality of life assessments on the EQ-5D, AQoL-8D and FACT-C will be used to derive pre-specified QoL scores according to the QoL manuals.

Quality of life assessments and changes from baseline will be descriptively summarized by study visit. Utility scores from the EQ-5D and AQoL-8D will be used to calculate Quality-Adjusted survival estimates.

8.3.4.5 Exploratory Relationships

The following relationships will be investigated by modeling and cross-tabulation:

- The relationship between blood-based markers of systemic inflammation and therapeutic outcomes (PFS, OS and Adverse Event rates), including the following:

  - the relationship between liver-derived acute phase plasma proteins and PFS
  - the relationship between the neutrophil/lymphocyte (NLR) and platelet/lymphocyte (PLR) ratios and PFS;
  - the influence of the modified Glasgow Prognostic Score (mGPS) on PFS.

- The influence of standard biochemical parameters on therapeutic outcomes. Including:
  - adjusted calcium, bilirubin, alkaline phosphatase, aspartate transaminase, alanine transaminase and γ-glutamyl transferase;
  - GERCOR 2-stage prognostic model [53].

Multiple models will be investigated, seeking both univariate, multi-variate and parsimonious models of health outcomes. Longitudinal changes in markers as well as baseline values will be investigated. The results will be considered hypothesis-generating rather than confirmatory.

8.4 Sample Size

It is anticipated that the hazard ratio for NLR in the primary model will be larger than the hazard ratio (1.6) observed by Chua *et al.* [45] in their multivariate analysis. This is because the multivariate analysis adjusted for the presence of hypoalbuminemia, which is likely to be correlated with NLR, and would have reduced the apparent association. It is anticipated, therefore, that the true Hazard Ratio is at least 1.7 in favor of NLR≤5. Assuming the true incidence of NLR>5 is 30%, the median PFS is 10.5 months in patients with NLR≤5, and all patients are followed for 24 months, 150 patients has at least 80% power to detect a hazard ratio of this size.

9. DATA COLLECTION, MANAGEMENT AND QUALITY ASSURANCE

The overall procedures for quality assurance of clinical study data are described in the Roche Standard Operational Procedures.

Data for this study will be recorded via an Electronic Data Capture system EDC using electronic Case Report Forms (eCRF). In no case is the eCRF to be considered as source data for this trial.

Accurate and reliable data collection will be assured by verification and cross–check of the eCRFs against the investigator"s records by the Roche monitor for selected parameters as outlined in the Trial Monitoring Plan.

A comprehensive validation check program utilizing front-end checks in the eCRF and back-end checks in the Roche database will verify the data and discrepancies will be generated accordingly. These are transferred electronically to the eCRF at the site for resolution by the investigator.

Throughout the study the SMT will review data according to the SMT Data Review Plan as described in the Data Quality Plan.

9.1 Assignment of Preferred Terms and Original Terminology

For classification purposes, preferred terms will be assigned by the sponsor to the original terms entered on the eCRF, using the most up-to-date version of the Medical Dictionary for Regulatory Activities (MedDRA) terminology for adverse events and diseases and theWhoDrug Drug Terms and Procedures Dictionary for treatments and surgical and medical procedures..

PART II: ETHICS AND GENERAL STUDY ADMINISTRATION

10. ETHICAL ASPECTS

10.1 Local Regulations/Declaration of Helsinki

The investigator will ensure that this study is conducted in full conformance with the principles of the "Declaration of Helsinki" or with the laws and regulations of the country in which the research is conducted, whichever affords the greater protection to the individual. The study must fully adhere to the principles outlined in "Guideline for Good Clinical Practice" ICH Tripartite Guideline [January 1997] or with local law if it affords greater protection to the patient.

10.2 Informed Consent

It is the responsibility of the investigator to obtain signed informed consent from each patient prior to participating in this study after adequate explanation of the aims, methods, anticipated benefits, and potential hazards of the study.

The investigator or designee must also explain that the patients are completely free to refuse to enter the study or to withdraw from it at any time, for any reason.

If new safety information results in significant changes in the risk/benefit assessment, the consent form should be reviewed and updated if necessary. All patients (including those already being treated) should be informed of the new information, given a copy of the revised form and give their consent to continue in the study.

For the patient not qualified or incapable of giving legal consent, written consent must be obtained from the legally acceptable representative. In the case where both the patient and his/her legally acceptable representative are unable to read, an impartial witness should be present during the entire informed consent discussion. After the patient and representative have orally consented to participation in the trial, the witness" signature on the form will attest that the information in the consent form was accurately explained and understood.

In a life-threatening situation where a patient is unconscious or otherwise unable to communicate, the emergency is such that there is not enough time to obtain consent from the patient's legally acceptable representative, and there is no other or better treatment available, it is permissible to treat the patient under protocol with consent of the investigator, with appropriate documentation that the local IEC had approved the procedures used to enroll patients in such situations. In addition, the patient or his/her legally acceptable representative should be informed about the trial as soon as possible and consent to continue, giving written consent as described above.

10.3 Independent Ethics Committees (IEC)

This protocol and any accompanying material provided to the patient (such as patient information sheets or descriptions of the study used to obtain informed consent) as well as any advertising or compensation given to the patient, will be submitted by the investigator to an IEC.

An approval letter (specifying the protocol number and title) from the site IEC or state IEC as appropriate, must be obtained before study initiation specifying the date on which the committee met and granted the approval.

Any modifications made to the protocol after receipt of the IEC approval must also be submitted by the investigator to the site or state IEC as appropriate in accordance with local procedures and regulatory requirements.

Roche shall also provide investigators with Bevacizumab Six Monthly Safety Reports (SSRs) for submission to the site or state IEC as appropriate.

10.4 Role of the Science Advisory Committee (SAC)

A Scientific Advisory Committee (SAC) has been established for the ML25753 (ASCENT) trial and specific policies on the operation of the SAC have been documented in a Charter. The SAC will be led by a medically qualified Chairperson and other members who are physicians with experience in the treatment of metastatic colorectal cancer as well as a biostatistician.

The role of the ASCENT Trial Scientific Advisory Committee (SAC) is to provide scientific input into protocol development, monitor trial progress, ensure patient safety, develop recruitment strategies, and develop the publication strategy and planning for the trial.

The frequency of the SAC meetings will be approximately every 6 months or as deemed necessary.

10.5 Financial Disclosure

The investigator(s) will provide the sponsor with sufficient accurate financial information (PD35) to allow the sponsor to submit complete and accurate financial certification or disclosure statement to the appropriate regulatory authorities. The investigator is responsible to promptly update any information provided to the sponsor if relevant changes occur in the course of the investigation and for 1 year following the completion of the study.

11. CONDITIONS FOR MODIFYING THE PROTOCOL

All protocol modifications must be submitted to the appropriate site IEC or state IEC as appropriate, for approval in accordance with local requirements. Approval must be obtained before any changes can be implemented, except for changes necessary to eliminate an immediate hazard to trial patients, or when the change(s) involves only logistical or administrative aspects of the trial [e.g. change in monitor(s)], change of telephone number(s).

12. CONDITIONS FOR TERMINATING THE STUDY

Both the sponsor and the investigator reserve the right to terminate the study at any time. Should this be necessary, both parties will arrange the procedures on an individual study basis after review and consultation. In terminating the study, Roche and the investigator will assure that adequate consideration is given to the protection of the patient"s interests. The appropriate IEC and regulatory authorities should be informed accordingly.

13. STUDY DOCUMENTATION, eCRFS AND RECORD KEEPING

13.1 Investigator's Files / Retention of Documents

The investigator must maintain adequate and accurate records to enable the conduct of the study to be fully documented and the study data to be subsequently verified. These documents should be classified into two different separate categories: 1) Investigator's Study File, and 2) patient clinical source documents.

The Investigator's Study File will contain the protocol/amendments, and schedule of assessments, Independent Ethics Committee and regulatory approval with correspondence, sample informed consent, drug records, staff curriculum vitae and authorization forms and other appropriate documents/correspondence, etc. In addition at the end of the study the investigator will receive the patient data, which includes an audit trail containing a complete record of all changes to data, query resolution correspondence and reasons for changes, in human readable format on CD which also has to be kept with the Investigator"s Study File.

Subject clinical source documents (usually defined by the project in advance to record key efficacy/safety parameters independent of the eCRFs) would include patient hospital/clinic records, physician's and nurse's notes, appointment book, original laboratory reports, ECG, EEG, X-ray, pathology and special assessment reports, signed informed consent forms, consultant letters, and patient screening and enrollment logs. The investigator must keep the two categories of documents as described above (including the archival CD) on file for at least 15 years after completion or discontinuation of the study. After that period of time the documents may be destroyed, patient to local regulations.

Should the investigator wish to assign the study records to another party or move them to another location, Roche must be notified in advance.

If the investigator cannot guarantee this archiving requirement at the investigational site for any or all of the documents, special arrangements must be made between the investigator and Roche to store these in a sealed container(s) outside of the site so that they can be returned sealed to the investigator in case of a regulatory audit. Where source documents are required for the continued care of the patient, appropriate copies should be made for storing outside of the site.

ICH GCP guidelines require that investigators maintain information in the study patient"s records which corroborate data collected on the eCRF.

13.2 Source Documents and Background Data

The investigator shall supply the sponsor on request with any required background data from the study documentation or clinic records. This is particularly important when errors in data transcription are suspected. In case of special problems and/or governmental queries or requests for audit inspections, it is also necessary to have access to the complete study records, provided that patient confidentiality is protected.

13.3 Audits and Inspections

The investigator should understand that source documents for this trial should be made available to appropriately qualified personnel from the Roche Pharma Development Quality Assurance Unit or its designees, or to health authority inspectors after appropriate notification. The verification of the eCRF data must be by direct inspection of source documents.

13.4 Case Report Forms or Electronic Case Report Forms

Data for this study will be captured via an eCRF on a computer. The data collected in the source documents is entered onto the study eCRF. An audit trail will maintain a record of initial entries and changes made; reasons for change; time and date of entry; and user name of person authorizing entry or change.

For each patient enrolled, an eCRF must be completed and electronically signed by the principal investigator or authorized delegate from the study staff. This also applies to records for those patients who fail to complete the study (even during the screening period if an eCRF was initiated). If a patient withdraws from the study, the reason must be noted on the eCRF. If a patient is withdrawn from the study because of a treatment-limiting AE, thorough efforts should be made to clearly document the outcome.

The investigator should ensure the accuracy, completeness and timeliness of the data reported to the sponsor in the eCRFs and in all required reports.

14. MONITORING THE STUDY

It is understood that the responsible Roche monitor will contact and visit the investigator regularly and will be allowed, on request, to inspect the various records of the trial provided that patient confidentiality is maintained in accord with local requirements.

It will be the monitor's responsibility to inspect the eCRF at regular intervals throughout the study, to verify the adherence to the protocol and the completeness, consistency and accuracy of the data being entered on them. The monitor must verify that the patient correctly received the study drug as per protocol specific procedures. The monitor should have access to laboratory test reports and other patient records needed to verify the entries in the eCRF as required. The investigator (or designee) agrees to cooperate with the monitor to ensure that any problems detected in the course of these monitoring visits are resolved.

15. CONFIDENTIALITY OF TRIAL DOCUMENTS AND SUBJECT RECORDS

The investigator must assure that patients" anonymity will be maintained and that their identities are protected from unauthorized parties. On eCRFs or other documents submitted to the sponsor, patients should not be identified by their names, but by an identification code, a patient number. The investigator should keep a patient enrollment log showing codes, names and addresses.

16. CLINICAL STUDY REPORT (CSR)

A clinical study report will be written and distributed to Health Authorities as required by applicable regulatory requirements.

17. PUBLICATION OF DATA AND PROTECTION OF TRADE SECRETS

Roche will comply with the requirements for publication of study results.

The results of this study may be published or presented at scientific meetings. If this is foreseen, the investigator agrees to submit all manuscripts or abstracts to Roche prior to submission. This allows the sponsor to protect proprietary information and to provide comments based on information from other studies that may not yet be available to the investigator.

In accordance with standard editorial and ethical practice, Roche will generally support publication of multicenter trials only in their entirety and not as individual center data. In this case, a coordinating investigator will be designated by mutual agreement.

Authorship will be determined by mutual agreement and in line with International Committee of Medical Journal Editors (ICMJE) authorship requirements. Any formal publication of the study in which input of Roche personnel exceeded that of conventional monitoring will be considered as a joint publication by the investigator and the appropriate Roche personnel.

Any inventions and resulting patents, improvements and / or know- how originating from biomarker studies will become and remain the exclusive and unburdened property of Roche, except where agreed otherwise.

Data derived from biomarker study analysis on individual patients will not be provided to investigators, but may be specifically requested in writing to Roche. The aggregate results of any research conducted using biomarker specimens will be available in accordance with the effective Roche policy on study data publication.

Exploratory objectives pre-specified in the study protocol as well as exploratory analyses, defined as additional analyses not pre-specified in the original Statistical Analysis Plan (SAP) may not be published ahead of the complete study efficacy results.

17.1 Criteria for authorship

Authorship criteria for all publications of Roche-sponsored clinical trials are based on International Committee of Medical Journal Editors (ICMJE) "*Uniform Requirements for Manuscripts Submitted to Biomedical Journals*." Authorship credit can be granted only to those who make substantial contributions to the publication, including, but not limited to **ALL** of the following criteria:

a) Substantial contributions to conception and design, or acquisition of data, or analysis and interpretation of the data;
b) Drafting or revision of the abstract/manuscript for important intellectual content, and;
c) Approval of the final version to be published.

The authorship criteria apply equally to external investigators and to Roche employees. Roche is committed to disclosing any financial contributions made by the Company related to a clinical trial, any publication assistance provided by the Company to the author(s) (financial or otherwise), and any other disclosures required by the applicable journal or congress or local regulations.

17.2 ML25753 publication policies and rules

Authorship will be determined according to the pre-defined criteria for authorship outlined above in Section 17.1 and under the guidance of the Scientific Advisory Committee (SAC). The following guidelines will also apply:

- In addition to the ICMJE requirements, authorship, authorship will follow the guidelines of the target journal, where applicable. Especially considering the maximum number of authors permitted for the target journal;
- All peer-reviewed publication activities for ML25753 will be set forth in a publication plan to be reviewed by Roche and SAC annually.
- The Lead PI may elect to be first or last author on all study publications;
- Members of the Scientific Advisory Committee (SAC) in addition to the Lead PI will be authors on all study publications;
- The study biostatistician may be an author on all study publications;
- The Roche Medical/Clinical Lead will be an author on all publications on the basis that he/she was responsible for developing the study protocol;

18. APPENDICES

18.1 Proteomic Sub-study

Proteomic pattern diagnostics is a recent and potentially revolutionary approach for early disease detection, prognostication, and monitoring in oncology. The use of proteomic technologies might benefit biomarker discovery and treatment modalities and different profiles may be associated with varying responses to therapeutics and other clinically relevant parameters and may also serve as prediction for treatment outcome.

The primary objective of ML25753 is to study the effect of the host inflammatory response (measured by NLR) on the clinical outcome of patients with mCRC treated with bevacizumab. The liver is a key player in the initiation and maintenance of systemic inflammatory response. Hepatocytes are stimulated to synthesize, and release into the systemic circulation, a variety of acute phase proteins that initiate, sustain or curtail the systemic inflammatory response. Measurement of acute phase proteins in plasma is a potentially useful means by which to monitor the systemic inflammatory response. Using Multiple Reaction Monitoring (MRM) proteomics technology, [redacted] [1] have identified changes in 32 high/medium abundance liver-derived plasma proteins (see Table 6) which may correlate with treatment response and toxicity in mCRC patients treated with chemotherapy[2]. Prospective studies with larger patient numbers are needed to determine if alterations in acute phase plasma protein profiles can offer prognostic or predictive value in mCRC when patients are treated with chemotherapy and bevacizumab. If as predicted NLR is shown to be highly prognostic, it will be important to determine the biological underpinnings of the NLR and which acute-phase proteins are key drivers of the inflammatory response.

Table 6: ***Candidate liver-derived plasma proteins to be studied.***

Alpha-1-antichymotrypsin	Alpha-1-antitrypsin	Alpha-1-acid glycoprotein 1	Alpha-2-macroglobulin	Alpha-1-microglobulin
Angiotensinogen	Antithrombin III	ApoLipoprotein A1	ApoLipoprotein A4	ApoLipoprotein B
ApoLipoprotein C3	Corticosteroid-binding globulin	Complement factor H	Ceruloplasmin	Clusterin
Complement C3	Complement C4-A	C-reactive protein	Fibrinogen A	Fibrinogen B

1 [redacted] personal communication
2 McKay et al. The development of multiple reaction monitoring assays for liver-derived plasma proteins. Proteomics Clin. Appl. 2007;1:1570–1581.

Fibrinogen C	Haptoglobin A	Haptoglobin B	Hemopexin	Kininogen
Paraoxonase/arylesterase1	Serum Amyloid A	Prothrombin	Serotransferrin	Transthyretin (Prealbumin)
Vitamin D binding protein	Vitronectin			

Objectives

The objective of the proteomic sub-study is to assess the value of acute-phase liver derived plasma proteins, measured by MRM, as prognostic or predictive biomarkers in patients with mCRC undergoing treatment with bevacizumab and fluoropyrimidine-based chemotherapy. Specifically the sub-study will:

- Study the relationship between NLR and liver-derived plasma proteins;
- Correlate baseline and changes in plasma protein levels from mCRC patient samples with treatment-related toxicity, treatment response, tumor progression and overall survival.

Sampling requirements

Plasma blood sampling is a mandatory criterion for inclusion in the ML25753. An initial pilot study will be undertaken using plasma samples from the first 50 patients to enroll in ML25753. Timing and frequency of the sampling will be in parallel with the blood draws as scheduled for the main study or as per institutional standards of care (Table 7).

Table 7: *Plasma sampling schedule*

Sample	Comments
**1. Pre-treatment baseline**	• ≤ 7 days prior to start of study treatment
**2. Day 3, Cycle 1 of Phase A treatment**	• This sample collection does not coincide with other study laboratory assessments and therefore requires an additional blood draw for the patient
**3. Day 14/21 of Phase A treatment**	• Taken prior to cycle 2<br>• Must be taken before drug administration<br>• To coincide with the chemotherapy cycle
**4. End of Phase A treatment**	• To be taken at the EoT safety follow-up visit at the end of Phase A treatment
**5. End of Phase B treatment**	• To be taken at the EoT safety follow-up visit at the end of Phase B treatment

Plasma samples should be collected and processed as follows:

1) Blood (1 x 6 mL) is drawn into a spray dried K_2 EDTA containing collection tube without gel separator. Ensure free flow with mild aspiration to avoid hemolysis. After filling the collection tube, each tube has to be inverted eight times to facilitate the mixing of blood and anticoagulant;

2) Samples should be stored at room temperature and centrifuged within 30 minutes of collection;

3) Within 30 minutes of blood collection, place the blood tube into the centrifuge and spin at 2500g at 20°C for 20mins. Label a 5 mL transfer tube with the appropriate transfer tube label;

4) Immediately after centrifugation, carefully transfer the plasma into the labeled transfer tube using a plastic pipette, being careful not to aspirate the interface containing the platelets (leave sufficient supernatant above sediment to avoid contamination with cells);

5) Once the plasma is transferred to the transfer tube, discard the blood collection tube;

6) Aliquot the plasma sample into labeled storage tubes (divide the plasma equally between the three storage tubes, approx. 1ml per tube). Discard the transfer tube;

7) Label the storage tubes with the appropriate labels provided by Roche;

8) Store the three storage tubes upright in a -70ºC freezer until shipment to [redacted];

9) If only a -20°C is available then samples must be shipped to [redacted]/storage facility within four weeks of being drawn.

Should an investigator wish to undertake any further research with plasma samples collected in study ML25753, a research proposal must be submitted to Roche and the SAC and be approved prior to commencement of any research.

18.2 ICH Guidelines for Clinical Safety Data Management, Definitions and Standards for Expedited Reporting, Topic E2

A serious adverse event is any experience that suggests a significant hazard, contraindication, side effect or precaution. It is any AE that at any dose fulfills at least one of the following criteria:

1. is fatal; [results in death] [NOTE: death is an outcome, not an event]

2. is Life-Threatening [NOTE: the term "Life-Threatening" refers to an event in which the patient was at immediate risk of death at the time of the event; it does not refer to an event which could hypothetically have caused a death had it been more severe].

3. required in-patient hospitalization or prolongation of existing hospitalization;

4. results in persistent or significant disability/incapacity;

5. is a congenital anomaly/birth defect;

6. is medically significant or requires intervention to prevent one or other of the outcomes listed above

Medical and scientific judgment should be exercised in deciding whether expedited reporting to the sponsor is appropriate in other situations, such as important medical events that may not be immediately life-threatening or result in death or hospitalization but may jeopardize the patient or may require intervention to prevent one of the outcomes listed in the definitions above. These situations should also usually be considered serious.

Examples of such events are intensive treatment in an emergency room or at home for allergic bronchospasm; blood dyscrasias or convulsions that do not result in hospitalization; or development of drug dependency or drug abuse.

An unexpected AE is one, the nature or severity of which is not consistent with the applicable product information.

Causality is initially assessed by the investigator. For Serious Adverse Events, possible causes of the event **are** indicated by selecting one or more options. (Check all that apply)

7. Pre-existing/Underlying disease **-** specify

8. Study treatment – specify the drug(s) related to the event

9. Other treatment (concomitant or previous) – specify

10. Protocol-related procedure

11. Other (e.g. accident, new or intercurrent illness) - specify

The term severe is a measure of intensity, thus a severe AE is not necessarily serious. For example, nausea of several hours' duration may be rated as severe, but may not be clinically serious.

A serious adverse event occurring during the study or which comes to the attention of the investigator within 15 days after stopping the treatment or during the protocol-defined follow-up period, if this is longer, whether considered treatment-related or not, must be reported. In addition, a serious adverse event that occurs after this time, if considered related to test "drug", should be reported.

Such preliminary reports will be followed by detailed descriptions later which will include copies of hospital case reports, autopsy reports and other documents when requested and applicable.

For serious adverse events, the following must be assessed and recorded on the AEs page of the eCRF: intensity, relationship to test substance, action taken, and outcome to date.

The investigator must notify the Ethics Review Committee/Institutional Review Board of a serious adverse event in writing as soon as is practical and in accordance with international and local laws and regulations.

ROCHE LOCAL COUNTRY CONTACT for SAEs: Local Monitor

See *Protocol Administrative and Contact Information & List of Investigators Form, [gcp_for000227]*, for details of administrative and contact information.

ROCHE HEADQUARTERS CONTACT for SAEs and other medical emergencies: Clinical Operations/Clinical Science

See *Protocol Administrative and Contact Information & List of Investigators form, [gcp_for000227]*, for details of administrative and contact information.

18.3 WHO Performance status

Grade	Explanation of activity
0	Fully active, able to carry on all pre-disease performance without restriction
1	Restricted in physically strenuous activity but ambulatory and able to carry out work of a light or sedentary nature, e.g., light house work, office work
2	Ambulatory and capable of all selfcare but unable to carry out any work activities. Up and about more than 50% of waking hours
3	Capable of only limited selfcare, confined to bed or chair more than 50% of waking hours
4	Completely disabled. Cannot carry on any selfcare. Totally confined to bed or chair
5	Dead

18.4 EQ-5D

By placing a tick in one box in each group below, please indicate which statements best describe your own health state today.

Mobility

I have no problems in walking about ☐

I have some problems in walking about ☐

I am confined to bed ☐

Self-Care

I have no problems with self-care ☐

I have some problems washing or dressing myself ☐

I am unable to wash or dress myself ☐

Usual Activities (*e.g. work, study, housework, family or leisure activities)*

I have no problems with performing my usual activities ☐

I have some problems with performing my usual activities ☐

I am unable to perform my usual activities ☐

Pain/Discomfort

I have no pain or discomfort ☐

I have moderate pain or discomfort ☐

I have extreme pain or discomfort ☐

Anxiety/Depression

I am not anxious or depressed ☐

I am moderately anxious or depressed ☐

I am extremely anxious or depressed ☐

Best imaginable health state

To help people say how good or bad a health state is, we have drawn a scale (rather like a thermometer) on which the best state you can imagine is marked 100 and the worst state you can imagine is marked 0.

We would like you to indicate on this scale how good or bad your own health is today, in your opinion. Please do this by drawing a line from the box below to whichever point on the scale indicates how good or bad your health state is today.

Your own health state today

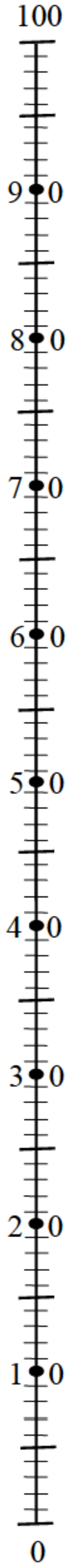

Worst imaginable health state

18.5 AQoL 8-D

The AQoL questionnaire can be found at
http://www.aqol.com.au/aqolquestionnaires.html

18.6 FACT-C (Version 4)

Below is a list of statements that other people with your illness have said are important. **Please circle or mark one number per line to indicate your response as it applies to the <u>past 7 days</u>.**

	**PHYSICAL WELL-BEING**	**Not at all**	**A little bit**	**Some-what**	**Quite a bit**	**Very much**
GP1	I have a lack of energy	0	1	2	3	4
GP2	I have nausea	0	1	2	3	4
GP3	Because of my physical condition, I have trouble meeting the needs of my family	0	1	2	3	4
GP4	I have pain	0	1	2	3	4
GP5	I am bothered by side effects of treatment	0	1	2	3	4
GP6	I feel ill	0	1	2	3	4
GP7	I am forced to spend time in bed	0	1	2	3	4

Please circle or mark one number per line to indicate your response as it applies to the <u>past 7 days</u>.

SOCIAL/FAMILY WELL-BEING

		Not at all	A little bit	Some-what	Quite a bit	Very much
GS1	I feel close to my friends	0	1	2	3	4
GS2	I get emotional support from my family	0	1	2	3	4
GS3	I get support from my friends	0	1	2	3	4
GS4	My family has accepted my illness	0	1	2	3	4
GS5	I am satisfied with family communication about my illness	0	1	2	3	4
GS6	I feel close to my partner (or the person who is my main support)	0	1	2	3	4
Q1	*Regardless of your current level of sexual activity, please answer the following question. If you prefer not to answer it, please mark this box* ☐ *and go to the next section.*					
GS7	I am satisfied with my sex life	0	1	2	3	4

EMOTIONAL WELL-BEING

		Not at all	A little bit	Some-what	Quite a bit	Very much
GE1	I feel sad	0	1	2	3	4
GE2	I am satisfied with how I am coping with my illness	0	1	2	3	4
GE3	I am losing hope in the fight against my illness	0	1	2	3	4
GE4	I feel nervous	0	1	2	3	4
GE5	I worry about dying	0	1	2	3	4
GE6	I worry that my condition will get worse	0	1	2	3	4

FUNCTIONAL WELL-BEING

		Not at all	A little bit	Some-what	Quite a bit	Very much
GF1	I am able to work (include work at home)	0	1	2	3	4
GF2	My work (include work at home) is fulfilling	0	1	2	3	4
GF3	I am able to enjoy life	0	1	2	3	4
GF4	I have accepted my illness	0	1	2	3	4
GF5	I am sleeping well	0	1	2	3	4
GF6	I am enjoying the things I usually do for fun	0	1	2	3	4
GF7	I am content with the quality of my life right now	0	1	2	3	4

ADDITIONAL CONCERNS

		Not at all	A little bit	Some-what	Quite a bit	Very much
C1	I have swelling or cramps in my stomach area	0	1	2	3	4
C2	I am losing weight	0	1	2	3	4
C3	I have control of my bowels	0	1	2	3	4
C4	I can digest my food well	0	1	2	3	4
C5	I have diarrhea (diarrhoea)	0	1	2	3	4
C6	I have a good appetite	0	1	2	3	4
C7	I like the appearance of my body	0	1	2	3	4
O2	Do you have an ostomy appliance? (Mark one box)	☐ No		or	☐ Yes	
	If yes, please answer the next two items:					
C8	I am embarrassed by my ostomy appliance	0	1	2	3	4
C9	Caring for my ostomy appliance is difficult	0	1	2	3	4

18.7 NYHA Classification of Heart failure

Functional capacity (four classes)

Class I: No limitation of physical activity

Ordinary physical activity does not cause undue fatigue, palpitation or dyspnea

Class II: Slight limitation of physical activity

Comfortable at rest, but ordinary physical activity results in fatigue, palpitation or dyspnea

Class III: Marked limitation of physical activity

Comfortable at rest, but less than ordinary activity causes fatigue, palpitation or dyspnea

Class IV: Unable to carry on any physical activity without discomfort. Symptoms of cardiac insufficiency may be present even at rest. If any physical activity is undertaken, discomfort is increased

18.8 Common Terminology Criteria for Adverse Events v4.0

The Common Terminology Criteria for Adverse Events v4.0, instituted June 14, 2010, is available at: http://evs.nci.nih.gov/ftp1/CTCAE/About.html

18.9 Suggested treatment algorithm for the management of bevacizumab-induced hypertension

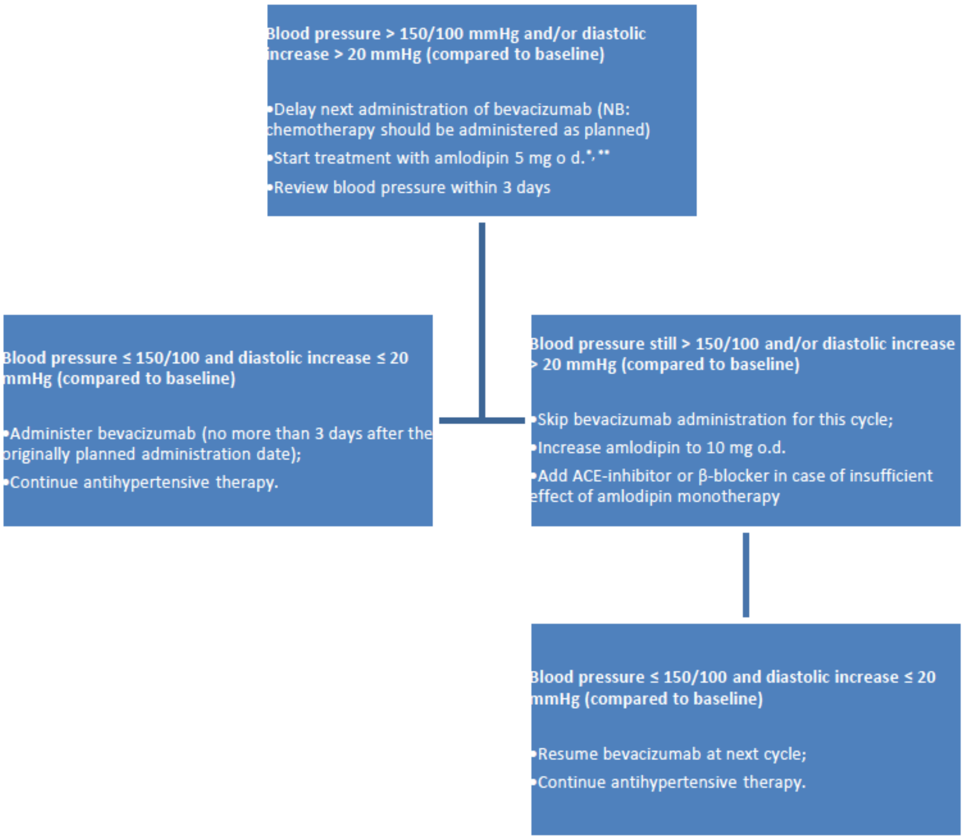

* This algorithm is a recommendation, final choice of antihypertensive drug(s) is at the discretion of the treating physician

** ACE-inhibitors or angiotensin 2 receptor antagonists may be considered as initial therapy in patients with proteinuria, chronic kidney disease risks or metabolic syndrome (Izzedine *et al*, 2009)

18.10 Pairing antihypertensive agents

When a second antihypertensive agent is required to control the blood pressure, the agent should be selected based on: the patient"s capacity to tolerate either agent individually, the complementarity of mechanism of action, evidence of better antihypertensive effect of the combination than either individual agent, and favorable tolerability of the combination. The following combinations have been recommended by the European Societies of Hypertension and Cardiology[3] for treatment of chronic hypertension in the general population:

- Calcium antagonist and angiotensin converting enzyme (ACE) inhibitor
- Calcium antagonist and angiotensin receptor antagonist
- Calcium antagonist (dihydropiridine) and β-blocker
- Calcium antagonist and thiazide diuretic
- Thiazide diuretic and ACE inhibitor
- Thiazide diuretic and angiotensin receptor antagonist

[3] Blood Press. 2007;16(3):135-232.

18.11 Capecitabine dose calculation according to body surface area (BSA)

500 mg tablet only

Where 100% dose level is twice-daily 1000 mg/m^2 (total daily dose of 2000 mg/m^2)

100% Dose Level = twice-daily 1000 mg/m^2		Number of 500 mg tablets to be taken in the	
Surface Area (m^2)	Average Dose per Administration (mg)	Morning	Evening
≤ 1.12	1000	2	2
1.13 – 1.37	1250	2	3
1.38 – 1.62	1500	3	3
1.63 – 1.87	1750	3	4
≥ 1.88	2000	4	4

75% Dose Level		Number of 500 mg tablets to be taken in the	
Surface Area (m^2)	Average Dose per Administration (mg)	Morning	Evening
≤ 1.12	750	1	2
1.13 – 1.37	1000	2	2
1.38 – 1.87	1250	2	3
≥ 1.88	1500	3	3

50% Dose Level		Number of 500 mg tablets to be taken in the	
Surface Area (m^2)	Average Dose per Administration (mg)	Morning	Evening
≤ 1.12	500	1	1
1.13 – 1.87	750	1	2
≥ 1.88	1000	2	2

18.12 General Guidance for Dose Modifications of Irinotecan/5-FU

* Grade 4 -Contin
best interest of th

18.13 Modified Charlson Comorbidity Index

Below is a screen display of the Modified Charlson Comorbidity Index that will be provided to sites for use throughout the study.

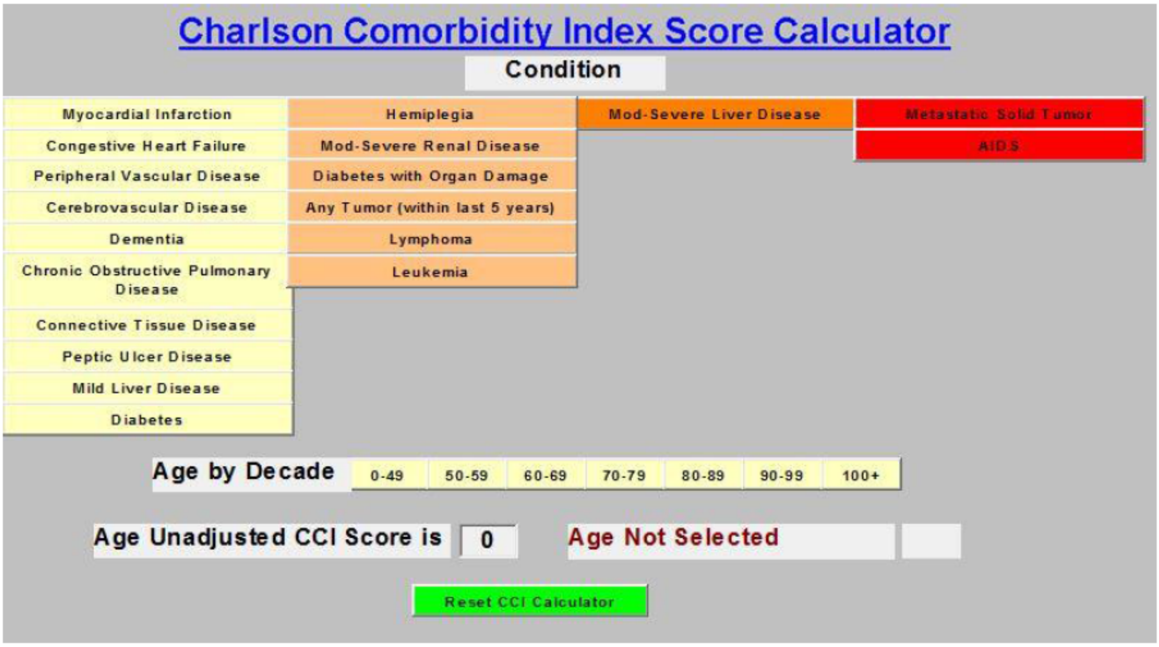

Instructions on how to Compute Charlson Comorbidity Index (CCI) Score:

1. To calculate a CCI score, select any of the applicable conditions.
2. All selected conditions will then be displayed in a lighter shade within the table.
3. Corrections can be made by unselecting conditions, which will remove their weighted value from the score.
4. The CCI score can then be totaled or an age-modified score can be determined by selecting any one of the applicable "age by decade" groups.
5. Scores totalled by selecting an age group without selecting comorbidity will result in no value for either total and you will be prompted to "Reset & Select Condition."
6. To reset the program, the "Reset CCI Calculator" button can be selected.
7. Scores totalled without age modification will appear in the "Age Unadjusted CCI Score" total and no value will appear in the "Age Adjusted Score" total.

*Note the upper limit scores for this calculator are 37 for age "unadjusted" and 43 for "age adjusted." CCI scores >8-10 have not received extensive evaluation.

19. REFERENCES

1. Van Cutsem E, Nordlinger B, Cervantes A, On behalf of the EGWG: **Advanced colorectal cancer: ESMO Clinical Practice Guidelines for treatment**. *Ann Oncol* 2010, **21**(suppl 5):v93-v97.

2. Kopetz S, Chang GJ, Overman MJ, Eng C, Sargent DJ, Larson DW, Grothey A, Vauthey J-N, Nagorney DM, McWilliams RR: **Improved Survival in Metastatic Colorectal Cancer Is Associated With Adoption of Hepatic Resection and Improved Chemotherapy**. *Journal of Clinical Oncology* 2009, **27**(22):3677-3683.

3. Tournigand C, AndrÃ© T, Achille E, Lledo Gr, Flesh M, Mery-Mignard D, Quinaux E, Couteau C, Buyse M, Ganem Gr *et al*: **FOLFIRI Followed by FOLFOX6 or the Reverse Sequence in Advanced Colorectal Cancer: A Randomized GERCOR Study**. *Journal of Clinical Oncology* 2004, **22**(2):229-237.

4. Grothey A, Sargent D, Goldberg RM, Schmoll H-J: **Survival of Patients With Advanced Colorectal Cancer Improves With the Availability of Fluorouracil-Leucovorin, Irinotecan, and Oxaliplatin in the Course of Treatment**. *Journal of Clinical Oncology* 2004, **22**(7):1209-1214.

5. Hicklin DJ, Ellis LM: **Role of the Vascular Endothelial Growth Factor Pathway in Tumor Growth and Angiogenesis**. *Journal of Clinical Oncology* 2005, **23**(5):1011-1027.

6. De Bock K, Cauwenberghs S, Carmeliet P: **Vessel abnormalization: another hallmark of cancer?: Molecular mechanisms and therapeutic implications**. *Current Opinion in Genetics & Development* 2011, **21**(1):73-79.

7. Bagri A, Berry L, Gunter B, Singh M, Kasman I, Damico LA, Xiang H, Schmidt M, Fuh G, Hollister B *et al*: **Effects of Anti-VEGF Treatment Duration on Tumor Growth, Tumor Regrowth, and Treatment Efficacy**. *Clin Cancer Res* 2010, **16**(15):3887-3900.

8. Welch S: **Bevacizumab combined with chemotherapy for patients with advanced colorectal cancer: a systematic review**. *Ann Oncol* 2010, **21**:1152-1162.

9. Galfrascoli E, Piva S, Cinquini M, Rossi A, La Verde N, Bramati A, Moretti A, Manazza A, Damia G, Torri V *et al*: **Risk/benefit profile of bevacizumab in metastatic colon cancer: A systematic review and meta-analysis**. *Digestive and Liver Disease*, 2011 **43**(4):286-94

10. Wagner A, Arnold D, Grothey A, Haerting J, Unverzagt S: **Anti-angiogenic therapies for metastatic colorectal cancer**. *Cochrane Database of Systematic Reviews* 2009(3):1-74.

11. Giantonio BJ: **Bevacizumab in combination with oxaliplatin, fluorouracil, and leucovorin (FOLFOX4) for previously treated metastatic colorectal cancer: results from the Eastern Cooperative Oncology Group Study E3200**. *J Clin Oncol* 2007, **25**:1539-1544.

12. Saltz LB: **Bevacizumab in combination with oxaliplatin-based chemotherapy as first-line therapy in metastatic colorectal cancer: a randomized phase III study**. *J Clin Oncol* 2008, **26**:2013-2019.

13. Hurwitz H, Fehrenbacher L, Novotny W, Cartwright T, Hainsworth J, Heim W, Berlin J, Baron A, Griffing S, Holmgren E *et al*: **Bevacizumab plus Irinotecan, Fluorouracil, and Leucovorin for Metastatic Colorectal Cancer**. *N Engl J Med* 2004, **350**(23):2335-2342.

14. Tebbutt NC: **Capecitabine, bevacizumab and mitomycin in first-line treatment of metastatic colorectal cancer: results of the Australasian Gastrointestinal Trials Group randomized, phase III MAX study**. *J Clin Oncol* 2010, **28**:3191-3198.

15. Hess GP, Wang PF, Quach D, Barber B, Zhao Z: **Systemic Therapy for Metastatic Colorectal Cancer: Patterns of Chemotherapy and Biologic Therapy Use in US Medical Oncology Practice**. *J Oncol Pract* 2010, **6**(6):301-307.

16. Grothey A, Sugrue MM, Purdie DM, Dong W, Sargent D, Hedrick E, Kozloff M: **Bevacizumab Beyond First Progression Is Associated With Prolonged Overall Survival in Metastatic Colorectal Cancer: Results From a Large Observational Cohort Study (BRiTE)**. *Journal of Clinical Oncology* 2008, **26**(33):5326-5334.

17. Bennouna J, Sastre J, Arnold D, *et al.* **Continuation of bevacizumab after first progression in metastatic colorectal cancer (ML18147): a randomized phase 3 trial**. *Lancet Oncol 2012*. **E-pub ahead of print** (published online November 16, 2012).

18. Masi G, Loupakis F, Salvatore L, Fornaro, Cremolini C, Schirripa M, Granetto C, Fea E, Antonuzzo L, Giommoni E, *et al*: **A randomized study evaluating the continuation of bevacizumab beyond progression in metastatic colorectal cancer patients who received bevacizumab as part of first-line treatment:**

results of the BEBYP trial by the Gruppo Oncologico Nord Ovest (GONO) *European Society for Medical Oncology 37th Congress - ESMO 2012 (LBA17)*

19. Hapani S, Chu D, Wu S: **Risk of gastrointestinal perforation in patients with cancer treated with bevacizumab: a meta-analysis**. *The Lancet Oncology* 2009, **10**(6):559-568.

20. Cohn A, Bekaii-Saab T, Bendell J, Hurwitz H, Kozloff M, Roach N, Tezcan H, Feng S, Sing A, Grothey A *et al*: **Clinical outcomes in bevacizumab (BV)-treated patients (pts) with metastatic colorectal cancer (mCRC): Results from ARIES observational cohort study (OCS) and confirmation of BRiTE data on BV beyond progression (BBP)**. *Journal of Clinical Oncology* 2010, **28**(15s).

21. Van Cutsem E, Rivera F, Berry S, Kretzschmar A, Michael M, DiBartolomeo M, Mazier MA, Canon JL, Georgoulias V, Peeters M *et al*: **Safety and efficacy of first-line bevacizumab with FOLFOX, XELOX, FOLFIRI and fluoropyrimidines in metastatic colorectal cancer: the BEAT study**. *Ann Oncol* 2009, **20**(11):1842-1847.

22. Chau I, Cunningham D: **Bevacizumab-associated gastrointestinal perforation**. *The Lancet Oncology* 2009, **10**(6):534-536.

23. Saif M, Elfiky A, Salem R: **Gastrointestinal Perforation Due to Bevacizumab in Colorectal Cancer**. *Annals of Surgical Oncology* 2007, **14**(6):1860-1869.

24. Sugrue M, Kozloff M, Hainsworth J, Badarinath S, Cohn A, Flynn P, Steis R, Dong W, Sarkar S, Grothey A: **Risk factors for gastrointestinal perforations in patients with metastatic colorectal cancer receiving bevacizumab plus chemotherapy**. *Journal of Clinical Oncology* 2006, **24**(18_suppl):3535-.

25. Small AJ, Coelho-Prabhu N, Baron TH: **Endoscopic placement of self-expandable metal stents for malignant colonic obstruction: long-term outcomes and complication factors**. *Gastrointestinal Endoscopy* 2010, **71**(3):560-572.

26. McCahill LE, Yothers GA, Sharif S, Petrelli NJ, Lopa S, O'Connell MJ, Wolmark N: **A phase II trial of 5-fluorouracil, leucovorin, and oxaliplatin (mFOLFOX6) chemotherapy plus bevacizumab for patients with unresectable stage IV colon cancer and a synchronous asymptomatic primary tumor: Results of NSABP C -10.** *Journal of Clinical Oncology* 2010, **28**(7s).

27. Poultsides GA, Servais EL, Saltz LB, Patil S, Kemeny NE, Guillem JG, Weiser M, Temple LKF, Wong WD, Paty PB: **Outcome of Primary Tumor in Patients With Synchronous Stage IV Colorectal Cancer Receiving Combination Chemotherapy Without Surgery As Initial Treatment**. *Journal of Clinical Oncology* 2009, **27**(20):3379-3384.

28. Field K, Desai J, Tie J, Kosmider S, Harold M, Gibbs P: **INITIAL TREATMENT OF METASTATIC COLORECTAL CANCER: USING PROSPECTIVE DATA TO UNDERSTAND ROUTINE CLINICAL PRACTICE**. *Asia–Pac J Clin Oncol : 100–147* 2010, **6**(Suppl. 3):144.

29. Buyse M, Sargent DJ, Grothey A, Matheson A, de Gramont A: **Biomarkers and surrogate end points - the challenge of statistical validation**. *Nat Rev Clin Oncol* 2010, **7**(6):309-317.

30. Atkinson AJ, Colburn WA, DeGruttola VG, DeMets DL, Downing GJ, Hoth DF, Oates JA, Peck CC, Schooley RT, Spilker BA *et al*: **Biomarkers and surrogate endpoints: Preferred definitions and conceptual framework**. *Clin Pharmacol Ther* 2001, **69**(3):89-95.

31. Jubb AM, Hurwitz HI, Bai W, Holmgren EB, Tobin P, Guerrero AS, Kabbinavar F, Holden SN, Novotny WF, Frantz GD *et al*: **Impact of Vascular Endothelial Growth Factor-A Expression, Thrombospondin-2 Expression, and Microvessel Density on the Treatment Effect of Bevacizumab in Metastatic Colorectal Cancer**. *2006* 2006, **24**(2):217-227.

32. Jubb AM, Harris AL: **Biomarkers to predict the clinical efficacy of bevacizumab in cancer**. *The Lancet Oncology* 2010, **11**(12):1172-1183.

33. Price TJ, Hardingham JE, Lee CK, Weickhardt A, Townsend AR, Wrin JW, Chua A, Shivasami A, Cummins MM, Murone C *et al*: **Impact of KRAS and BRAF Gene Mutation Status on Outcomes From the Phase III AGITG MAX Trial of Capecitabine Alone or in Combination With Bevacizumab and Mitomycin in Advanced Colorectal Cancer**. *Journal of Clinical Oncology* 2011, **29**(19):2675-82).

34. Hanahan D, Weinberg Robert A: **Hallmarks of Cancer: The Next Generation**. *Cell* 2011, **144**(5):646-674.

35. Grivennikov SI, Greten FR, Karin M: **Immunity, Inflammation, and Cancer**. *Cell* 2010, **140**(6):883-899.

36. Moore MM, Chua W, Charles KA, Clarke SJ: **Inflammation and Cancer: Causes and Consequences**. *Clin Pharmacol Ther* 2010, **87**(4):504-508.

37. Colotta F, Allavena P, Sica A, Garlanda C, Mantovani A: **Cancer-related inflammation, the seventh hallmark of cancer: links to genetic instability**. *Carcinogenesis* 2009, **7**:1073-1081.

38. Jarnicki A, Putoczki T, Ernst M: **Stat3: linking inflammation to epithelial cancer - more than a "gut" feeling?** *Cell Division* 2010, **5**(1):14.

39. Bergers G, Hanahan D: **Modes of resistance to anti-angiogenic therapy**. *Nat Rev Cancer* 2008, **8**(8):592-603.

40. Ellis LM, Hicklin DJ: **Pathways Mediating Resistance to Vascular Endothelial Growth Factor-Targeted Therapy**. *Clin Cancer Res* 2008, **14**(20):6371-6375.

41. Shojaei F, Wu X, Malik AK, Zhong C, Baldwin ME, Schanz S, Fuh G, Gerber H-P, Ferrara N: **Tumor refractoriness to anti-VEGF treatment is mediated by CD11b+Gr1+ myeloid cells**. *Nat Biotech* 2007, **25**(8):911-920.

42. Kopetz S, Hoff PM, Morris JS, Wolff RA, Eng C, Glover KY, Adinin R, Overman MJ, Valero V, Wen S *et al*: **Phase II Trial of Infusional Fluorouracil, Irinotecan, and Bevacizumab for Metastatic Colorectal Cancer: Efficacy and Circulating Angiogenic Biomarkers Associated With Therapeutic Resistance**. *Journal of Clinical Oncology* 2010, **28**(3):453-459.

43. Gabay C, Kushner I: **Acute-Phase Proteins and Other Systemic Responses to Inflammation**. *N Engl J Med* 1999, **340**(6):448-454.

44. Kishi Y, Kopetz S, Chun Y, Palavecino M, Abdalla E, Vauthey JN: **Blood neutrophil-to-lymphocyte ratio predicts survival in patients with colorectal liver metastases treated with systemic chemotherapy**. *Ann Surg Oncol* 2009, **16**:614-622.

45. Chua W, Charles KA, Baracos VE, Clarke SJ: **Neutrophil/lymphocyte ratio predicts chemotherapy outcomes in patients with advanced colorectal cancer**. *Br J Cancer* 2011, **104**(8):1288-1295.

46. Leighl NB, Bennouna J, Yi J, Moore N, Hambleton J, Hurwitz H: **Bleeding events in bevacizumab-treated cancer patients who received full-dose anticoagulation and remained on study**. *Br J Cancer* 2011.

47. Grothey A, Nikcevich DA, Sloan JA, Kugler JW, Silberstein PT, Dentchev T, Wender DB, Novotny PJ, Chitaley U, Alberts SR *et al*: **Intravenous Calcium and Magnesium for Oxaliplatin-Induced Sensory Neurotoxicity in Adjuvant Colon Cancer: NCCTG N04C7**. *Journal of Clinical Oncology* 2011, **29**(4):421-427.

48. Gamelin L, Boisdron-Celle M, Delva R, Guerin-Meyer Vr, Ifrah N, Morel A, Gamelin E: **Prevention of Oxaliplatin-Related Neurotoxicity by Calcium and Magnesium Infusions**. *Clin Cancer Res* 2004, **10**(12):4055-4061.

49. Knijn N, Tol J, Koopman M, Werter MJBP, Imholz ALT, Valster FAA, Mol L, Vincent AD, Teerenstra S, Punt CJA: **The effect of prophylactic calcium and magnesium infusions on the incidence of neurotoxicity and clinical outcome of oxaliplatin-based systemic treatment in advanced colorectal cancer patients**. *European Journal of Cancer* 2011, **47**(3):369-374.

50. Chun YS, Vauthey J-N, Boonsirikamchai P, Maru DM, Kopetz S, Palavecino M, Curley SA, Abdalla EK, Kaur H, Charnsangavej C *et al*: **Association of Computed Tomography Morphologic Criteria With Pathologic Response and Survival in Patients Treated With Bevacizumab for Colorectal Liver Metastases**. *JAMA* 2009, **302**(21):2338-2344.

51. Grothey A, Hedrick EE, Mass RD, Sarkar S, Suzuki S, Ramanathan RK, Hurwitz HI, Goldberg RM, Sargent DJ: **Response-Independent Survival Benefit in Metastatic Colorectal Cancer: A Comparative Analysis of N9741 and AVF2107**. *Journal of Clinical Oncology* 2008, **26**(2):183-189.

52. Desar IME, van Herpen CML, van Laarhoven HWM, Barentsz JO, Oyen WJG, van der Graaf WTA: **Beyond RECIST: Molecular and functional imaging techniques for evaluation of response to targeted therapy**. *Cancer Treatment Reviews* 2009, **35**(4):309-321.

53. Chibaudel B, Tournigand C, Bonnetain F, Andre T, Lledo G, Maindrault-Goebel F, Larsen AK, Louvet C, de Gramont A: **Handy prognostic model in patients with oxaliplatin-based or irinotecan-based first-line chemotherapy for metastatic colorectal cancer**. *Journal of Clinical Oncology* 2010, **28**(15_suppl):3577
