# Supplementary material for: The prognostic role of inflammatory markers in patients with metastatic colorectal cancer treated with bevacizumab: A translational study [ASCENT]
Source: PLoS One. 2020 Mar 6;15(3):e0229900. doi: 10.1371/journal.pone.0229900 (PMC7059922; doi:10.1371/journal.pone.0229900)
Supplement: S1 Table — (DOCX) [file pone.0229900.s003.docx]

| **S1 Table: Predicted PFS probabilities from the Primary Model (full analysis set)** | | | | | | | | | |
| --- | --- | --- | --- | --- | --- | --- | --- | --- | --- |
| Baseline Characteristics | | | | | Predicted PFS probability | | | | |
| NLR | WHO Performance Status | Metastatic Disease of the liver | Number of site of Metastatic Disease | Presence of Metastatic Disease in the liver with no other sites involved | Hazard Ratio | 6 months | 1 yr | 1.5 yr | 2 yr |
| <=5 | 0 | N | 0-3 | N | Reference | 84.3% | 56.1% | 43.7% | 35.1% |
| <=5 | 0 | N | 0-3 | Y | 1.0 | 84.7% | 57.1% | 44.7% | 36.2% |
| <=5 | 0 | N | >3 | N | 1.0 | 83.6% | 54.5% | 41.9% | 33.3% |
| <=5 | 0 | N | >3 | Y | 1.0 | 84.0% | 55.5% | 43.0% | 34.4% |
| <=5 | 0 | Y | 0-3 | N | 1.5 | 77.3% | 41.9% | 28.7% | 20.7% |
| <=5 | 0 | Y | 0-3 | Y | 1.5 | 77.9% | 43.0% | 29.8% | 21.6% |
| <=5 | 0 | Y | >3 | N | 1.6 | 76.3% | 40.1% | 27.0% | 19.1% |
| <=5 | 0 | Y | >3 | Y | 1.5 | 76.9% | 41.2% | 28.0% | 20.1% |
| <=5 | >=1 | N | 0-3 | N | 1.6 | 75.5% | 38.6% | 25.6% | 17.8% |
| <=5 | >=1 | N | 0-3 | Y | 1.6 | 76.1% | 39.7% | 26.6% | 18.7% |
| <=5 | >=1 | N | >3 | N | 1.7 | 74.4% | 36.8% | 23.9% | 16.4% |
| <=5 | >=1 | N | >3 | Y | 1.7 | 75.1% | 37.9% | 24.9% | 17.2% |
| <=5 | >=1 | Y | 0-3 | N | 2.5 | 65.5% | 23.9% | 12.8% | 7.5% |
| <=5 | >=1 | Y | 0-3 | Y | 2.4 | 66.3% | 24.9% | 13.6% | 8.0% |
| <=5 | >=1 | Y | >3 | N | 2.6 | 64.1% | 22.2% | 11.6% | 6.6% |
| <=5 | >=1 | Y | >3 | Y | 2.5 | 64.9% | 23.2% | 12.3% | 7.1% |
| >5 | 0 | N | 0-3 | N | 1.4 | 78.2% | 43.6% | 30.4% | 22.2% |
| >5 | 0 | N | 0-3 | Y | 1.4 | 78.8% | 44.6% | 31.4% | 23.2% |
| >5 | 0 | N | >3 | N | 1.5 | 77.3% | 41.8% | 28.6% | 20.6% |
| >5 | 0 | N | >3 | Y | 1.5 | 77.8% | 42.8% | 29.7% | 21.5% |
| >5 | 0 | Y | 0-3 | N | 2.2 | 69.1% | 28.6% | 16.6% | 10.4% |
| >5 | 0 | Y | 0-3 | Y | 2.1 | 69.8% | 29.6% | 17.5% | 11.1% |
| >5 | 0 | Y | >3 | N | 2.3 | 67.8% | 26.9% | 15.2% | 9.3% |
| >5 | 0 | Y | >3 | Y | 2.2 | 68.6% | 27.9% | 16.1% | 9.9% |
| >5 | >=1 | N | 0-3 | N | 2.4 | 66.7% | 25.4% | 14.1% | 8.4% |
| >5 | >=1 | N | 0-3 | Y | 2.3 | 67.5% | 26.5% | 14.9% | 9.0% |
| >5 | >=1 | N | >3 | N | 2.5 | 65.4% | 23.8% | 12.8% | 7.4% |
| >5 | >=1 | N | >3 | Y | 2.4 | 66.2% | 24.8% | 13.5% | 8.0% |
| >5 | >=1 | Y | 0-3 | N | 3.6 | 54.4% | 12.7% | 5.2% | 2.4% |
| >5 | >=1 | Y | 0-3 | Y | 3.5 | 55.3% | 13.5% | 5.7% | 2.7% |
| >5 | >=1 | Y | >3 | N | 3.7 | 52.8% | 11.5% | 4.5% | 2.0% |
| >5 | >=1 | Y | >3 | Y | 3.6 | 53.7% | 12.2% | 4.9% | 2.2% |
