# Supplementary material for: The prognostic role of inflammatory markers in patients with metastatic colorectal cancer treated with bevacizumab: A translational study [ASCENT]
Source: PLoS One. 2020 Mar 6;15(3):e0229900. doi: 10.1371/journal.pone.0229900 (PMC7059922; doi:10.1371/journal.pone.0229900)
Supplement: S2 Table — (DOCX) [file pone.0229900.s004.docx]

| **S2 Table: Predicted Overall Survival probabilities from the primary model (full analysis set)** | | | | | | | | | |
| --- | --- | --- | --- | --- | --- | --- | --- | --- | --- |
| Baseline Characteristics | | | | | Predicted OS probability | | | | |
| NLR | WHO Performance Status | Metastatic Disease of the liver | Number of site of Metastatic Disease | Presence of Metastatic Disease in the liver with no other sites involved | Hazard Ratio | 6 months | 1 yr | 1.5 yr | 2 yr |
| <=5 | 0 | N | 0-3 | N | Reference | 96.0% | 86.6% | 79.8% | 70.3% |
| <=5 | 0 | N | 0-3 | Y | 0.8 | 96.9% | 89.3% | 83.8% | 75.8% |
| <=5 | 0 | N | >3 | N | 1.5 | 94.0% | 80.1% | 70.6% | 58.1% |
| <=5 | 0 | N | >3 | Y | 1.2 | 95.2% | 84.0% | 76.1% | 65.3% |
| <=5 | 0 | Y | 0-3 | N | 1.4 | 94.5% | 81.6% | 72.7% | 60.8% |
| <=5 | 0 | Y | 0-3 | Y | 1.1 | 95.6% | 85.3% | 77.9% | 67.6% |
| <=5 | 0 | Y | >3 | N | 2.2 | 91.6% | 73.1% | 61.2% | 46.4% |
| <=5 | 0 | Y | >3 | Y | 1.7 | 93.3% | 78.2% | 68.0% | 54.8% |
| <=5 | >=1 | N | 0-3 | N | 1.8 | 93.1% | 77.4% | 66.9% | 53.4% |
| <=5 | >=1 | N | 0-3 | Y | 1.4 | 94.5% | 81.8% | 73.0% | 61.1% |
| <=5 | >=1 | N | >3 | N | 2.7 | 89.5% | 67.4% | 53.9% | 38.0% |
| <=5 | >=1 | N | >3 | Y | 2.1 | 91.7% | 73.4% | 61.5% | 46.8% |
| <=5 | >=1 | Y | 0-3 | N | 2.5 | 90.4% | 69.7% | 56.7% | 41.3% |
| <=5 | >=1 | Y | 0-3 | Y | 2.0 | 92.4% | 75.3% | 64.1% | 49.9% |
| <=5 | >=1 | Y | >3 | N | 3.9 | 85.6% | 57.3% | 41.8% | 25.6% |
| <=5 | >=1 | Y | >3 | Y | 3.0 | 88.5% | 64.6% | 50.4% | 34.3% |
| >5 | 0 | N | 0-3 | N | 1.6 | 93.6% | 78.9% | 68.9% | 55.9% |
| >5 | 0 | N | 0-3 | Y | 1.3 | 94.9% | 83.0% | 74.6% | 63.3% |
| >5 | 0 | N | >3 | N | 2.5 | 90.2% | 69.4% | 56.3% | 40.8% |
| >5 | 0 | N | >3 | Y | 2.0 | 92.3% | 75.0% | 63.7% | 49.5% |
| >5 | 0 | Y | 0-3 | N | 2.3 | 91.0% | 71.5% | 59.1% | 44.0% |
| >5 | 0 | Y | 0-3 | Y | 1.8 | 92.9% | 76.9% | 66.2% | 52.5% |
| >5 | 0 | Y | >3 | N | 3.6 | 86.5% | 59.7% | 44.5% | 28.2% |
| >5 | 0 | Y | >3 | Y | 2.8 | 89.3% | 66.7% | 53.0% | 37.0% |
| >5 | >=1 | N | 0-3 | N | 2.9 | 88.8% | 65.6% | 51.5% | 35.5% |
| >5 | >=1 | N | 0-3 | Y | 2.3 | 91.1% | 71.8% | 59.4% | 44.4% |
| >5 | >=1 | N | >3 | N | 4.5 | 83.3% | 52.2% | 36.0% | 20.3% |
| >5 | >=1 | N | >3 | Y | 3.5 | 86.7% | 60.0% | 44.9% | 28.6% |
| >5 | >=1 | Y | 0-3 | N | 4.1 | 84.6% | 55.1% | 39.3% | 23.2% |
| >5 | >=1 | Y | 0-3 | Y | 3.2 | 87.7% | 62.6% | 48.0% | 31.8% |
| >5 | >=1 | Y | >3 | N | 6.4 | 77.3% | 39.9% | 23.7% | 10.5% |
| >5 | >=1 | Y | >3 | Y | 5.0 | 81.7% | 48.7% | 32.3% | 17.1% |
