# Supplementary material for: The prognostic role of inflammatory markers in patients with metastatic colorectal cancer treated with bevacizumab: A translational study [ASCENT]
Source: PLoS One. 2020 Mar 6;15(3):e0229900. doi: 10.1371/journal.pone.0229900 (PMC7059922; doi:10.1371/journal.pone.0229900)
Supplement: S3 Table — (DOCX) [file pone.0229900.s005.docx]

| **S3 Table: Impact of potential confounders on primary endpoint (full analysis set)** |
| --- |

| Confounder |  | N | Events | Censored | Hazard Ratio  for NLR | 95% CI | Wald p-value  for NLR |
| --- | --- | --- | --- | --- | --- | --- | --- |
| None |  | 127 | 108 | 19 | 1.4 | (0.9 - 2.2) | 0.101 |
| Age |  | 127 | 108 | 19 | 1.4 | (0.9 - 2.2) | 0.105 |
| Sex |  | 127 | 108 | 19 | 1.5 | (0.9 - 2.3) | 0.088 |
| Charlson Comorbidity Index |  | 123 | 104 | 19 | 1.4 | (0.9 - 2.2) | 0.158 |
| Charlson Comorbidity Index:   <=1, >1 |  | 123 | 104 | 19 | 1.4 | (0.9 - 2.2) | 0.178 |
| ECG Results:   Normal, Abnormal NCS, Abnormal CS |  | 124 | 106 | 18 | 1.4 | (0.9 - 2.1) | 0.153 |
| Any Clinically significant Physical Examination:   No, Yes |  | 124 | 106 | 18 | 1.4 | (0.9 - 2.2) | 0.156 |
| BMI |  | 115 | 97 | 18 | 1.4 | (0.9 - 2.1) | 0.203 |
| PLR |  | 127 | 108 | 19 | 1.3 | (0.7 - 2.4) | 0.406 |
| PLR:   <=150, >150 and <=300, >300 |  | 127 | 108 | 19 | 1.2 | (0.6 - 2.4) | 0.653 |
| C-reactive protein and albumin:   C-reactive protein <= 10 mg/l and albumin >= 35 g/l ,   C-reactive protein <= 10 mg/l and albumin < 35 g/l ,   C-reactive protein > 10 mg/l ,   C-reactive protein > 10 mg/l and albumin < 35 g/l |  | 113 | 94 | 19 | 1.1 | (0.7 - 1.9) | 0.699 |
| Glasgow Prognostic Index |  | 127 | 108 | 19 | 0.7 | (0.4 - 1.4) | 0.299 |
| Absolute Neutrophil Count (10^9/L) |  | 112 | 95 | 17 | 1.2 | (0.7 - 2.0) | 0.607 |
| Activated Partial Thromboplastin Time (sec) |  | 102 | 85 | 17 | 1.5 | (0.9 - 2.5) | 0.126 |
| Alanine Aminotransferase (IU/L) |  | 126 | 107 | 19 | 1.5 | (0.9 - 2.3) | 0.089 |
| Albumin (g/L) |  | 126 | 107 | 19 | 1.3 | (0.8 - 2.1) | 0.231 |
| Alkaline Phosphatase (IU/L) |  | 126 | 107 | 19 | 1.1 | (0.6 - 1.8) | 0.737 |
| Aspartate Aminotransferase (IU/L) |  | 127 | 108 | 19 | 1.2 | (0.8 - 1.9) | 0.424 |
| Basophils (10^9/L) |  | 127 | 108 | 19 | 1.5 | (1.0 - 2.3) | 0.081 |
| Bilirubin (umol/L) |  | 126 | 107 | 19 | 1.4 | (0.9 - 2.2) | 0.115 |
| Blood Urea Nitrogen (mmol/L) |  | 121 | 103 | 18 | 1.5 | (1.0 - 2.4) | 0.081 |
| C Reactive Protein (mg/dL) |  | 113 | 94 | 19 | 1.0 | (0.6 - 1.7) | 0.997 |
| Calcium (mmol/L) |  | 120 | 103 | 17 | 1.4 | (0.9 - 2.2) | 0.156 |
| Carcinoembryonic Antigen (ug/L) |  | 121 | 103 | 18 | 1.4 | (0.9 - 2.2) | 0.170 |
| Chloride (mmol/L) |  | 125 | 106 | 19 | 1.2 | (0.7 - 2.0) | 0.449 |
| Creatinine (umol/L) |  | 126 | 107 | 19 | 1.4 | (0.9 - 2.2) | 0.154 |
| Creatinine Clearance (mL/min) |  | 115 | 97 | 18 | 1.4 | (0.9 - 2.2) | 0.167 |
| Eosinophils (10^9/L) |  | 127 | 108 | 19 | 1.5 | (1.0 - 2.4) | 0.055 |
| Erythrocytes (10^12/L) |  | 127 | 108 | 19 | 1.4 | (0.9 - 2.2) | 0.188 |
| Gamma Glutamyl Transferase (IU/L) |  | 126 | 107 | 19 | 1.2 | (0.7 - 2.0) | 0.419 |
| Glucose (mmol/L) |  | 112 | 96 | 16 | 1.3 | (0.8 - 2.1) | 0.237 |
| Hemoglobin (g/L) |  | 127 | 108 | 19 | 1.3 | (0.8 - 2.0) | 0.305 |
| Lactate Dehydrogenase (IU/L) |  | 119 | 100 | 19 | 1.3 | (0.8 - 2.2) | 0.315 |
| Leukocytes (10^9/L) |  | 127 | 108 | 19 | 1.2 | (0.8 - 2.0) | 0.356 |
| Lymphocytes (10^9/L) |  | 127 | 108 | 19 | 1.6 | (0.9 - 2.7) | 0.108 |
| Monocytes (10^9/L) |  | 127 | 108 | 19 | 1.2 | (0.8 - 2.0) | 0.354 |
| Neutrophils (10^9/L) |  | 127 | 108 | 19 | 1.1 | (0.7 - 1.8) | 0.651 |
| Platelets (10^9/L) |  | 127 | 108 | 19 | 1.2 | (0.8 - 1.9) | 0.379 |
| Potassium (mmol/L) |  | 125 | 106 | 19 | 1.4 | (0.9 - 2.2) | 0.137 |
| Protein (g/L) |  | 124 | 106 | 18 | 1.4 | (0.9 - 2.1) | 0.178 |
| Prothrombin Intl. Normalized Ratio (ratio) |  | 111 | 95 | 16 | 1.4 | (0.9 - 2.3) | 0.182 |
| Sodium (mmol/L) |  | 126 | 107 | 19 | 1.3 | (0.8 - 2.0) | 0.315 |
| Urate (umol/L) |  | 97 | 84 | 13 | 1.4 | (0.8 - 2.4) | 0.217 |
| pH |  | 114 | 98 | 16 | 1.3 | (0.8 - 2.0) | 0.269 |
