# Supplementary material for: The prognostic role of inflammatory markers in patients with metastatic colorectal cancer treated with bevacizumab: A translational study [ASCENT]
Source: PLoS One. 2020 Mar 6;15(3):e0229900. doi: 10.1371/journal.pone.0229900 (PMC7059922; doi:10.1371/journal.pone.0229900)
Supplement: S4 Table — (DOCX) [file pone.0229900.s006.docx]

| **S4 Table. The association between baseline proteomic markers and PFS (full analysis set)** | | | | | | | | |
| --- | --- | --- | --- | --- | --- | --- | --- | --- |
| Model | Parameter | N | Level | Hazard Ratio | 95% CI for  Hazard Ratio | p-value for  model | p-value for  variable | p-value for  level |
| 1 | NLR | 39 | <=5 | Reference | NA | 0.407 | 0.395 | NA |
|  |  | 12 | >5 | 1.35 | 0.68 - 2.68 |  |  | 0.395 |
| 2 | **A1AGLP BASELINE** | **51** |  | **1.13** | **1.00 - 1.28** | **0.119** | **0.047** |  |
|  | NLR | 39 | <=5 | Reference | NA |  | 0.665 | NA |
|  |  | 12 | >5 | 1.17 | 0.58 - 2.35 |  |  | 0.665 |
| 3 | A1ANTRYP BASELINE | 51 |  | 1.17 | 0.92 - 1.50 | 0.321 | 0.198 |  |
|  | NLR | 39 | <=5 | Reference | NA |  | 0.453 | NA |
|  |  | 12 | >5 | 1.30 | 0.65 - 2.59 |  |  | 0.453 |
| 4 | **A1MICG BASELINE** | **51** |  | **0.09** | **0.01 - 0.99** | **0.075** | **0.049** |  |
|  | NLR | 39 | <=5 | Reference | NA |  | 0.900 | NA |
|  |  | 12 | >5 | 0.95 | 0.45 - 2.03 |  |  | 0.900 |
| 5 | A2MACG BASELINE | 51 |  | 1.01 | 0.84 - 1.22 | 0.701 | 0.877 |  |
|  | NLR | 39 | <=5 | Reference | NA |  | 0.391 | NA |
|  |  | 12 | >5 | 1.37 | 0.67 - 2.81 |  |  | 0.391 |
| 6 | **AACT BASELINE** | **51** |  | **1.62** | **1.04 - 2.53** | **0.095** | **0.033** |  |
|  | NLR | 39 | <=5 | Reference | NA |  | 0.705 | NA |
|  |  | 12 | >5 | 0.85 | 0.38 - 1.94 |  |  | 0.705 |
| 7 | ANGTNSGN BASELINE | 51 |  | 1.75 | 0.14 - 21.72 | 0.648 | 0.664 |  |
|  | NLR | 39 | <=5 | Reference | NA |  | 0.395 | NA |
|  |  | 12 | >5 | 1.35 | 0.68 - 2.68 |  |  | 0.395 |
| 8 | ANTHRM BASELINE | 51 |  | 0.34 | 0.07 - 1.63 | 0.286 | 0.176 |  |
|  | NLR | 39 | <=5 | Reference | NA |  | 0.774 | NA |
|  |  | 12 | >5 | 1.11 | 0.53 - 2.32 |  |  | 0.774 |
| 9 | APOA1 BASELINE | 51 |  | 0.84 | 0.63 - 1.11 | 0.316 | 0.215 |  |
|  | NLR | 39 | <=5 | Reference | NA |  | 0.675 | NA |
|  |  | 12 | >5 | 1.17 | 0.57 - 2.41 |  |  | 0.675 |
| 10 | APOA4 BASELINE | 51 |  | 0.15 | 0.02 - 1.10 | 0.112 | 0.061 |  |
|  | NLR | 39 | <=5 | Reference | NA |  | 0.991 | NA |
|  |  | 12 | >5 | 1.00 | 0.48 - 2.11 |  |  | 0.991 |
| 11 | APOB BASELINE | 51 |  | 1.27 | 0.20 - 7.91 | 0.687 | 0.798 |  |
|  | NLR | 39 | <=5 | Reference | NA |  | 0.377 | NA |
|  |  | 12 | >5 | 1.37 | 0.68 - 2.77 |  |  | 0.377 |
| 12 | **APOC3 BASELINE** | **51** |  | **0.77** | **0.60 - 1.00** | **0.085** | **0.049** |  |
|  | NLR | 39 | <=5 | Reference | NA |  | 0.871 | NA |
|  |  | 12 | >5 | 1.06 | 0.52 - 2.18 |  |  | 0.871 |
| 13 | APOJ BASELINE | 51 |  | 0.58 | 0.17 - 2.06 | 0.494 | 0.402 |  |
|  | NLR | 39 | <=5 | Reference | NA |  | 0.769 | NA |
|  |  | 12 | >5 | 1.13 | 0.51 - 2.51 |  |  | 0.769 |
| 14 | C3 BASELINE | 51 |  | 1.33 | 0.54 - 3.26 | 0.588 | 0.534 |  |
|  | NLR | 39 | <=5 | Reference | NA |  | 0.325 | NA |
|  |  | 12 | >5 | 1.43 | 0.70 - 2.92 |  |  | 0.325 |
| 15 | C4A BASELINE | 51 |  | 1.51 | 0.83 - 2.77 | 0.296 | 0.179 |  |
|  | NLR | 39 | <=5 | Reference | NA |  | 0.189 | NA |
|  |  | 12 | >5 | 1.67 | 0.78 - 3.57 |  |  | 0.189 |
| 16 | CBG BASELINE | 51 |  | 0.14 | 0.00 - 73.66 | 0.587 | 0.540 |  |
|  | NLR | 39 | <=5 | Reference | NA |  | 0.416 | NA |
|  |  | 12 | >5 | 1.33 | 0.67 - 2.65 |  |  | 0.416 |
| 17 | CFAH BASELINE | 51 |  | 7.14 | 0.10 - 493.89 | 0.472 | 0.363 |  |
|  | NLR | 39 | <=5 | Reference | NA |  | 0.268 | NA |
|  |  | 12 | >5 | 1.52 | 0.73 - 3.18 |  |  | 0.268 |
| 18 | **CRLPLSMN BASELINE** | **51** |  | **14.65** | **1.06 - 202.32** | **0.103** | **0.045** |  |
|  | NLR | 39 | <=5 | Reference | NA |  | 0.555 | NA |
|  |  | 12 | >5 | 1.23 | 0.62 - 2.46 |  |  | 0.555 |
| 19 | CRP BASELINE | 51 |  | 380.47 | 9.79 - 14791.84 | 0.010 | 0.001 |  |
|  | NLR | 39 | <=5 | Reference | NA |  | 0.584 | NA |
|  |  | 12 | >5 | 0.80 | 0.36 - 1.77 |  |  | 0.584 |
| 20 | **CRP_LOG BASELINE** | **51** |  | **1.48** | **1.09 - 2.02** | **0.026** | **0.013** |  |
|  | NLR | 39 | <=5 | Reference | NA |  | 0.761 | NA |
|  |  | 12 | >5 | 0.89 | 0.41 - 1.91 |  |  | 0.761 |
| 21 | FACTII BASELINE | 51 |  | 0.15 | 0.01 - 1.70 | 0.199 | 0.125 |  |
|  | NLR | 39 | <=5 | Reference | NA |  | 0.895 | NA |
|  |  | 12 | >5 | 1.05 | 0.50 - 2.22 |  |  | 0.895 |
| 22 | FIBA BASELINE | 51 |  | 0.92 | 0.81 - 1.06 | 0.361 | 0.252 |  |
|  | NLR | 39 | <=5 | Reference | NA |  | 0.352 | NA |
|  |  | 12 | >5 | 1.39 | 0.70 - 2.77 |  |  | 0.352 |
| 23 | **FIBB BASELINE** | **51** |  | **0.80** | **0.67 - 0.96** | **0.032** | **0.014** |  |
|  | NLR | 39 | <=5 | Reference | NA |  | 0.463 | NA |
|  |  | 12 | >5 | 1.29 | 0.65 - 2.58 |  |  | 0.463 |
| 24 | FIBG BASELINE | 51 |  | 0.58 | 0.30 - 1.14 | 0.180 | 0.115 |  |
|  | NLR | 39 | <=5 | Reference | NA |  | 0.220 | NA |
|  |  | 12 | >5 | 1.57 | 0.76 - 3.21 |  |  | 0.220 |
| 25 | HEMO BASELINE | 51 |  | 0.91 | 0.71 - 1.17 | 0.529 | 0.445 |  |
|  | NLR | 39 | <=5 | Reference | NA |  | 0.477 | NA |
|  |  | 12 | >5 | 1.29 | 0.64 - 2.58 |  |  | 0.477 |
| 26 | HPTA BASELINE | 51 |  | 1.03 | 0.93 - 1.13 | 0.615 | 0.594 |  |
|  | NLR | 39 | <=5 | Reference | NA |  | 0.500 | NA |
|  |  | 12 | >5 | 1.28 | 0.63 - 2.61 |  |  | 0.500 |
| 27 | HPTB BASELINE | 51 |  | 1.10 | 0.99 - 1.23 | 0.118 | 0.062 |  |
|  | NLR | 39 | <=5 | Reference | NA |  | 0.697 | NA |
|  |  | 12 | >5 | 1.15 | 0.57 - 2.32 |  |  | 0.697 |
| 28 | **KNG1 BASELINE** | **51** |  | **0.00** | **0.00 - 0.49** | **0.054** | **0.024** |  |
|  | NLR | 39 | <=5 | Reference | NA |  | 0.878 | NA |
|  |  | 12 | >5 | 1.06 | 0.52 - 2.15 |  |  | 0.878 |
| 29 | PON1 BASELINE | 51 |  | 0.29 | 0.06 - 1.35 | 0.193 | 0.115 |  |
|  | NLR | 39 | <=5 | Reference | NA |  | 0.644 | NA |
|  |  | 12 | >5 | 1.18 | 0.58 - 2.38 |  |  | 0.644 |
| 30 | PON1_LOG BASELINE | 51 |  | 0.58 | 0.31 - 1.09 | 0.170 | 0.091 |  |
|  | NLR | 39 | <=5 | Reference | NA |  | 0.695 | NA |
|  |  | 12 | >5 | 1.15 | 0.57 - 2.35 |  |  | 0.695 |
| 31 | **PREALB BASELINE** | **51** |  | **0.01** | **0.00 - 0.59** | **0.045** | **0.027** |  |
|  | NLR | 39 | <=5 | Reference | NA |  | 0.808 | NA |
|  |  | 12 | >5 | 1.09 | 0.54 - 2.22 |  |  | 0.808 |
| 32 | SAA BASELINE | 51 |  | 2.03 | 0.99 - 4.18 | 0.176 | 0.054 |  |
|  | NLR | 39 | <=5 | Reference | NA |  | 0.713 | NA |
|  |  | 12 | >5 | 1.14 | 0.56 - 2.31 |  |  | 0.713 |
| 33 | TRFE BASELINE | 51 |  | 0.65 | 0.36 - 1.17 | 0.260 | 0.153 |  |
|  | NLR | 39 | <=5 | Reference | NA |  | 0.809 | NA |
|  |  | 12 | >5 | 1.10 | 0.52 - 2.30 |  |  | 0.809 |
| 34 | VTDB BASELINE | 51 |  | 3.72 | 0.47 - 29.45 | 0.341 | 0.213 |  |
|  | NLR | 39 | <=5 | Reference | NA |  | 0.217 | NA |
|  |  | 12 | >5 | 1.59 | 0.76 - 3.34 |  |  | 0.217 |
| 35 | VTNC BASELINE | 51 |  | 0.72 | 0.45 - 1.15 | 0.256 | 0.170 |  |
|  | NLR | 39 | <=5 | Reference | NA |  | 0.676 | NA |
|  |  | 12 | >5 | 1.16 | 0.57 - 2.37 |  |  | 0.676 |
